# A new multi-attribute group decision-making method based on Einstein Bonferroni operators under interval-valued Fermatean hesitant fuzzy environment

**DOI:** 10.1038/s41598-024-62762-0

**Published:** 2024-05-29

**Authors:** Siyue Lei, Xiuqin Ma, Hongwu Qin, Yibo Wang, Jasni Mohamad Zain

**Affiliations:** 1 College of Computer Science and Engineering, Northwest Normal University, Lanzhou, 730070 Gansu China; 2 Institute for Big Data Analytics and Artificial Intelligence (IBDAAI), Universiti Teknologi MARA, 40450 Shah Alam, Malaysia

**Keywords:** Interval-valued Fermatean hesitant fuzzy sets, Einstein t-norms, Bonferroni mean, Multi-attribute group decision-making, Applied mathematics, Computational science, Computer science, Information technology, Pure mathematics, Scientific data, Software, Statistics

## Abstract

Faced with the increasing complexity and uncertainty of decision-making information, interval-valued Fermatean hesitant fuzzy sets (IVFHFSs) were presented as a novel mathematical model that handled uncertain data more effectively. However, existing multi-attribute group decision-making (MAGDM) methods based on IVFHFSs do not thoroughly investigate the operational laws. Also, these existing MAGDM methods do not take into account the connections between attributes and are less flexible. To address these issues, this paper proposes a new MAGDM method based on Einstein Bonferroni operators under IVFHFSs. First, we thoroughly examine the operational laws of Einstein t-norms under the IVFHFSs to further extend the study of the operational laws. Then, we introduce the interval-valued Fermatean hesitant fuzzy Einstein Bonferroni mean operator and the interval-valued Fermatean hesitant fuzzy Einstein weighted Bonferroni mean operator under Einstein t-norms. Our suggested aggregation operators consider the relationship between attributes and are far more flexible in comparison to the current approaches. Later, a novel MAGDM method based on Einstein Bonferroni operators under the IVFHFSs is given. Finally, the practicality and validity of the proposed method are demonstrated by a cardiovascular disease diagnosis application.

## Introduction

Choosing the best choice from a group of objects on the basis of a variety of qualitative and quantitative attributes is known as multi-attribute decision-making (MADM)^1,2^. As a well-known outflow of decision theory, MADM has been substantially explored and successfully applied to a variety of domains, including industries, medical diagnosis, engineering and environmental sciences, and so on^3,4^. Furthermore, when faced with a particularly important task in practice, the decision will be taken by a group of decision-makers who are going to address it together. As a result, the concept of multi-attribute group decision-making (MAGDM) was established, with the intention of having several decision-makers choose the best option from a group of alternatives. In recent years, some MAGDM methods have been developed^5–8^. However, in practice, decision-makers are frequently challenged with uncertain and fuzzy information when performing MAGDM.

**Figure 1:**
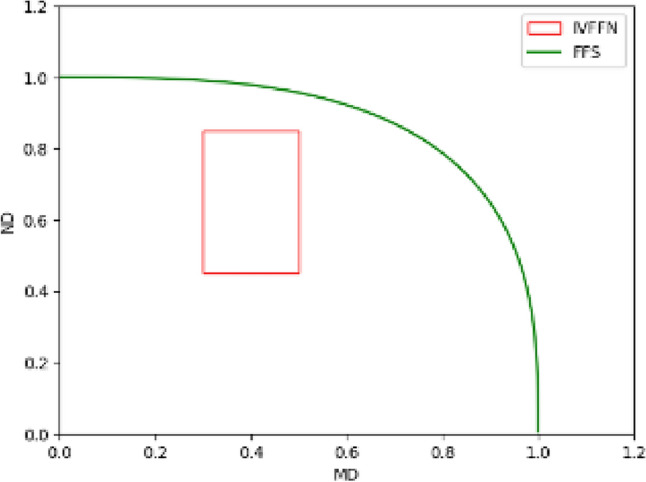
Diagram of an IVFFS containing only one fuzzy number.

**Figure 2:**
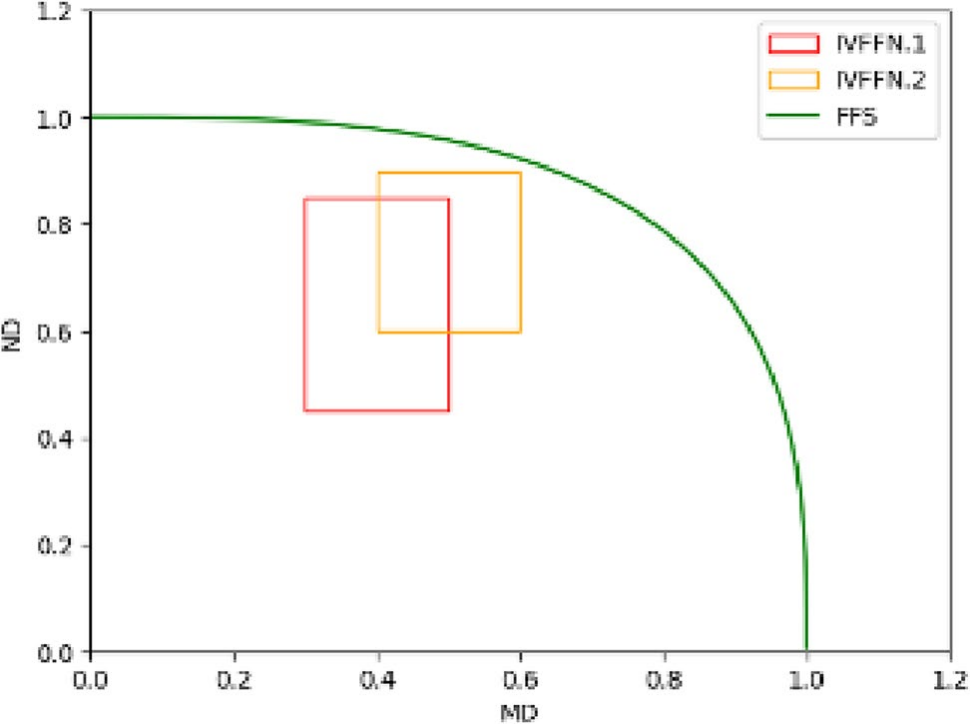
Diagram of an IVFHFS containing only one fuzzy number with hesitation number of two.

Uncertain information^9^ in the actual world has increased due to the decision-making environment's rising complexity^10^. In 1965, Zadeh^11^ proposed the fuzzy sets (FSs) theory as a solution to the uncertainty problem. Then, Atanassov^12^ proposed the intuitionistic fuzzy sets (IFSs), which supplemented the concept of “membership degree(MD)” with “non-membership degree(ND)” and $0\le MD+ND\le 1$. Further, the idea of interval-valued intuitionistic fuzzy sets (IVIFSs)^13^ were put forth by extending the IFSs, which enables the decision maker to describe the evaluation range of an alternative scheme on a particular attribute using an interval number^14^. Interval-valued Pythagorean fuzzy sets (IVPFSs) were proposed in^15^, which were inspired by IVIFSs and incorporated the feature of interval numbers into Pythagorean fuzzy sets. By limiting the sum of the squares of the upper bound of the MD and ensuring that the upper bound of the ND does not exceed 1, IVPFSs can handle more fuzzy information than IVIFSs. In 2022, Rani and Mishra^16^ proposed the conception of interval-valued Fermatean fuzzy sets (IVFFSs) by extending IVPFSs. Compared with IVIFSs and IVPFSs, the sum of the cubic MD and the cubic ND of IVFFS does not exceed 1, which can describe a wider range of fuzzy information. Figure 1 indicates the range of uncertain information that can be represented when there is only one element in IVFFSs. It might be challenging to come to an agreement on assessments when decision-makers are reluctant to use assessments in complex and unclear situations such as mental health evaluations. Consequently, the concept of hesitant fuzzy sets (HFSs) was first developed in 2009 by Torra et al.^17^ as an extension of FSs. A group of likely values serves as the representation of the MD of HFSs, which is suitable for describing hesitant and uncertain information. As a result, HFSs have been thoroughly studied and developed recently, and some extended models based on HFSs were given as diverse as dual hesitant fuzzy sets^18^, dual hesitant Pythagorean fuzzy sets^19^, interval-valued hesitant fuzzy sets^20^ and Fermatean hesitant fuzzy sets (FHFSs)^21^ and so on. Among these above-extended HFS, interval-valued Fermatean hesitant fuzzy sets (IVFHFSs) are one of the most worthy of attention developed by Kirişci and Şimşek^22^ in 2022. The model of IVFHFSs is the extension of FHFSs and IVFFSs, which inherit their strengths. That is, IVFHFSs not only adopt interval-valued data to describe MD and ND with a wider range but also involve the hesitant feature of data. Figure 2 represents the range of uncertain information that can be represented with only one element in IVFHFSs when the number of hesitations for that element is 2. From Fig. 2, we can understand that IVFHFSs fully consider the advantages of FHFSs and IVFFSs. It allows us to have a wider, more flexible, and more clever range in representing uncertain information by adjusting the range and number of the frames in Fig. 2.

The aggregation of fuzzy information is essential for solving the MAGDM problem in the fuzzy environment. As a result, research on aggregation operators (AOs) is very important and valuable and has been developed and studied by many researchers in recent years. The most common approach to fuzzy aggregation operations in works on AOs up to this point is the combination of the fundamental algebraic product and the algebraic sum of Archimedean t-norms (AR-TNs)^23,24^. The class of strictly AR-TNs is known to include, among other examples, algebraic t-norms (A-TNs) and Einstein t-norms (E-TNs)^25–27^. The algebraic product and sum are similar to Einstein product and sum in the representation of smoothing approximations. As a result, E-TNs are good substitutes for A-TNs. These operators provide us with a wide range of MADM applications while eliminating the irrationality and inconsistent nature of the operational laws. Rani et al.^28^ pointed out that the algebraic sum and product operations do not perform as well as the Einstein sum and product under the intersection and union of Fermatean fuzzy sets (FFSs). They made the point that compared to basic operations, E-TNs operations are more valuable and flexible. Rahman et al.^29^ proposed Einstein weighted averaging AO and Einstein ordered weighted averaging AO under the IVPFSs environment. To solve supply chain management problems, Ali et al.^30^ studied a series of complex Einstein weighted geometric AOs based on IVPFSs. Based on the FFSs environment, Rani and Mishra^31^ proposed some Einstein AOs and demonstrated the effectiveness and advantages of the approach through the multi-criteria electric vehicle charging station problems. Bonferroni mean(BM)^32^ as a mean operator is also one of the most popular AOs. In 2009, Yager^33^ used the BM operator to deal with the multi-criteria problem. Later, Beliakov et al.^34^ studied the generalized BM operators in detail and solved the defect that the BM operator just only handles exact numbers. Zhu and Xu^35^ extended the BM operator to HFSs. Xu et al.^36^ explored the Pythagorean fuzzy BM operator and developed an accelerative calculating algorithm for it. Wang et al.^37^ introduced the BM operator into the hesitant Fermatean fuzzy sets to tackle the MADM problem. Ali et al.^38^ devised Aczel-Alsina operations to interval-valued q-rung orthopair fuzzy sets and originated a series of aggregation operators, including the BM operator. Based on dynamic comprehensive time entropy and an ATS-generalized weighted intuitionistic fuzzy BM operator, Zhang et al.^39^ established a new MADM model to make decisions about attributes and time weights present in dynamic intuitionistic fuzzy environments.

In recent years, the research method of MAGDM^40,41^ combined with fuzzy set theory has developed by leaps and bounds in various fields. At present, the extensive application of this method in the medical field is especially outstanding. Here is a good case to reveal the positive effects of this approach. The application of the MAGDM method drastically reduces the mortality rate from cardiovascular diseases, which pose a serious threat to human health. The World Health Organization (WHO) reported that 18 million deaths worldwide are attributed to cardiovascular diseases^42^. Data from the National Bureau of Statistics (https://data.stats.gov.cn/) shows that the proportion of deaths due to cardiovascular disease in the total number of deaths is increasing year by year and has reached 23.65% in 2019. Therefore, early prevention and control of these diseases is very necessary. Early monitoring, early diagnosis, and early treatment can not only effectively reduce the morbidity and mortality of such diseases but also improve the quality of life for patients. Accelerating the diagnosis of these diseases and improving the degree of effective diagnosis has become one of the key methods to reduce the harm of these diseases. Nowadays, the clinical diagnosis of cardiovascular disease is made by doctors who analyze clinical tests to determine the degree of ambiguity of a patient's cardiovascular disease. This judgment information is typically uncertain. This is because we can only make a vague judgment about the likelihood of a patient having a cardiovascular disease based on clinical tests, but we cannot make a definitive judgment about whether the patient has cardiovascular disease. In comparison with other fuzzy set models, we find that IVFHFSs are particularly suitable for the medical diagnosis of cardiovascular disease due to their hesitant nature. IVFHFSs can combine the opinions of all medical experts without data loss, and they are more accurate and flexible in representing the opinions of medical experts. For the moment, both Kirişci et al.^22^ and DemİR et al.^43^ have applied IVFHFSs to the medical field. Kirişci et al.^22^ initiated the model of IVFHFSs and proposed various related AOs, such as the interval-valued Fermatean hesitant weighted averaging operator and interval-valued Fermatean geometric operator, and the new score function (SC) was proposed to rank the alternatives. Then, the validity and feasibility of the proposed MAGDM method were verified under medical decision-making application. DemİR et al.^43^ have proposed the correlation coefficients and weighted correlation coefficients methods based on the IVFHFSs. Then, the viability and usefulness were demonstrated through pattern recognition application and medical decision-making.

However, there are some research gaps as follows. That is, we find that there are still some shortcomings in existing MAGDM methods^22,43,44^. Firstly, the AOs proposed by Kirişci et al.^22^ and Zeng et al.^44^ have very low flexibility, and both are based on A-TNs. However, E-TNs are better substitutions for A-TNs, and there is no research on Einstein AOs for IVFHFSs. Furthermore, the data model in^44^ is interval-valued hesitant fuzzy sets (IVHFSs). In fields with high complexity and uncertainty, such as the medical field, IVFHSs are unable to represent more information compared to IVFHFSs. Then, the methods in^22,43,44^ do not take into account the interconnections between attributes. In general, the attribute indicators in the medical field are related to each other in some way and can influence each other and their methods do not have adjustable parameters and cannot handle variable environments. In addition, when considering the opinions of the different experts, their methods do not take full advantage of the hesitant property of the data model, thus resulting in the loss of information. To address these issues mentioned above, we develop a new MAGDM approach based on the Einstein Bonferroni mean AOs under the IVFHFSs environment. The main innovation points are summarized as follows:

- Based on E-TNs, this paper studies the Einstein operators for IVFHFSs, enriching the research on aggregation operators under the IVFHFSs environment.
- By combining Einstein operators with the BM, this paper presents the interval-valued Fermatean hesitant fuzzy Einstein Bonferroni mean (IVFHFEBM) operator and the interval-valued Fermatean hesitant fuzzy Einstein weighted Bonferroni mean (IVFHFEWBM) operator. Compared with the existing methods, our proposed AOs take into account the connection between attributes. Our proposed operators have much higher flexibility in contrast to the existing operators based on IVFHFSs.
- This paper suggests a MAGDM approach using the IVFHFEBM and IVFHFEWBM operators. When dealing with multiple expert opinions, we take the approach of seeking common ground. This approach makes full use of the property of IVFHFSs to reduce the loss of information in the MAGDM. The rationality, validity, and superiority of the proposed methods are verified by a cardiac diagnosis application.

The following is the structure of the entire paper. "Preliminary" section briefly describes the basic concepts of partial fuzzy sets, including HFSs, FHFSs, IVFFSs, IVFHFSs, AR-TNs, and BM operation. The E-TNs operations with some desirable properties and the form and derivation of the IVFHFEBM and IVFHFEWBM are introduced in "The IVFHFEBM and IVFHFEWBM AOs under E-TNs" section. "A new MAGDM based on IVFHFEBM and IVFHFEWBM" section presents a new MAGDM method based on IVFHFEBM and IVFHFEWBM. "Case study and comparative analysis" section uses a case study of cardiac diagnostics to show the rationality and applicability of our methodology and demonstrates the robustness of our proposed method by conducting a parametric analysis, in addition to a comparison with existing decision-making methods to demonstrate the superiority of our proposed method. In "Conclusion" section, a summary is presented.

## Preliminary

This section provides a brief overview of the definitions of the HFSs, FHFSs, IVFFSs, IVFHFSs, AR-TNs, and BM operation.

### Definition 2.1.

^17^ Let $S \ne \phi$ and a HFS $H$ on $S$ is a function, and the mathematical is expressed as follows:

$$H = \left\{ { < s,h\left( s \right)\left| {s \in S} \right\rangle } \right\}$$

where $h\left( s \right)$ includes several values in $\left[ {0,1} \right]$, indicating the possible MD. We call the $h\left( s \right)$ as a hesitant fuzzy number.

### Definition 2.2.

^21^ Let $S \ne \phi$ and then a FHFS $\overline{FH}$ on $S$ can be represented by a function $h_{{\overline{FH} }} \left( s \right)$, individually, denoted by the mathematical notation that can be expressed as follows:

$$\begin{array}{*{20}c} {\overline{FH} = \left\{ { < s,\left( {h_{{\overline{FH} }} \left( s \right)} \right){|}s \in S > } \right\}} \\ \end{array}$$

where $h_{{\overline{FH} }} \left( s \right) :S \to \left[ {0,1} \right]$ indicates multiple possible pairs of MD ($u$) and ND ($\nu$) of $s$, $s \in S$. In general, we call $\overline{fh} = h_{{\overline{FH} }} \left( s \right) = \left( {\mu_{{\overline{FHi} }} \left( s \right),\nu_{{\overline{FHi} }} \left( s \right) } \right)$ as a Fermatean hesitant fuzzy number (FHFN), where $i$ indicates the number of FFNs. If $\alpha \in \overline{fh}$, then $\alpha$ is a FHFN, and it can be denoted by $\alpha = \left( {u,\nu } \right)$, and $0 < u^{3} + \nu^{3} \le 1$.

### Definition 2.3.

^16^ Let $S \ne \phi$ and then an IVFFS $\tilde{F}$ on $S$ is expressed as follows:

$$\tilde{F} = \left\{ { < s,([\mu_{{\tilde{F}}}^{ - } \left( s \right),\mu_{{\tilde{F}}}^{ + } \left( s \right)\left] {,[\nu_{{\tilde{F}}}^{ - } \left( s \right),\nu_{{\tilde{F}}}^{ + } \left( s \right)} \right]){|}s \in S > } \right\}$$

where $\mu_{{\tilde{F}}}^{ - } \left( s \right)$ and $\mu_{{\tilde{F}}}^{ + } \left( s \right)$ denote minimum and maximum values of interval-valued MD, respectively. Equally, $\nu_{{\tilde{F}}}^{ - } \left( s \right)$ and $\nu_{{\tilde{F}}}^{ + } \left( s \right)$ denote minimum and maximum values of interval-valued ND, separately, with the condition $0 \le \mu_{{\tilde{F}}}^{ - } \left( s \right) \le \mu_{F}^{ + } \left( s \right) \le 1$, $0 \le \nu_{{\tilde{F}}}^{ - } \left( s \right) \le \nu_{{\tilde{F}}}^{ + } \left( s \right) \le 1$, $0 < (\mu_{{\tilde{P}}}^{ + } )^{3} + (\nu_{{\tilde{P}}}^{ + } )^{3} \le 1$. For convenience, we call $\tilde{f} = ([\mu_{{\tilde{F}}}^{ - } \left( s \right),\mu_{{\tilde{F}}}^{ + } \left( s \right)\left] {,[\nu_{{\tilde{F}}}^{ - } \left( s \right),\nu_{{\tilde{F}}}^{ + } \left( s \right)} \right])$ as an interval-valued Fermatean fuzzy number (IVFFN). Specifically, when $\mu_{F}^{ - } \left( s \right) = \mu_{{\tilde{F}}}^{ + } \left( s \right)$ and $\nu_{{\tilde{F}}}^{ - } \left( s \right) = \nu_{F}^{ + } \left( s \right)$, the IVFFN is degraded to FFN.

For any parameter $s \in S$, the indeterminacy degree can be computed as $\pi_{{\tilde{F}}} \left( s \right) = \left[ {\pi_{{\tilde{F}}}^{ - } \left( s \right),\pi_{{\tilde{F}}}^{ + } \left( s \right)} \right] = \left[ {\sqrt[3]{{1 - (\mu_{{\tilde{F}}}^{ + } )^{3} - (\nu_{{\tilde{F}}}^{ + } )^{3} }},\sqrt[3]{{1 - (\mu_{{\tilde{F}}}^{ - } )^{3} - (\nu_{{\tilde{F}}}^{ - } )^{3} }}} \right]$. The degree of indeterminacy, the more indecisive the object is implied to be.

### Definition 2.4.

^22^ Let $S \ne \phi$ and an IVFHFS ${\mathcal{F}}$ on S is performed as follows:

$${\mathcal{F}} = \left\{ { < s,\left( {h_{{\mathcal{F}}} \left( s \right)} \right){|}s \in S > } \right\}$$

where $h_{{\mathcal{F}}} \left( s \right) :S \to \left[ {0,1} \right]$ denotes the multiple possible pair of interval-valued MD ($[\mu_{{\mathcal{F}}}^{ - } \left( s \right),\mu_{{\mathcal{F}}}^{ + } \left( s \right)]$) and ND ($[\nu_{{\mathcal{F}}}^{ - } \left( s \right),\nu_{{\mathcal{F}}}^{ + } \left( s \right)]$), satisfying all the $\mu_{{\mathcal{F}}}^{ - } \left( s \right)$, $\mu_{{\mathcal{F}}}^{ + } \left( s \right)$, $\nu_{{\mathcal{F}}}^{ - } \left( s \right)$ and $\nu_{{\mathcal{F}}}^{ + } \left( s \right)$ with the condition $0 \le \mu_{{\mathcal{F}}}^{ - } \left( s \right) \le \mu_{{\mathcal{F}}}^{ + } \left( s \right) \le 1$, $0 \le \nu_{{\mathcal{F}}}^{ - } \left( s \right) \le \nu_{{\mathcal{F}}}^{ + } \left( s \right) \le 1$, $0 < (\mu_{{\mathcal{F}}}^{ + } )^{3} + (\nu_{{\mathcal{F}}}^{ + } )^{3} \le 1$. As a rule, we call ${\mathcalligra{f}}_{x} = ([\mu_{xi}^{ - } ,\mu_{xi}^{ + } \left] {,[\nu_{xi}^{ - } ,\nu_{xi}^{ + } } \right]) = h_{{\mathcal{F}}} \left( s \right)$ as an interval-valued Fermatean hesitant fuzzy number (IVFHFN), where $x$ denotes the number of IVFHFN and $i$ implies the number of the combination of interval-valued MD and ND.

### Example 1.

There are two IVFHFNs which are ${\mathcalligra{f}}_{1} = \left\{ {\left( {\left[ {0.7,0.8} \right],\left[ {0.3,0.4} \right]} \right),\left( {\left[ {0.8,0.9} \right],\left[ {0.2,0.3} \right]} \right)} \right\}$ $i = 1$ and ${\mathcalligra{f}}_{2} = \left\{ {\left( {\left[ {0.5,0.8} \right],\left[ {0.3,0.5} \right]} \right),\left( {\left[ {0.7,0.9} \right],\left[ {0.2,0.4} \right]} \right),\left( {\left[ {0.8,0.9} \right],\left[ {0.2,0.5} \right]} \right)} \right\}$ $i = 2$ satisfying $0 < 0.8^{3} + 0.4^{3} \le 1$, $0 < 0.9^{3} + 0.3^{3} \le 1$, $0 < 0.8^{3} + 0.5^{3} \le 1$, $0 < 0.9^{3} + 0.4^{3} \le 1$, $0 < 0.9^{3} + 0.5^{3} \le 1$, separately.

According to the definition of IVFHFSs, there are some special circumstances here, apparently:

- if every $h_{{\mathcal{F}}} \left( s \right)$ just includes only one pair of intervals, i.e., $i = 1$, the IVFHFSs can be viewed as IVFFSs;
- if $\mu_{{\mathcal{F}}}^{ - } \left( s \right) = \mu_{{\mathcal{F}}}^{ + } \left( s \right)$ and $\nu_{{\mathcal{F}}}^{ - } \left( s \right) = \nu_{{\mathcal{F}}}^{ + } \left( s \right)$, the IVFHFSs reduce into FHFSs;
- if any interval-valued ND satisfies $\nu_{{\mathcal{F}}}^{ - } \left( s \right) = \nu_{{\mathcal{F}}}^{ + } \left( s \right) = 0$, then the IVFHFSs are considered to be IVHFSs.
- if all the $\mu_{{\mathcal{F}}}^{ + } \left( s \right)$ and $\nu_{{\mathcal{F}}}^{ + } \left( s \right)$ are constrained by the condition that $0 < (\mu_{{\mathcal{F}}}^{ + } )^{2} + (\nu_{{\mathcal{F}}}^{ + } )^{2} \le 1$, the IVFHFSs degrade into IVPHFSs.
- if all the $\mu_{{\mathcal{F}}}^{ + } \left( s \right)$ and $\nu_{{\mathcal{F}}}^{ + } \left( s \right)$ are constrained by the condition that $0 < \mu_{{\mathcal{F}}}^{ + } + \nu_{{\mathcal{F}}}^{ + } \left( x \right) \le 1$, the IVFHFSs degrade into IVIHFSs, similarly.

### Definition 2.5.

^22^ Let ${\mathcalligra{f}} = ([\mu_{i}^{ - } ,\mu_{i}^{ + } \left] {,[\nu_{i}^{ - } ,\nu_{i}^{ + } } \right])\left( {i = 1,2, \ldots ,k} \right)$ be an IVFHFN, then the SC of ${\mathcalligra{f}}$ is defined as follows:

2.1 $$\begin{array}{*{20}c} {SC\left( {{\mathcalligra{f}}} \right) = \left[ {\frac{1}{{2\left| {{\mathcalligra{f}}} \right|}}\mathop \sum \limits_{i = 1}^{{\left| {{\mathcalligra{f}}} \right|}} \left[ {(\mu_{i}^{ - } )^{3} - \left( {\nu_{i}^{ + } } \right)^{3} } \right],\frac{1}{{2\left| {{\mathcalligra{f}}} \right|}}\mathop \sum \limits_{i = 1}^{{\left| {{\mathcalligra{f}}} \right|}} \left[ {(\mu_{i}^{ + } )^{3} - \left( {\nu_{i}^{ - } } \right)^{3} } \right]} \right]} \\ \end{array}$$

Further, the following is the definition of the ${\mathcalligra{f}}$ accuracy function (AC):

2.2 $$\begin{array}{*{20}c} {AC\left( {{\mathcalligra{f}}} \right) = \left[ {\frac{1}{{2\left| {{\mathcalligra{f}}} \right|}}\mathop \sum \limits_{i = 1}^{{\left| {{\mathcalligra{f}}} \right|}} \left[ {(\mu_{i}^{ - } )^{3} + \left( {\nu_{i}^{ + } } \right)^{3} } \right],\frac{1}{{2\left| {{\mathcalligra{f}}} \right|}}\mathop \sum \limits_{i = 1}^{{\left| {{\mathcalligra{f}}} \right|}} \left[ {(\mu_{i}^{ + } )^{3} + \left( {\nu_{i}^{ - } } \right)^{3} } \right]} \right]} \\ \end{array}$$

As we can see, the SC and the AF are both interval numbers, and then we need to process them a step further.

### Definition 2.6.

^22^ Suppose that there are two interval numbers $A = \left[ {A^{ - } ,A^{ + } } \right]$ and $B = \left[ {B^{ - } ,B^{ + } } \right]$, and the likelihood of $A \geqslant B$ is stated as follows:

2.3 $$\begin{array}{*{20}c} {P\left( {A \geqslant B} \right) = \max \left\{ {1 - \max \left\{ {\frac{{B^{ + } - A^{ - } }}{J\left( A \right) + J\left( B \right)},0} \right\},0} \right\}} \\ \end{array}$$

where $J\left( A \right) = A^{ + } - A^{ - }$ and $J\left( B \right) = B^{ + } - B^{ - }$, and holds the following items:

- $$0 \le P\left( {A \geqslant B} \right) \le 1$$;
- $$if P\left( {A \geqslant B} \right) = P\left( {B \geqslant A} \right), P\left( {A \geqslant B} \right) = P\left( {B \geqslant A} \right) = 1/2$$;
- $$P\left( {A \geqslant B} \right) + P\left( {B \geqslant A} \right) = 1$$.

Using the above definition, we can obtain a precise value to compare the size of two IVFHFNs.

### Definition 2.7.

^22^ Let ${\mathcalligra{f}}_{1}$ and ${\mathcalligra{f}}_{2}$ be two IVFHFNs.

- If $$P\left( {SC({\mathcalligra{f}}_{1} } \right) \geqslant SC({\mathcalligra{f}}_{2} )) < \frac{1}{2}$$, then $${\mathcalligra{f}}_{1} \prec {\mathcalligra{f}}_{2}$$
- If $$P\left( {SC({\mathcalligra{f}}_{1} } \right) \geqslant SC({\mathcalligra{f}}_{2} )) = \frac{1}{2}$$, then
- If $P\left( {AC({\mathcalligra{f}}_{1} } \right) \geqslant AC({\mathcalligra{f}}_{2} )) < \frac{1}{2}$, we say ${\mathcalligra{f}}_{1} \prec {\mathcalligra{f}}_{2}$
- If $P\left( {AC({\mathcalligra{f}}_{1} } \right) \geqslant AC({\mathcalligra{f}}_{2} )) = \frac{1}{2}$, we say ${\mathcalligra{f}}_{1} = {\mathcalligra{f}}_{2}$

### Definition 2.8.

^45^ Let $E:\left[ {0,1} \right] \times \left[ {0,1} \right] \to \left[ {0,1} \right]$ be an Archimedean t-norm if it satisfies associativity, symmetricity, non-decreasing, and $E\left( {h,1} \right) = h$ for all $h$. It also caters to any $h \in \left( {0,1} \right)$ $H\left( {h,h} \right) < h$.

### Definition 2.9.

^45^ Let $K:\left[ {0,1} \right] \times \left[ {0,1} \right] \to \left[ {0,1} \right]$ be an Archimedean t-conorm if it satisfies associativity, symmetricity, non-decreasing, and $K\left( {h,0} \right) = h$ for all $h$. It also caters to any $h \in \left( {0,1} \right)$ $K\left( {h,h} \right) > h$.

### Definition 2.10.

^32^ Let $\sigma ,\tau \ge 0$, and $a_{x} \left( {x = 1,2, \ldots ,n} \right)$ be a non-empty and non-negative set. The BM is defined as:

2.4 $$\begin{array}{*{20}c} {BM^{\sigma ,\tau } \left( {a_{1} ,a_{2} , \ldots ,a_{n} } \right) = \left( {\frac{1}{{n\left( {n - 1} \right)}}\mathop \sum \limits_{x,y = 1;x \ne y}^{n} a_{x}^{\sigma } a_{y}^{\tau } } \right)^{{\frac{1}{\sigma + \tau }}} } \\ \end{array}$$

## The IVFHFEBM and IVFHFEWBM AOs under E-TNs

In this section, we propose the E-TNs operation on IVFHFNs. We investigate the extension AOs of BM based on the E-TNs operations under the IVFHF environment. Considering the effect of attribute weights, we also propose the AO of IVFHFEWBM.

### The Einstein operations on IVFHFNs

Einstein operations are basic operations based on the AR-TNs derived by substituting functions that satisfy the relevant conditions. Firstly, we propose the arithmetic operations based on AR-TNs under the IVFHFSs environment.

#### Definition 3.1.

Let ${\mathcalligra{f}} = ([\mu_{i}^{ - } ,\mu_{i}^{ + } \left] {,[\nu_{i}^{ - } ,\nu_{i}^{ + } } \right])$, ${\mathcalligra{f}}_{1} = ([\mu_{1i}^{ - } ,\mu_{1i}^{ + } \left] {,[\nu_{1i}^{ - } ,\nu_{1i}^{ + } } \right])$ and ${\mathcalligra{f}}_{2} = ([\mu_{2i}^{ - } ,\mu_{2i}^{ + } \left] {,[\nu_{2i}^{ - } ,\nu_{2i}^{ + } } \right])$ $\left( {i = 1,2, \ldots ,k} \right)$ be three IVFHFNs, where $i$ denotes the number of hesitation elements. The arithmetic operations based on AR-TNs in the IVFHFSs environment are expressed as:

1. $${\mathcalligra{f}}^{\lambda } = \mathop {{\mathcalligra{f}}}\limits_{{([\mu_{i}^{ - } ,\mu_{i}^{ + } \left] {,[\nu_{i}^{ - } ,\nu_{i}^{ + } } \right]) \in {\mathcalligra{f}}}} \left( {\begin{array}{*{20}c} {\left[ {\sqrt[3]{{\dot{\varepsilon }^{ - 1} \left( {\lambda \dot{\varepsilon }\left( {\left( {\mu_{i}^{ - } } \right)^{3} } \right)} \right)}},\sqrt[3]{{\dot{\varepsilon }^{ - 1} \left( {\lambda \dot{\varepsilon }\left( {\left( {\mu_{i}^{ + } } \right)^{3} } \right)} \right)}}} \right],} \\ {\left[ {\sqrt[3]{{\dot{s}^{ - 1} \left( {\lambda \dot{s}\left( {\left( {\nu_{i}^{ - } } \right)^{3} } \right)} \right)}},\sqrt[3]{{\dot{s}^{ - 1} \left( {\lambda \dot{s}\left( {\left( {\nu_{i}^{ + } } \right)^{3} } \right)} \right)}}} \right]} \\ \end{array} } \right)$$
2. $$\lambda {\mathcalligra{f}} = \mathop {{\mathcalligra{f}}}\limits_{{([\mu_{i}^{ - } ,\mu_{i}^{ + } \left] {,[\nu_{i}^{ - } ,\nu_{i}^{ + } } \right]) \in {\mathcalligra{f}}}} \left( {\begin{array}{*{20}c} {\left[ {\sqrt[3]{{\dot{s}^{ - 1} \left( {\lambda \dot{s}\left( {\left( {\mu_{i}^{ - } } \right)^{3} } \right)} \right)}},\sqrt[3]{{\dot{s}^{ - 1} \left( {\lambda \dot{s}\left( {\left( {\mu_{i}^{ + } } \right)^{3} } \right)} \right)}}} \right],} \\ {\left[ {\sqrt[3]{{\varepsilon^{ - 1} \left( {\lambda \dot{\varepsilon }\left( {\left( {\nu_{i}^{ - } } \right)^{3} } \right)} \right)}},\sqrt[3]{{\dot{\varepsilon }^{ - 1} \left( {\lambda \dot{\varepsilon }\left( {\left( {\nu_{i}^{ + } } \right)^{3} } \right)} \right)}}} \right]} \\ \end{array} } \right)$$
3. $${\mathcalligra{f}}_{1} \oplus {\mathcalligra{f}}_{2} = \bigcup\limits_{{\begin{array}{*{20}c} {([\mu_{1i}^{ - } ,\mu_{i}^{ + } \left] {,[\nu_{1i}^{ - } ,\nu_{1i}^{ + } } \right]) \in {\mathcalligra{f}}_{1} ,} \\ {([\mu_{2i}^{ - } ,\mu_{2i}^{ + } \left] {,[\nu_{2i}^{ - } ,\nu_{2i}^{ + } } \right]) \in {\mathcalligra{f}}_{2} } \\ \end{array} }} {\left( {\begin{array}{*{20}l} {\left[ {\sqrt[3]{{K\left( {\left( {\mu_{1i}^{ - } } \right)^{3} ,\left( {\mu_{2i}^{ - } } \right)^{3} } \right)}},\sqrt[3]{{K\left( {\left( {\mu_{1i}^{ + } } \right)^{3} ,\left( {\mu_{2i}^{ + } } \right)^{3} } \right)}}} \right],} \hfill \\ {\left[ {\sqrt[3]{{E\left( {\left( {\nu_{1i}^{ - } } \right)^{3} ,\left( {\nu_{2i}^{ - } } \right)^{3} } \right)}},\sqrt[3]{{E\left( {\left( {\nu_{1i}^{ + } } \right)^{3} ,\left( {\nu_{2i}^{ + } } \right)^{3} } \right)}}} \right]} \hfill \\ \end{array} } \right)}$$
4. $${\mathcalligra{f}}_{1} \otimes {\mathcalligra{f}}_{2} = \bigcup\limits_{{\begin{array}{*{20}c} {([\mu_{1i}^{ - } ,\mu_{1i}^{ + } \left] {,[\nu_{1i}^{ - } ,\nu_{1i}^{ + } } \right]) \in {\mathcalligra{f}}_{1} ,} \\ {([\mu_{2i}^{ - } ,\mu_{2i}^{ + } \left] {,[\nu_{2i}^{ - } ,\nu_{2i}^{ + } } \right]) \in {\mathcalligra{f}}_{2} } \\ \end{array} }} {\left( {\begin{array}{*{20}l} {\left[ {\sqrt[3]{{E\left( {\left( {\mu_{1i}^{ - } } \right)^{3} ,\left( {\mu_{2i}^{ - } } \right)^{3} } \right)}},\sqrt[3]{{E\left( {\left( {\mu_{1i}^{ + } } \right)^{3} ,\left( {\mu_{2i}^{ + } } \right)^{3} } \right)}}} \right],} \hfill \\ {\left[ {\sqrt[3]{{K\left( {\left( {\nu_{1i}^{ - } } \right)^{3} ,\left( {\nu_{2i}^{ - } } \right)^{3} } \right)}},\sqrt[3]{{K\left( {\left( {\nu_{1i}^{ + } } \right)^{3} ,\left( {\nu_{2i}^{ + } } \right)^{3} } \right)}}} \right]} \hfill \\ \end{array} } \right)}$$

where the function $\dot{\varepsilon }$ is called an additive generating element expressing that Archimedean t-norm as $E\left( {h,z} \right) = \dot{\varepsilon }^{ - 1} \left( {\dot{\tau }\left( h \right) + \dot{\varepsilon }\left( z \right)} \right)$, and the function $\dot{s}\left( t \right) = \dot{\varepsilon }\left( {1 - t} \right)$ represents that Archimedean t-conorm as $K\left( {h,z} \right) = \dot{s}^{ - 1} \left( {\dot{s}\left( h \right) + \dot{s}\left( z \right)} \right)$.

Then, according to Definition 3.1, we simply bring in the corresponding E-TNs to obtain Einstein operations on IVFHFNs.

In the E-TNs setting^25,26^, we have $\dot{\varepsilon }\left( t \right) = log_{2} \left( {\frac{2 - t}{t}} \right)$, $\dot{s}\left( t \right) = log_{2} \left( {\frac{1 + t}{{1 - t}}} \right)$, and by means of $\dot{\varepsilon }\left( t \right)$ and $\dot{s}\left( t \right)$ we can deduce that $E\left( {h,z} \right) = \frac{hz}{{1 + \left( {1 - h} \right)\left( {1 - z} \right)}}$ and $K\left( {h,z} \right) = \frac{h + z}{{1 + hz}}$ and $\varepsilon^{ - 1} \left( t \right) = \frac{2}{{2^{t} + 1}}$, $\dot{s}^{ - 1} \left( t \right) = \frac{{2^{t} - 1}}{{2^{t} + 1}}$, respectively.

So, we can obtain Definition 3.2 as follows:

#### Definition 3.2.

Suppose that there are three IVFHFNs: ${\mathcalligra{f}} = ([\mu_{i}^{ - } ,\mu_{i}^{ + } \left] {,[\nu_{i}^{ - } ,\nu_{i}^{ + } } \right])$, ${\mathcalligra{f}}_{1} = ([\mu_{1i}^{ - } ,\mu_{1i}^{ + } \left] {,[\nu_{1i}^{ - } ,\nu_{1i}^{ + } } \right])$ and ${\mathcalligra{f}}_{2} = ([\mu_{2i}^{ - } ,\mu_{2i}^{ + } \left] {,[\nu_{2i}^{ - } ,\nu_{2i}^{ + } } \right])\left( {i = 1,2, \ldots ,k} \right)$, separately. The operations based on E-TNs under the IVFHFSs environment are expressed as:

1. $${\mathcalligra{f}}^{\lambda } = \bigcup\limits_{{([\mu_{i}^{ - } ,\mu_{i}^{ + } \left] {,[\nu_{i}^{ - } ,\nu_{i}^{ + } } \right]) \in {\mathcalligra{f}}}} {\left( {\begin{array}{*{20}l} {\left[ {\sqrt[3]{{\frac{{2\left[ {\left( {\mu_{i}^{ - } } \right)^{3} } \right]^{\lambda } }}{{\left[ {2 - \left( {\mu_{i}^{ - } } \right)^{3} } \right]^{\lambda } + \left[ {\left( {\mu_{i}^{ - } } \right)^{3} } \right]^{\lambda } }}}},\sqrt[3]{{\frac{{2\left[ {\left( {\mu_{i}^{ + } } \right)^{3} } \right]^{\lambda } }}{{\left[ {2 - \left( {\mu_{i}^{ + } } \right)^{3} } \right]^{\lambda } + \left[ {\left( {\mu_{i}^{ + } } \right)^{3} } \right]^{\lambda } }}}}} \right],} \hfill \\ {\left[ {\sqrt[3]{{\frac{{\left[ {1 + \left( {\nu_{i}^{ - } } \right)^{3} } \right]^{\lambda } - \left[ {1 - \left( {\nu_{i}^{ - } } \right)^{3} } \right]^{\lambda } }}{{\left[ {1 + \left( {\nu_{i}^{ - } } \right)^{3} } \right]^{\lambda } + \left[ {1 - \left( {\nu_{i}^{ - } } \right)^{3} } \right]^{\lambda } }}}},\sqrt[3]{{\frac{{\left[ {1 + \left( {\nu_{i}^{ + } } \right)^{3} } \right]^{\lambda } - \left[ {1 - \left( {\nu_{i}^{ + } } \right)^{3} } \right]^{\lambda } }}{{\left[ {1 + \left( {\nu_{i}^{ + } } \right)^{3} } \right]^{\lambda } + \left[ {1 - \left( {\nu_{i}^{ + } } \right)^{3} } \right]^{\lambda } }}}}} \right]} \hfill \\ \end{array} } \right)}$$
2. $$\lambda {\mathcalligra{f}} = \bigcup\limits_{{([\mu_{i}^{ - } ,\mu_{i}^{ + } \left] {,[\nu_{i}^{ - } ,\nu_{i}^{ + } } \right]) \in {\mathcalligra{f}}}} {\left( {\begin{array}{*{20}l} {\left[ {\sqrt[3]{{\frac{{\left[ {1 + \left( {\mu_{i}^{ - } } \right)^{3} } \right]^{\lambda } - \left[ {1 - \left( {\mu_{i}^{ - } } \right)^{3} } \right]^{\lambda } }}{{\left[ {1 + \left( {\mu_{i}^{ - } } \right)^{3} } \right]^{\lambda } + \left[ {1 - \left( {\mu_{i}^{ - } } \right)^{3} } \right]^{\lambda } }}}},\sqrt[3]{{\frac{{\left[ {1 + \left( {\mu_{i}^{ + } } \right)^{3} } \right]^{\lambda } - \left[ {1 - \left( {\mu_{i}^{ + } } \right)^{3} } \right]^{\lambda } }}{{\left[ {1 + \left( {\mu_{i}^{ + } } \right)^{3} } \right]^{\lambda } + \left[ {1 - \left( {\mu_{i}^{ + } } \right)^{3} } \right]^{\lambda } }}}}} \right],} \hfill \\ {\left[ {\sqrt[3]{{\frac{{2\left[ {\left( {\nu_{i}^{ - } } \right)^{3} } \right]^{\lambda } }}{{\left[ {2 - \left( {\nu_{i}^{ - } } \right)^{3} } \right]^{\lambda } + \left[ {\left( {\nu_{i}^{ - } } \right)^{3} } \right]^{\lambda } }}}},\sqrt[3]{{\frac{{2\left[ {\left( {\nu_{i}^{ + } } \right)^{3} } \right]^{\lambda } }}{{\left[ {2 - \left( {\nu_{i}^{ + } } \right)^{3} } \right]^{\lambda } + \left[ {\left( {\nu_{i}^{ + } } \right)^{3} } \right]^{\lambda } }}}}} \right]} \hfill \\ \end{array} } \right)}$$
3. $${\mathcalligra{f}}_{1} \oplus {\mathcalligra{f}}_{2} = \bigcup\limits_{{\begin{array}{*{20}c} {([\mu_{1i}^{ - } ,\mu_{1i}^{ + } \left] {,[\nu_{1i}^{ - } ,\nu_{1i}^{ + } } \right]) \in {\mathcalligra{f}}_{1} ,} \\ {([\mu_{2i}^{ - } ,\mu_{2i}^{ + } \left] {,[\nu_{2i}^{ - } ,\nu_{2i}^{ + } } \right]) \in {\mathcalligra{f}}_{2} } \\ \end{array} }} {\left( {\begin{array}{*{20}l} {\left[ {\sqrt[3]{{\frac{{\left( {\mu_{1i}^{ - } } \right)^{3} + \left( {\mu_{2i}^{ - } } \right)^{3} }}{{1 + \left( {\mu_{1i}^{ - } } \right)^{3} \left( {\mu_{2i}^{ - } } \right)^{3} }}}},\sqrt[3]{{\frac{{\left( {\mu_{1i}^{ + } } \right)^{3} + \left( {\mu_{2i}^{ + } } \right)^{3} }}{{1 + \left( {\mu_{1i}^{ + } } \right)^{3} \left( {\mu_{2i}^{ + } } \right)^{3} }}}}} \right],} \hfill \\ {\left[ {\frac{{\nu_{1i}^{ - } \nu_{2i}^{ - } }}{{\sqrt[3]{{1 + \left[ {1 - \left( {\nu_{1i}^{ - } } \right)^{3} } \right]\left[ {1 - \left( {\nu_{2i}^{ - } } \right)^{3} } \right]}}}},\frac{{\nu_{1i}^{ + } \nu_{2i}^{ + } }}{{\sqrt[3]{{1 + \left[ {1 - \left( {\nu_{1i}^{ + } } \right)^{3} } \right]\left[ {1 - \left( {\nu_{2i}^{ + } } \right)^{3} } \right]}}}}} \right]} \hfill \\ \end{array} } \right)}$$
4. $${\mathcalligra{f}}_{1} \otimes {\mathcalligra{f}}_{2} = \bigcup\limits_{{\begin{array}{*{20}c} {([\mu_{1i}^{ - } ,\mu_{1i}^{ + } \left] {,[\nu_{1i}^{ - } ,\nu_{1i}^{ + } } \right]) \in {\mathcalligra{f}}_{1} ,} \\ {([\mu_{2i}^{ - } ,\mu_{2i}^{ + } \left] {,[\nu_{2i}^{ - } ,\nu_{2i}^{ + } } \right]) \in {\mathcalligra{f}}_{2} } \\ \end{array} }} {\left( {\begin{array}{*{20}l} {\left[ {\frac{{\mu_{1i}^{ - } \mu_{2i}^{ - } }}{{\sqrt[3]{{1 + \left[ {1 - \left( {\mu_{1i}^{ - } } \right)^{3} } \right]\left[ {1 - \left( {\mu_{2i}^{ - } } \right)^{3} } \right]}}}},\frac{{\mu_{1i}^{ + } \mu_{2i}^{ + } }}{{\sqrt[3]{{1 + \left[ {1 - \left( {\mu_{1i}^{ + } } \right)^{3} } \right]\left[ {1 - \left( {\mu_{2i}^{ + } } \right)^{3} } \right]}}}}} \right],} \hfill \\ {\left[ {\sqrt[3]{{\frac{{\left( {\nu_{1i}^{ - } } \right)^{3} + \left( {\nu_{2i}^{ - } } \right)^{3} }}{{1 + \left( {\nu_{1i}^{ - } } \right)^{3} \left( {\nu_{2i}^{ - } } \right)^{3} }}}},\sqrt[3]{{\frac{{\left( {\nu_{1i}^{ + } } \right)^{3} + \left( {\nu_{2i}^{ + } } \right)^{3} }}{{1 + \left( {\nu_{1i}^{ + } } \right)^{3} \left( {\nu_{2i}^{ + } } \right)^{3} }}}}} \right]} \hfill \\ \end{array} } \right)}$$
5. $${\mathcalligra{f}}^{C} = \mathop {{\mathcalligra{f}}}\limits_{{([\mu_{i}^{ - } ,\mu_{i}^{ + } \left] {,[\nu_{i}^{ - } ,\nu_{i}^{ + } } \right]) \in {\mathcalligra{f}}}} \left( {[\nu_{i}^{ - } ,\nu_{i}^{ + } } \right],[\mu_{i}^{ - } ,\mu_{i}^{ + } ])$$

#### Theorem 3.1.

Let ${\mathcalligra{f}} = ([\mu_{i}^{ - } ,\mu_{i}^{ + } \left] {,[\nu_{i}^{ - } ,\nu_{i}^{ + } } \right])$, ${\mathcalligra{f}}_{1} = ([\mu_{1i}^{ - } ,\mu_{1i}^{ + } \left] {,[\nu_{1i}^{ - } ,\nu_{1i}^{ + } } \right])$ and ${\mathcalligra{f}}_{2} = ([\mu_{2i}^{ - } ,\mu_{2i}^{ + } \left] {,[\nu_{2i}^{ - } ,\nu_{2i}^{ + } } \right])\left( {i = 1,2, \ldots ,k} \right)$ be three IVFHFNs, $\lambda > 0$ and both of ${\mathcalligra{f}}^{C}$, ${\mathcalligra{f}}^{\lambda }$, $\lambda {\mathcalligra{f}}$, ${\mathcalligra{f}}_{1} \oplus {\mathcalligra{f}}_{2}$ and ${\mathcalligra{f}}_{1} \otimes {\mathcalligra{f}}_{2}$ are IVFHNs.

#### Proof.

IVFHNs satisfy that for any $([\mu_{i}^{ - } ,\mu_{i}^{ + } \left] {,[\nu_{i}^{ - } ,\nu_{i}^{ + } } \right]) \in {\mathcalligra{f}}$ we have $0 < (\mu_{i}^{ + } )^{3} + (\nu_{i}^{ + } )^{3} \le 1$.

Hence, it is a very simple matter to prove which ${\mathcalligra{f}}^{C}$ is an IVFHN, and we omitted it.

When $\lambda > 0$, we can deduce:

$$\begin{aligned} & \frac{{2\left[ {\left( {\mu_{i}^{ + } } \right)^{3} } \right]^{\lambda } }}{{\left[ {2 - \left( {\mu_{i}^{ + } } \right)^{3} } \right]^{\lambda } + \left[ {\left( {\mu_{i}^{ + } } \right)^{3} } \right]^{\lambda } }} + \frac{{\left[ {1 + \left( {\nu_{i}^{ + } } \right)^{3} } \right]^{\lambda } - \left[ {1 - \left( {\nu_{i}^{ + } } \right)^{3} } \right]^{\lambda } }}{{\left[ {1 + \left( {\nu_{i}^{ + } } \right)^{3} } \right]^{\lambda } + \left[ {1 - \left( {\nu_{i}^{ + } } \right)^{3} } \right]^{\lambda } }} \\ & \quad \le \frac{{2\left[ {1 - \left( {\nu_{i}^{ + } } \right)^{3} } \right]^{\lambda } }}{{\left[ {1 + \left( {\nu_{i}^{ + } } \right)^{3} } \right]^{\lambda } + \left[ {1 - \left( {\nu_{i}^{ + } } \right)^{3} } \right]^{\lambda } }} + \frac{{\left[ {1 + \left( {\nu_{i}^{ + } } \right)^{3} } \right]^{\lambda } - \left[ {1 - \left( {\nu_{i}^{ + } } \right)^{3} } \right]^{\lambda } }}{{\left[ {1 + \left( {\nu_{i}^{ + } } \right)^{3} } \right]^{\lambda } + \left[ {1 - \left( {\nu_{i}^{ + } } \right)^{3} } \right]^{\lambda } }} = 1 \\ \end{aligned}$$

Thus, ${\mathcalligra{f}}^{\lambda }$ is an IVFHN. $\lambda {\mathcalligra{f}}$ is also an IVFHN, similarly. As for ${\mathcalligra{f}}_{1} \oplus {\mathcalligra{f}}_{2}$,

$$\begin{aligned} & \frac{{\left( {\mu_{1i}^{ + } } \right)^{3} + \left( {\mu_{2i}^{ + } } \right)^{3} }}{{1 + \left( {\mu_{1i}^{ + } } \right)^{3} \left( {\mu_{2i}^{ + } } \right)^{3} }} + \frac{{(\nu_{1i}^{ + } \nu_{2i}^{ + } )^{3} }}{{1 + \left[ {1 - \left( {\nu_{1i}^{ + } } \right)^{3} } \right]\left[ {1 - \left( {\nu_{2i}^{ + } } \right)^{3} } \right]}} \\ & \quad \le \frac{{\left[ {1 - \left( {\nu_{1i}^{ + } } \right)^{3} } \right] + \left[ {1 - \left( {\nu_{2i}^{ + } } \right)^{3} } \right]}}{{1 + \left[ {1 - \left( {\nu_{1i}^{ + } } \right)^{3} } \right]\left[ {1 - \left( {\nu_{2i}^{ + } } \right)^{3} } \right]}} + \frac{{(\nu_{1i}^{ + } \nu_{2i}^{ + } )^{3} }}{{1 + \left[ {1 - \left( {\nu_{1i}^{ + } } \right)^{3} } \right]\left[ {1 - \left( {\nu_{2i}^{ + } } \right)^{3} } \right]}} = 1 \\ \end{aligned}$$

Thus, ${\mathcalligra{f}}_{1} \oplus {\mathcalligra{f}}_{2}$ is an IVFHN. ${\mathcalligra{f}}_{1} \otimes {\mathcalligra{f}}_{2}$ is also an IVFHN, similarly.

#### Theorem 3.2.

Let ${\mathcalligra{f}} = ([\mu_{i}^{ - } ,\mu_{i}^{ + } \left] {,[\nu_{i}^{ - } ,\nu_{i}^{ + } } \right])$, ${\mathcalligra{f}}_{1} = ([\mu_{1i}^{ - } ,\mu_{1i}^{ + } \left] {,[\nu_{1i}^{ - } ,\nu_{1i}^{ + } } \right])$ and ${\mathcalligra{f}}_{2} = ([\mu_{2i}^{ - } ,\mu_{2i}^{ + } \left] {,[\nu_{2i}^{ - } ,\nu_{2i}^{ + } } \right])\left( {i = 1,2, \ldots ,k} \right)$ be three IVFHFNs, and $\lambda_{1} , \lambda_{2} ,\lambda > 0$. Then we have:

1. $${\mathcalligra{f}}_{1} \oplus {\mathcalligra{f}}_{2} = {\mathcalligra{f}}_{2} \oplus {\mathcalligra{f}}_{1}$$;
2. $${\mathcalligra{f}}_{1} \otimes {\mathcalligra{f}}_{2} = {\mathcalligra{f}}_{2} \otimes {\mathcalligra{f}}_{1}$$;
3. $$\lambda \left( {{\mathcalligra{f}}_{1} \oplus {\mathcalligra{f}}_{2} } \right) = \lambda {\mathcalligra{f}}_{1} \oplus \lambda {\mathcalligra{f}}_{2}$$;
4. $$\lambda_{1} {\mathcalligra{f}} \oplus \lambda_{2} {\mathcalligra{f}} = \left( {\lambda_{1} + \lambda_{2} } \right){\mathcalligra{f}}$$;
5. $$\left( {{\mathcalligra{f}}_{1} \otimes {\mathcalligra{f}}_{2} } \right)^{\lambda } = {\mathcalligra{f}}_{1}^{\lambda } \otimes {\mathcalligra{f}}_{2}^{\lambda }$$;
6. $${\mathcalligra{f}}^{{\lambda_{1} }} \otimes {\mathcalligra{f}}^{{\lambda_{2} }} = {\mathcalligra{f}}^{{\lambda_{1} + \lambda_{2} }}$$.

#### Proof.

We can easily derive Theorem 3.2 from Definition 3.2, so we omitted this part of the proof step.

#### Proposition 3.1.

Let ${\mathcalligra{f}}_{1} = ([\mu_{1i}^{ - } ,\mu_{1i}^{ + } \left] {,[\nu_{1i}^{ - } ,\nu_{1i}^{ + } } \right])$ and ${\mathcalligra{f}}_{2} = ([\mu_{2i}^{ - } ,\mu_{2i}^{ + } \left] {,[\nu_{2i}^{ - } ,\nu_{2i}^{ + } } \right])\left( {i = 1,2, \ldots ,k} \right)$ be two IVFHFNs. If the number of $i$ where belongs to ${\mathcalligra{f}}_{1}$ equals the number of $i$ where belongs to ${\mathcalligra{f}}_{2}$ and $\mu_{1i}^{ - } = \mu_{2i}^{ - }$, $\mu_{1i}^{ + } = \mu_{2i}^{ + }$, $\nu_{1i}^{ - } = \nu_{2i}^{ - }$, $\nu_{1i}^{ + } = \nu_{2i}^{ + }$, respectively, then we can call ${\mathcalligra{f}}_{1} = {\mathcalligra{f}}_{2}$.

### The AO of IVFHFEBM

We can see that the BM operator in Definition 2.10 considers the interrelationships between input parameters. It relates the input parameters themselves to all the other remaining parameters. By applying the input parameters of the initial BM operator to the IVFHFNs, we obtain an IVFHFEBM that can be adapted to the IVFHF environment. Based on Definitions 2.10 and 3.2, the AO of IVFHFBM can be expressed as follows:

#### Definition 3.3.

Let ${\mathcalligra{f}}_{x} = ([\mu_{xi}^{ - } ,\mu_{xi}^{ + } \left] {,[\nu_{xi}^{ - } ,\nu_{xi}^{ + } } \right])\left( {x = 1,2, \ldots ,n,i = 1,2, \ldots ,k} \right)$ consists of a group of IVFHFNs and the IVFHFEBM is defined as:

3.1 $$\begin{array}{*{20}c} {IVFHFEBM^{\sigma ,\tau } \left( {{\mathcalligra{f}}_{1} ,{\mathcalligra{f}}_{2} , \ldots ,{\mathcalligra{f}}_{n} } \right) = \left\{ {\frac{1}{{n\left( {n - 1} \right)}}\left[ {\begin{array}{*{20}c} n \\ \oplus \\ {x,y = 1;x \ne y} \\ \end{array} \left( {{\mathcalligra{f}}_{x}^{\sigma } \otimes {\mathcalligra{f}}_{y}^{\tau } } \right)} \right]} \right\}^{{\frac{1}{\sigma + \tau }}} } \\ \end{array}$$

where the parameters $\sigma ,\tau > 0$ and $n > 1$.

According to the E-TNs operational laws of the IVFHFNs and Definition 3.3, the following propositions can be acquired:

#### Lemma 3.1.

Assume that ${\mathcalligra{f}}_{x} = ([\mu_{xi}^{ - } ,\mu_{xi}^{ + } \left] {,[\nu_{xi}^{ - } ,\nu_{xi}^{ + } } \right])\left( {x = 1,2, \ldots ,n,i = 1,2, \ldots ,k} \right)$ consists of a group of IVFHFNs, and $\sigma ,\tau \ge 0$. Then,

3.2 $${\mathcalligra{f}}_{x}^{\sigma } \otimes {\mathcalligra{f}}_{y}^{\tau } = \bigcup\limits_{{([\mu_{i}^{ - } ,\mu_{i}^{ + } \left] {,[\nu_{i}^{ - } ,\nu_{i}^{ + } } \right]) \in {\mathcalligra{f}}}} {\left( {\begin{array}{*{20}l} {\left[ {\begin{array}{*{20}c} {\frac{{\sqrt[3]{{2\left[ {\left( {\mu_{xi}^{ - } } \right)^{3} } \right]^{\sigma } \left[ {\left( {\mu_{yi}^{ - } } \right)^{3} } \right]^{\tau } }}}}{{\sqrt[3]{{\left[ {2 - \left( {\mu_{xi}^{ - } } \right)^{3} } \right]^{\sigma } \left[ {2 - \left( {\mu_{yi}^{ - } } \right)^{3} } \right]^{\tau } + \left[ {\left( {\mu_{xi}^{ - } } \right)^{3} } \right]^{\sigma } \left[ {\left( {\mu_{yi}^{ - } } \right)^{3} } \right]^{\tau } }}}},} \\ {\frac{{\sqrt[3]{{2\left[ {\left( {\mu_{xi}^{ + } } \right)^{3} } \right]^{\sigma } \left[ {\left( {\mu_{yi}^{ + } } \right)^{3} } \right]^{\tau } }}}}{{\sqrt[3]{{\left[ {2 - \left( {\mu_{xi}^{ + } } \right)^{3} } \right]^{\sigma } \left[ {2 - \left( {\mu_{yi}^{ + } } \right)^{3} } \right]^{\tau } + \left[ {\left( {\mu_{xi}^{ + } } \right)^{3} } \right]^{\sigma } \left[ {\left( {\mu_{yi}^{ + } } \right)^{3} } \right]^{\tau } }}}}} \\ \end{array} } \right],} \hfill \\ {\left[ {\begin{array}{*{20}l} {\sqrt[3]{{\frac{{[1 + \left( {\nu_{xi}^{ - } } \right)^{3} ]^{\sigma } \left[ {1 + \left( {\nu_{yi}^{ - } } \right)^{3} } \right]^{\tau } - \left[ {1 - \left( {\nu_{xi}^{ - } } \right)^{3} } \right]^{\sigma } \left[ {1 - \left( {\nu_{yi}^{ - } } \right)^{3} } \right]^{\tau } }}{{[1 + \left( {\nu_{xi}^{ - } } \right)^{3} ]^{\sigma } \left[ {1 + \left( {\nu_{yi}^{ - } } \right)^{3} } \right]^{\tau } + \left[ {1 - \left( {\nu_{xi}^{ - } } \right)^{3} } \right]^{\sigma } \left[ {1 - \left( {\nu_{yi}^{ - } } \right)^{3} } \right]^{\tau } }}}},} \hfill \\ {\sqrt[3]{{\frac{{\left[ {1 + \left( {\nu_{xi}^{ + } } \right)^{3} } \right]^{\sigma } \left[ {1 + \left( {\nu_{yi}^{ + } } \right)^{3} } \right]^{\tau } - \left[ {1 - \left( {\nu_{xi}^{ + } } \right)^{3} } \right]^{\sigma } \left[ {1 - \left( {\nu_{yi}^{ + } } \right)^{3} } \right]^{\tau } }}{{\left[ {1 + \left( {\nu_{xi}^{ + } } \right)^{3} } \right]^{\sigma } \left[ {1 + \left( {\nu_{yi}^{ + } } \right)^{3} } \right]^{\tau } + \left[ {1 - \left( {\nu_{xi}^{ + } } \right)^{3} } \right]^{\sigma } \left[ {1 - \left( {\nu_{yi}^{ + } } \right)^{3} } \right]^{\tau } }}}}} \hfill \\ \end{array} } \right]} \hfill \\ \end{array} } \right)}$$

#### Proof.

Firstly, To make the proof process more concise, we let $U_{xi}^{ - } = 2 - \left( {\mu_{xi}^{ - } } \right)^{3}$, $U_{yi}^{ - } = 2 - \left( {\mu_{yi}^{ - } } \right)^{3}$, $VA_{xi}^{ - } = 1 + \left( {\nu_{xi}^{ - } } \right)^{3}$, $VA_{yi}^{ - } = 1 + \left( {\nu_{yi}^{ - } } \right)^{3}$, $VS_{xi}^{ - } = 1 - \left( {\nu_{xi}^{ - } } \right)^{3}$, $VS_{yi}^{ - } = 1 - \left( {\nu_{yi}^{ - } } \right)^{3}$ and $U_{xi}^{ + }$, $U_{yi}^{ + }$, $VA_{xi}^{ + }$, $VA_{yi}^{ + }$, $VS_{xi}^{ + }$, $VS_{yi}^{ + }$ are to replace $-$ with $+$ in above formula. According to Einstein's basic operations in Definition 3.2, we have

$$\begin{aligned} & {\mathcalligra{f}}_{x}^{\sigma } \\ & \quad = \bigcup\limits_{{([\mu_{i}^{ - } ,\mu_{i}^{ + } \left] {,[\nu_{i}^{ - } ,\nu_{i}^{ + } } \right]) \in {\mathcalligra{f}}}} {\left( {\left[ {\begin{array}{*{20}c} {\sqrt[3]{{\frac{{2\left[ {\left( {\mu_{xi}^{ - } } \right)^{3} } \right]^{\sigma } }}{{\left[ {U_{xi}^{ - } } \right]^{\sigma } + \left[ {\left( {\mu_{xi}^{ - } } \right)^{3} } \right]^{\sigma } }}}},\sqrt[3]{{\frac{{2\left[ {\left( {\mu_{xi}^{ + } } \right)^{3} } \right]^{\sigma } }}{{\left[ {U_{xi}^{ + } } \right]^{\sigma } + \left[ {\left( {\mu_{xi}^{ + } } \right)^{3} } \right]^{\sigma } }}}}} \\ \end{array} } \right],\left[ {\begin{array}{*{20}c} {\sqrt[3]{{\frac{{\left[ {VA_{xi}^{ - } } \right]^{\sigma } - \left[ {VS_{xi}^{ - } } \right]^{\sigma } }}{{\left[ {VA_{xi}^{ - } } \right]^{\sigma } + \left[ {VS_{xi}^{ - } } \right]^{\sigma } }}}},\sqrt[3]{{\frac{{\left[ {VS_{xi}^{ + } } \right]^{\sigma } - \left[ {VS_{xi}^{ + } } \right]^{\sigma } }}{{\left[ {VS_{xi}^{ + } } \right]^{\sigma } + \left[ {VS_{xi}^{ + } } \right]^{\sigma } }}}}} \\ \end{array} } \right]} \right)} \\ & {\mathcalligra{f}}_{y}^{\tau } \\ & \quad = \bigcup\limits_{{([\mu_{i}^{ - } ,\mu_{i}^{ + } \left] {,[\nu_{i}^{ - } ,\nu_{i}^{ + } } \right]) \in {\mathcalligra{f}}}} {\left( {\left[ {\begin{array}{*{20}c} {\sqrt[3]{{\frac{{2\left[ {\left( {\mu_{yi}^{ - } } \right)^{3} } \right]^{\tau } }}{{\left[ {U_{yi}^{ - } } \right]^{\tau } + \left[ {\left( {\mu_{yi}^{ - } } \right)^{3} } \right]^{\tau } }}}},} \\ \end{array} \sqrt[3]{{\frac{{2\left[ {\left( {\mu_{yi}^{ + } } \right)^{3} } \right]^{\tau } }}{{\left[ {U_{yi}^{ + } } \right]^{\tau } + \left[ {\left( {\mu_{yi}^{ + } } \right)^{3} } \right]^{\tau } }}}}} \right],\left[ {\begin{array}{*{20}c} {\sqrt[3]{{\frac{{\left[ {VA_{yi}^{ - } } \right]^{\tau } - \left[ {VS_{yi}^{ - } } \right]^{\tau } }}{{\left[ {VA_{yi}^{ - } } \right]^{\tau } + \left[ {VS_{yi}^{ - } } \right]^{\tau } }}}},\sqrt[3]{{\frac{{\left[ {VA_{yi}^{ + } } \right]^{\tau } - \left[ {VS_{yi}^{ + } } \right]^{\tau } }}{{\left[ {VA_{yi}^{ + } } \right]^{\tau } + \left[ {VS_{yi}^{ + } } \right]^{\tau } }}}}} \\ \end{array} } \right]} \right)} \\ \end{aligned}$$

Further,

$$\begin{aligned} & {\mathcalligra{f}}_{x}^{\sigma } \otimes {\mathcalligra{f}}_{y}^{\tau } = {\mathcalligra{f}}_{{([\mu_{i}^{ - } ,\mu_{i}^{ + } \left] {,[\nu_{i}^{ - } ,\nu_{i}^{ + } } \right]) \in {\mathcalligra{f}}}} \\ & \quad \left( {\begin{array}{*{20}l} {\left[ {\begin{array}{*{20}c} {\sqrt[3]{{\frac{{2\left[ {\left( {\mu_{xi}^{ - } } \right)^{3} } \right]^{\sigma } }}{{\left[ {U_{xi}^{ - } } \right]^{\sigma } + \left[ {\left( {\mu_{xi}^{ - } } \right)^{3} } \right]^{\sigma } }}}},\sqrt[3]{{\frac{{2\left[ {\left( {\mu_{xi}^{ + } } \right)^{3} } \right]^{\sigma } }}{{\left[ {U_{xi}^{ + } } \right]^{\sigma } + \left[ {\left( {\mu_{xi}^{ + } } \right)^{3} } \right]^{\sigma } }}}}} \\ \end{array} } \right],} \hfill \\ {\left[ {\begin{array}{*{20}c} {\sqrt[3]{{\frac{{\left[ {VA_{xi}^{ - } } \right]^{\sigma } - \left[ {VS_{xi}^{ - } } \right]^{\sigma } }}{{\left[ {VA_{xi}^{ - } } \right]^{\sigma } + \left[ {VS_{xi}^{ - } } \right]^{\sigma } }}}},\sqrt[3]{{\frac{{\left[ {VS_{xi}^{ + } } \right]^{\sigma } - \left[ {VS_{xi}^{ + } } \right]^{\sigma } }}{{\left[ {VS_{xi}^{ + } } \right]^{\sigma } + \left[ {VS_{xi}^{ + } } \right]^{\sigma } }}}}} \\ \end{array} } \right]} \hfill \\ \end{array} } \right) \otimes \left( {\begin{array}{*{20}l} {\left[ {\begin{array}{*{20}c} {\sqrt[3]{{\frac{{2\left[ {\left( {\mu_{yi}^{ - } } \right)^{3} } \right]^{\tau } }}{{\left[ {U_{yi}^{ - } } \right]^{\tau } + \left[ {\left( {\mu_{yi}^{ - } } \right)^{3} } \right]^{\tau } }}}},} \\ \end{array} \sqrt[3]{{\frac{{2\left[ {\left( {\mu_{yi}^{ + } } \right)^{3} } \right]^{\tau } }}{{\left[ {U_{yi}^{ + } } \right]^{\tau } + \left[ {\left( {\mu_{yi}^{ + } } \right)^{3} } \right]^{\tau } }}}}} \right],} \hfill \\ {\left[ {\begin{array}{*{20}c} {\sqrt[3]{{\frac{{\left[ {VA_{yi}^{ - } } \right]^{\tau } - \left[ {VS_{yi}^{ - } } \right]^{\tau } }}{{\left[ {VA_{yi}^{ - } } \right]^{\tau } + \left[ {VS_{yi}^{ - } } \right]^{\tau } }}}},\sqrt[3]{{\frac{{\left[ {VA_{yi}^{ + } } \right]^{\tau } - \left[ {VS_{yi}^{ + } } \right]^{\tau } }}{{\left[ {VA_{yi}^{ + } } \right]^{\tau } + \left[ {VS_{yi}^{ + } } \right]^{\tau } }}}}} \\ \end{array} } \right]} \hfill \\ \end{array} } \right) \\ & \quad = \bigcup\limits_{{([\mu_{i}^{ - } ,\mu_{i}^{ + } \left] {,[\nu_{i}^{ - } ,\nu_{i}^{ + } } \right]) \in {\mathcalligra{f}}}} {\left( {\left[ {\begin{array}{*{20}c} {\sqrt[3]{{\frac{{2\left[ {\left( {\mu_{xi}^{ - } } \right)^{3} } \right]^{\sigma } \left[ {\left( {\mu_{yi}^{ - } } \right)^{3} } \right]^{\tau } }}{{\left[ {U_{xi}^{ - } } \right]^{\sigma } \left[ {U_{yi}^{ - } } \right]^{\tau } + \left[ {\left( {\mu_{xi}^{ - } } \right)^{3} } \right]^{\sigma } \left[ {\left( {\mu_{yi}^{ - } } \right)^{3} } \right]^{\tau } }}}},} \\ {\sqrt[3]{{\frac{{2\left[ {\left( {\mu_{xi}^{ + } } \right)^{3} } \right]^{\sigma } \left[ {\left( {\mu_{yi}^{ + } } \right)^{3} } \right]^{\tau } }}{{\left[ {U_{xi}^{ + } } \right]^{\sigma } \left[ {U_{yi}^{ + } } \right]^{\tau } + \left[ {\left( {\mu_{xi}^{ + } } \right)^{3} } \right]^{\sigma } \left[ {\left( {\mu_{yi}^{ + } } \right)^{3} } \right]^{\tau } }}}}} \\ \end{array} } \right],\left[ {\begin{array}{*{20}c} {\sqrt[3]{{\frac{{\left[ {VA_{xi}^{ - } } \right]^{\sigma } \left[ {VA_{yi}^{ - } } \right]^{\tau } - \left[ {VS_{xi}^{ - } } \right]^{\sigma } \left[ {VS_{yi}^{ - } } \right]^{\tau } }}{{\left[ {VA_{xi}^{ - } } \right]^{\sigma } \left[ {VA_{yi}^{ - } } \right]^{\tau } + \left[ {VS_{xi}^{ - } } \right]^{\sigma } \left[ {VS_{yi}^{ - } } \right]^{\tau } }}}},} \\ {\sqrt[3]{{\frac{{\left[ {VA_{xi}^{ + } } \right]^{\sigma } \left[ {VA_{yi}^{ + } } \right]^{\tau } - \left[ {VS_{xi}^{ + } } \right]^{\sigma } \left[ {VS_{yi}^{ - } } \right]^{\tau } }}{{\left[ {VA_{xi}^{ + } } \right]^{\sigma } \left[ {VA_{yi}^{ + } } \right]^{\tau } + \left[ {VS_{xi}^{ + } } \right]^{\sigma } \left[ {VS_{yi}^{ + } } \right]^{\tau } }}}}} \\ \end{array} } \right]} \right)} \\ \end{aligned}$$

Finally, we have finished proving Lemma 3.1.

#### Lemma 3.2.

Assume that ${\mathcalligra{f}}_{x} = ([\mu_{xi}^{ - } ,\mu_{xi}^{ + } \left] {,[\nu_{xi}^{ - } ,\nu_{xi}^{ + } } \right])\left( {x = 1,2, \ldots ,n,i = 1,2, \ldots ,k} \right)$ consists of a group of IVFHFNs, and $\sigma ,\tau \ge 0$. Then,

3.3 $$\begin{array}{*{20}c} {\begin{array}{*{20}c} n \\ \oplus \\ {x,y = 1;x \ne y} \\ \end{array} \left( {{\mathcalligra{f}}_{x}^{\sigma } \otimes {\mathcalligra{f}}_{y}^{\tau } } \right) = \mathop {{\mathcalligra{f}}}\limits_{{([\mu_{i}^{ - } ,\mu_{i}^{ + } \left] {,[\nu_{i}^{ - } ,\nu_{i}^{ + } } \right]) \in {\mathcalligra{f}}}} \left( {\left[ {\begin{array}{*{20}c} {\sqrt[3]{{\begin{array}{*{20}c} {\frac{{\begin{array}{*{20}c} {r^{ - } - s^{ - } } \\ \end{array} }}{{\begin{array}{*{20}c} {r^{ - } + s^{ - } } \\ \end{array} }}} \\ \end{array} }},\sqrt[3]{{\begin{array}{*{20}c} {\frac{{\begin{array}{*{20}c} {r^{ + } - s^{ + } } \\ \end{array} }}{{\begin{array}{*{20}c} {r^{ + } + s^{ + } } \\ \end{array} }}} \\ \end{array} }}} \\ \end{array} } \right],\left[ {\sqrt[3]{{\frac{{2t^{ - } }}{{o^{ - } + t^{ - } }}}}, \sqrt[3]{{\frac{{2t^{ + } }}{{o^{ + } + t^{ + } }}}}} \right]} \right)} \\ \end{array}$$

where $$r^{ - } = \mathop \prod \limits_{x,y = 1;x \ne y}^{n} \begin{array}{*{20}c} {\left\{ {\left[ {2 - \left( {\mu_{xi}^{ - } } \right)^{3} } \right]^{\sigma } \left[ {2 - \left( {\mu_{yi}^{ - } } \right)^{3} } \right]^{\tau } + 3\left[ {\left( {\mu_{xi}^{ - } } \right)^{3} } \right]^{\sigma } \left[ {\left( {\mu_{yi}^{ - } } \right)^{3} } \right]^{\tau } } \right\}} \\ \end{array}$$*,* and $r^{ + }$ is to replace $-$ with $+$ in $r^{ - }$; $s^{ - } = \mathop \prod \limits_{x,y = 1;x \ne y}^{n} \left\{ {\left[ {2 - \left( {\mu_{xi}^{ - } } \right)^{3} } \right]^{\sigma } \left[ {2 - \left( {\mu_{yi}^{ - } } \right)^{3} } \right]^{\tau } - \left[ {\left( {\mu_{xi}^{ - } } \right)^{3} } \right]^{\sigma } \left[ {\left( {\mu_{yi}^{ - } } \right)^{3} } \right]^{\tau } } \right\}$*,* and $s^{ + }$ is to replace $-$ with $+$ in $s^{ - }$; $$t^{ - } = \begin{array}{*{20}c} {\mathop \prod \limits_{x,y = 1;x \ne y}^{n} \begin{array}{*{20}c} {\left\{ {\left[ {1 + \left( {\nu_{xi}^{ - } } \right)^{3} } \right]^{\sigma } \left[ {1 + \left( {\nu_{yi}^{ - } } \right)^{3} } \right]^{\tau } - \left[ {1 - \left( {\nu_{xi}^{ - } } \right)^{3} } \right]^{\sigma } \left[ {1 - \left( {\nu_{yi}^{ - } } \right)^{3} } \right]^{\tau } } \right\}} \\ \end{array} } \\ \end{array}$$*,* and $t^{ + }$ is to replace $-$ with $+$ in $t^{ - }$; $$o^{ - } = \mathop \prod \limits_{x,y = 1;x \ne y}^{n} \begin{array}{*{20}c} {\left\{ {\left[ {1 + \left( {\nu_{xi}^{ - } } \right)^{3} } \right]^{\sigma } \left[ {1 + \left( {\nu_{yi}^{ - } } \right)^{3} } \right]^{\tau } + 3\left[ {1 - \left( {\nu_{xi}^{ - } } \right)^{3} } \right]^{\sigma } \left[ {1 - \left( {\nu_{yi}^{ - } } \right)^{3} } \right]^{\tau } } \right\}} \\ \end{array}$$*,* and $o^{ + }$ is to replace $-$ with $+$ in $o^{ - }$.

#### Proof.

Similarly, we use the simplified method in Lemma 3.1 that $U_{xi}^{ - } = 2 - \left( {\mu_{xi}^{ - } } \right)^{3}$, $U_{yi}^{ - } = 2 - \left( {\mu_{yi}^{ - } } \right)^{3}$, $VA_{xi}^{ - } = 1 + \left( {\nu_{xi}^{ - } } \right)^{3}$, $VA_{yi}^{ - } = 1 + \left( {\nu_{yi}^{ - } } \right)^{3}$, $VS_{xi}^{ - } = 1 - \left( {\nu_{xi}^{ - } } \right)^{3}$, $VS_{yi}^{ - } = 1 - \left( {\nu_{yi}^{ - } } \right)^{3}$ and $U_{xi}^{ + }$, $U_{yi}^{ + }$, $VA_{xi}^{ + }$, $VA_{yi}^{ + }$, $VS_{xi}^{ + }$, $VS_{yi}^{ + }$ are to replace $-$ with $+$ in above formula. And in accordance with Lemma 3.1, we can get:

$$\begin{aligned} & {\mathcalligra{f}}_{1}^{\sigma } \otimes {\mathcalligra{f}}_{2}^{\tau } \\ & \quad = \bigcup\limits_{{([\mu_{i}^{ - } ,\mu_{i}^{ + } \left] {,[\nu_{i}^{ - } ,\nu_{i}^{ + } } \right]) \in {\mathcalligra{f}}}} {\left( {\begin{array}{*{20}l} {\left[ {\sqrt[3]{{\frac{{2\left[ {\left( {\mu_{1i}^{ - } } \right)^{3} } \right]^{\sigma } \left[ {\left( {\mu_{2i}^{ - } } \right)^{3} } \right]^{\tau } }}{{\left[ {U_{1i}^{ - } } \right]^{\sigma } \left[ {U_{2i}^{ - } } \right]^{\tau } + \left[ {\left( {\mu_{1i}^{ - } } \right)^{3} } \right]^{\sigma } \left[ {\left( {\mu_{2i}^{ - } } \right)^{3} } \right]^{\tau } }}}},\sqrt[3]{{\frac{{2\left[ {\left( {\mu_{1i}^{ + } } \right)^{3} } \right]^{\sigma } \left[ {\left( {\mu_{2i}^{ + } } \right)^{3} } \right]^{\tau } }}{{\left[ {U_{1i}^{ + } } \right]^{\sigma } \left[ {U_{2i}^{ + } } \right]^{\tau } + \left[ {\left( {\mu_{1i}^{ + } } \right)^{3} } \right]^{\sigma } \left[ {\left( {\mu_{2i}^{ + } } \right)^{3} } \right]^{\tau } }}}}} \right],} \hfill \\ {\left[ {\sqrt[3]{{\frac{{\left[ {VA_{1i}^{ - } } \right]^{\sigma } \left[ {VA_{2i}^{ - } } \right]^{\tau } - \left[ {VS_{1i}^{ - } } \right]^{\sigma } \left[ {VS_{2i}^{ - } } \right]^{\tau } }}{{\left[ {VA_{1i}^{ - } } \right]^{\sigma } \left[ {VA_{2i}^{ - } } \right]^{\tau } + \left[ {VS_{1i}^{ - } } \right]^{\sigma } \left[ {VS_{2i}^{ - } } \right]^{\tau } }}}},\sqrt[3]{{\frac{{\left[ {VA_{1i}^{ + } } \right]^{\sigma } \left[ {VA_{2i}^{ + } } \right]^{\tau } - \left[ {VS_{1i}^{ + } } \right]^{\sigma } \left[ {VS_{2i}^{ + } } \right]^{\tau } }}{{\left[ {VA_{1i}^{ + } } \right]^{\sigma } \left[ {VA_{2i}^{ + } } \right]^{\tau } + \left[ {VS_{1i}^{ + } } \right]^{\sigma } \left[ {VS_{2i}^{ + } } \right]^{\tau } }}}}} \right]} \hfill \\ \end{array} } \right)} \\ & {\mathcalligra{f}}_{2}^{\sigma } \otimes {\mathcalligra{f}}_{1}^{\tau } \\ & \quad = \bigcup\limits_{{([\mu_{i}^{ - } ,\mu_{i}^{ + } \left] {,[\nu_{i}^{ - } ,\nu_{i}^{ + } } \right]) \in {\mathcalligra{f}}}} {\left( {\begin{array}{*{20}l} {\left[ {\sqrt[3]{{\frac{{2\left[ {\left( {\mu_{2i}^{ - } } \right)^{3} } \right]^{\sigma } \left[ {\left( {\mu_{1i}^{ - } } \right)^{3} } \right]^{\tau } }}{{\left[ {U_{2i}^{ - } } \right]^{\sigma } \left[ {U_{1i}^{ - } } \right]^{\tau } + \left[ {\left( {\mu_{2i}^{ - } } \right)^{3} } \right]^{\sigma } \left[ {\left( {\mu_{1i}^{ - } } \right)^{3} } \right]^{\tau } }}}},\sqrt[3]{{\frac{{2\left[ {\left( {\mu_{2i}^{ + } } \right)^{3} } \right]^{\sigma } \left[ {\left( {\mu_{1i}^{ + } } \right)^{3} } \right]^{\tau } }}{{\left[ {U_{2i}^{ + } } \right]^{\sigma } \left[ {U_{1i}^{ + } } \right]^{\tau } + \left[ {\left( {\mu_{2i}^{ + } } \right)^{3} } \right]^{\sigma } \left[ {\left( {\mu_{1i}^{ + } } \right)^{3} } \right]^{\tau } }}}}} \right],} \hfill \\ {\left[ {\sqrt[3]{{\frac{{\left[ {VA_{2i}^{ - } } \right]^{\sigma } \left[ {VA_{1i}^{ - } } \right]^{\tau } - \left[ {VS_{2i}^{ - } } \right]^{\sigma } \left[ {VS_{1i}^{ - } } \right]^{\tau } }}{{\left[ {VA_{2i}^{ - } } \right]^{\sigma } \left[ {VA_{1i}^{ - } } \right]^{\tau } + \left[ {VS_{2i}^{ - } } \right]^{\sigma } \left[ {VS_{1i}^{ - } } \right]^{\tau } }}}},\sqrt[3]{{\frac{{\left[ {VA_{2i}^{ + } } \right]^{\sigma } \left[ {VA_{1i}^{ + } } \right]^{\tau } - \left[ {VS_{2i}^{ + } } \right]^{\sigma } \left[ {VS_{1i}^{ + } } \right]^{\tau } }}{{\left[ {VA_{2i}^{ + } } \right]^{\sigma } \left[ {VA_{1i}^{ + } } \right]^{\tau } + \left[ {VS_{2i}^{ + } } \right]^{\sigma } \left[ {VS_{1i}^{ + } } \right]^{\tau } }}}}} \right]} \hfill \\ \end{array} } \right)} \\ \end{aligned}$$

And then by supposing $n=2$, we can conclude:

$$\begin{array}{*{20}c} 2 \\ \oplus \\ {x,y = 1;x \ne y} \\ \end{array} \left( {{\mathcalligra{f}}_{x}^{\sigma } \otimes {\mathcalligra{f}}_{y}^{\tau } } \right) = \left( {{\mathcalligra{f}}_{1}^{\sigma } \otimes {\mathcalligra{f}}_{2}^{\tau } } \right) \oplus \left( {{\mathcalligra{f}}_{2}^{\sigma } \otimes {\mathcalligra{f}}_{1}^{\tau } } \right) = {\mathcalligra{f}}_{{([\mu_{i}^{ - } ,\mu_{i}^{ + } \left] {,[\nu_{i}^{ - } ,\nu_{i}^{ + } } \right]) \in {\mathcalligra{f}}}}$$

$$\left( {\begin{array}{*{20}c} {\left[ {\sqrt[3]{\begin{gathered} \left( {\begin{array}{*{20}c} {\mathop \prod \limits_{{x,y = 1;x \ne y}}^{2} \begin{array}{*{20}c} {\left\{ {\left[ {U_{{xi}}^{ - } } \right]^{\sigma } \left[ {U_{{yi}}^{ - } } \right]^{\tau } + 3\left[ {\left( {\mu _{{xi}}^{ - } } \right)^{3} } \right]^{\sigma } \left[ {\left( {\mu _{{yi}}^{ - } } \right)^{3} } \right]^{\tau } } \right\}} \\ \end{array} - } \\ {\mathop \prod \limits_{{x,y = 1;x \ne y}}^{2} \left\{ {\left[ {U_{{xi}}^{ - } } \right]^{\sigma } \left[ {U_{{yi}}^{ - } } \right]^{\tau } - \left[ {\left( {\mu _{{xi}}^{ - } } \right)^{3} } \right]^{\sigma } \left[ {\left( {\mu _{{yi}}^{ - } } \right)^{3} } \right]^{\tau } } \right\}} \\ \end{array} } \right)\cdot \hfill \\ \left( {\begin{array}{*{20}c} {\mathop \prod \limits_{{x,y = 1;x \ne y}}^{2} \begin{array}{*{20}c} {\left\{ {\left[ {U_{{xi}}^{ - } } \right]^{\sigma } \left[ {U_{{yi}}^{ - } } \right]^{\tau } + 3\left[ {\left( {\mu _{{xi}}^{ - } } \right)^{3} } \right]^{\sigma } \left[ {\left( {\mu _{{yi}}^{ - } } \right)^{3} } \right]^{\tau } } \right\}} \\ \end{array} + } \\ {\mathop \prod \limits_{{x,y = 1;x \ne y}}^{2} \left\{ {\left[ {U_{{xi}}^{ - } } \right]^{\sigma } \left[ {U_{{yi}}^{ - } } \right]^{\tau } - \left[ {\left( {\mu _{{xi}}^{ - } } \right)^{3} } \right]^{\sigma } \left[ {\left( {\mu _{{yi}}^{ - } } \right)^{3} } \right]^{\tau } } \right\}} \\ \end{array} } \right)^{{ - 1}} \hfill \\ \end{gathered} },\sqrt[3]{\begin{gathered} \left( {\begin{array}{*{20}c} {\mathop \prod \limits_{{x,y = 1;x \ne y}}^{2} \begin{array}{*{20}c} {\left\{ {\left[ {U_{{xi}}^{ + } } \right]^{\sigma } \left[ {U_{{yi}}^{ + } } \right]^{\tau } + 3\left[ {\left( {\mu _{{xi}}^{ + } } \right)^{3} } \right]^{\sigma } \left[ {\left( {\mu _{{yi}}^{ + } } \right)^{3} } \right]^{\tau } } \right\}} \\ \end{array} - } \\ {\mathop \prod \limits_{{x,y = 1;x \ne y}}^{2} \left\{ {\left[ {U_{{xi}}^{ + } } \right]^{\sigma } \left[ {U_{{yi}}^{ + } } \right]^{\tau } - \left[ {\left( {\mu _{{xi}}^{ + } } \right)^{3} } \right]^{\sigma } \left[ {\left( {\mu _{{yi}}^{ + } } \right)^{3} } \right]^{\tau } } \right\}} \\ \end{array} } \right)^{{ - 1}} \cdot \hfill \\ \left( {\begin{array}{*{20}c} {\mathop \prod \limits_{{x,y = 1;x \ne y}}^{2} \begin{array}{*{20}c} {\left\{ {\left[ {U_{{xi}}^{ + } } \right]^{\sigma } \left[ {U_{{yi}}^{ + } } \right]^{\tau } + 3\left[ {\left( {\mu _{{xi}}^{ + } } \right)^{3} } \right]^{\sigma } \left[ {\left( {\mu _{{yi}}^{ + } } \right)^{3} } \right]^{\tau } } \right\}} \\ \end{array} + } \\ {\mathop \prod \limits_{{x,y = 1;x \ne y}}^{2} \left\{ {\left[ {U_{{xi}}^{ + } } \right]^{\sigma } \left[ {U_{{yi}}^{ + } } \right]^{\tau } - \left[ {\left( {\mu _{{xi}}^{ + } } \right)^{3} } \right]^{\sigma } \left[ {\left( {\mu _{{yi}}^{ + } } \right)^{3} } \right]^{\tau } } \right\}} \\ \end{array} } \right)^{{ - 2}} \hfill \\ \end{gathered} }} \right]} \\ {\left[ {\sqrt[3]{{\begin{array}{*{20}l} {2\left( {\mathop \prod \limits_{{x,y = 1;x \ne y}}^{2} \begin{array}{*{20}c} {\left\{ {\left[ {VA_{{xi}}^{ - } } \right]^{\sigma } \left[ {VA_{{yi}}^{ - } } \right]^{\tau } - \left[ {VS_{{xi}}^{ - } } \right]^{\sigma } \left[ {VS_{{yi}}^{ - } } \right]^{\tau } } \right\}} \\ \end{array} } \right)\cdot} \hfill \\ {\left( {\begin{array}{*{20}c} {\mathop \prod \limits_{{x,y = 1;x \ne y}}^{2} \begin{array}{*{20}c} {\left\{ {\left[ {VA_{{xi}}^{ - } } \right]^{\sigma } \left[ {VA_{{yi}}^{ - } } \right]^{\tau } + 3\left[ {VS_{{xi}}^{ - } } \right]^{\sigma } \left[ {VS_{{yi}}^{ - } } \right]^{\tau } } \right\}} \\ \end{array} + } \\ {\mathop \prod \limits_{{x,y = 1;x \ne y}}^{2} \left\{ {\left[ {VA_{{xi}}^{ - } } \right]^{\sigma } \left[ {VA_{{yi}}^{ - } } \right]^{\tau } - \left[ {VS_{{xi}}^{ - } } \right]^{\sigma } \left[ {VS_{{yi}}^{ - } } \right]^{\tau } } \right\}} \\ \end{array} } \right)^{{ - 1}} } \hfill \\ \end{array} }},\sqrt[3]{{\begin{array}{*{20}l} {2\left( {\begin{array}{*{20}c} {\mathop \prod \limits_{{x,y = 1;x \ne y}}^{2} \begin{array}{*{20}c} {\left\{ {\left[ {VA_{{xi}}^{ + } } \right]^{\sigma } \left[ {VA_{{yi}}^{ + } } \right]^{\tau } - \left[ {VS_{{xi}}^{ + } } \right]^{\sigma } \left[ {VS_{{yi}}^{ + } } \right]^{\tau } } \right\}} \\ \end{array} } \\ \end{array} } \right)\cdot} \hfill \\ {\left( {\begin{array}{*{20}c} {\mathop \prod \limits_{{x,y = 1;x \ne y}}^{2} \begin{array}{*{20}c} {\left\{ {\left[ {VA_{{xi}}^{ + } } \right]^{\sigma } \left[ {VA_{{yi}}^{ + } } \right]^{\tau } + 3\left[ {VS_{{xi}}^{ + } } \right]^{\sigma } \left[ {VS_{{yi}}^{ + } } \right]^{\tau } } \right\}} \\ \end{array} + } \\ {\mathop \prod \limits_{{x,y = 1;x \ne y}}^{2} \left\{ {\left[ {VA_{{xi}}^{ + } } \right]^{\sigma } \left[ {VA_{{yi}}^{ + } } \right]^{\tau } - \left[ {VS_{{xi}}^{ + } } \right]^{\sigma } \left[ {VS_{{yi}}^{ + } } \right]^{\tau } } \right\}} \\ \end{array} } \right)^{{ - 1}} } \hfill \\ \end{array} }}} \right]} \\ \end{array} ,} \right)$$

Supposing $n = k$, the equation is as follows:

$$\begin{array}{*{20}c} k \\ \oplus \\ {x,y = 1;x \ne y} \\ \end{array} \left( {{\mathcalligra{f}}_{x}^{\sigma } \otimes {\mathcalligra{f}}_{y}^{\tau } } \right) = {\mathcalligra{f}}_{{([\mu_{i}^{ - } ,\mu_{i}^{ + } \left] {,[\nu_{i}^{ - } ,\nu_{i}^{ + } } \right]) \in {\mathcalligra{f}}}}$$

$$\left( {\begin{array}{*{20}l} {\left[ {\sqrt[3]{{\begin{array}{*{20}l} {\left( {\begin{array}{*{20}c} {\mathop \prod \limits_{{x,y = 1;x \ne y}}^{k} \begin{array}{*{20}c} {\left\{ {\left[ {U_{{xi}}^{ - } } \right]^{\sigma } \left[ {U_{{yi}}^{ - } } \right]^{\tau } + 3\left[ {\left( {\mu _{{xi}}^{ - } } \right)^{3} } \right]^{\sigma } \left[ {\left( {\mu _{{yi}}^{ - } } \right)^{3} } \right]^{\tau } } \right\}} \\ \end{array} - } \\ {\mathop \prod \limits_{{x,y = 1;x \ne y}}^{k} \left\{ {\left[ {U_{{xi}}^{ - } } \right]^{\sigma } \left[ {U_{{yi}}^{ - } } \right]^{\tau } - \left[ {\left( {\mu _{{xi}}^{ - } } \right)^{3} } \right]^{\sigma } \left[ {\left( {\mu _{{yi}}^{ - } } \right)^{3} } \right]^{\tau } } \right\}} \\ \end{array} } \right) \cdot } \hfill \\ {\left( {\begin{array}{*{20}c} {\mathop \prod \limits_{{x,y = 1;x \ne y}}^{k} \begin{array}{*{20}c} {\left\{ {\left[ {U_{{xi}}^{ - } } \right]^{\sigma } \left[ {U_{{yi}}^{ - } } \right]^{\tau } + 3\left[ {\left( {\mu _{{xi}}^{ - } } \right)^{3} } \right]^{\sigma } \left[ {\left( {\mu _{{yi}}^{ - } } \right)^{3} } \right]^{\tau } } \right\}} \\ \end{array} + } \\ {\mathop \prod \limits_{{x,y = 1;x \ne y}}^{k} \left\{ {\left[ {U_{{xi}}^{ - } } \right]^{\sigma } \left[ {U_{{yi}}^{ - } } \right]^{\tau } - \left[ {\left( {\mu _{{xi}}^{ - } } \right)^{3} } \right]^{\sigma } \left[ {\left( {\mu _{{yi}}^{ - } } \right)^{3} } \right]^{\tau } } \right\}} \\ \end{array} } \right)^{{ - 1}} } \hfill \\ \end{array} ,}}\sqrt[3]{{\begin{array}{*{20}l} {\left( {\begin{array}{*{20}c} {\mathop \prod \limits_{{x,y = 1;x \ne y}}^{k} \begin{array}{*{20}c} {\left\{ {\left[ {U_{{xi}}^{ + } } \right]^{\sigma } \left[ {U_{{yi}}^{ + } } \right]^{\tau } + 3\left[ {\left( {\mu _{{xi}}^{ + } } \right)^{3} } \right]^{\sigma } \left[ {\left( {\mu _{{yi}}^{ + } } \right)^{3} } \right]^{\tau } } \right\}} \\ \end{array} - } \\ {\mathop \prod \limits_{{x,y = 1;x \ne y}}^{k} \left\{ {\left[ {U_{{xi}}^{ + } } \right]^{\sigma } \left[ {U_{{yi}}^{ + } } \right]^{\tau } - \left[ {\left( {\mu _{{xi}}^{ + } } \right)^{3} } \right]^{\sigma } \left[ {\left( {\mu _{{yi}}^{ + } } \right)^{3} } \right]^{\tau } } \right\}} \\ \end{array} } \right)} \hfill \\ {\left( {\begin{array}{*{20}c} {\mathop \prod \limits_{{x,y = 1;x \ne y}}^{k} \begin{array}{*{20}c} {\left\{ {\left[ {U_{{xi}}^{ + } } \right]^{\sigma } \left[ {U_{{yi}}^{ + } } \right]^{\tau } + 3\left[ {\left( {\mu _{{xi}}^{ + } } \right)^{3} } \right]^{\sigma } \left[ {\left( {\mu _{{yi}}^{ + } } \right)^{3} } \right]^{\tau } } \right\}} \\ \end{array} + } \\ {\mathop \prod \limits_{{x,y = 1;x \ne y}}^{k} \left\{ {\left[ {U_{{xi}}^{ + } } \right]^{\sigma } \left[ {U_{{yi}}^{ + } } \right]^{\tau } - \left[ {\left( {\mu _{{xi}}^{ + } } \right)^{3} } \right]^{\sigma } \left[ {\left( {\mu _{{yi}}^{ + } } \right)^{3} } \right]^{\tau } } \right\}} \\ \end{array} } \right)^{{ - 1}} } \hfill \\ \end{array} }}} \right],} \hfill \\ {\left[ {\sqrt[3]{{\begin{array}{*{20}l} {2\left( {\begin{array}{*{20}c} {\mathop \prod \limits_{{x,y = 1;x \ne y}}^{k} \begin{array}{*{20}c} {\left\{ {\left[ {VA_{{xi}}^{ - } } \right]^{\sigma } \left[ {VA_{{yi}}^{ - } } \right]^{\tau } - \left[ {VS_{{xi}}^{ - } } \right]^{\sigma } \left[ {VS_{{yi}}^{ - } } \right]^{\tau } } \right\}} \\ \end{array} } \\ \end{array} } \right)\cdot} \hfill \\ {\left( {\begin{array}{*{20}c} {\mathop \prod \limits_{{x,y = 1;x \ne y}}^{k} \begin{array}{*{20}c} {\left\{ {\left[ {VA_{{xi}}^{ - } } \right]^{\sigma } \left[ {VA_{{yi}}^{ - } } \right]^{\tau } + 3\left[ {VS_{{xi}}^{ - } } \right]^{\sigma } \left[ {VS_{{yi}}^{ - } } \right]^{\tau } } \right\}} \\ \end{array} + } \\ {\mathop \prod \limits_{{x,y = 1;x \ne y}}^{k} \left\{ {\left[ {VA_{{xi}}^{ - } } \right]^{\sigma } \left[ {VA_{{yi}}^{ - } } \right]^{\tau } - \left[ {VS_{{xi}}^{ - } } \right]^{\sigma } \left[ {VS_{{yi}}^{ - } } \right]^{\tau } } \right\}} \\ \end{array} } \right)^{{ - 1}} } \hfill \\ \end{array} ,}}\sqrt[3]{{\begin{array}{*{20}l} {2\left( {\begin{array}{*{20}c} {\mathop \prod \limits_{{x,y = 1;x \ne y}}^{k} \begin{array}{*{20}c} {\left\{ {\left[ {VA_{{xi}}^{ + } } \right]^{\sigma } \left[ {VA_{{yi}}^{ + } } \right]^{\tau } - \left[ {VS_{{xi}}^{ + } } \right]^{\sigma } \left[ {VS_{{yi}}^{ + } } \right]^{\tau } } \right\}} \\ \end{array} } \\ \end{array} } \right)\cdot} \hfill \\ {\left( {\begin{array}{*{20}c} {\mathop \prod \limits_{{x,y = 1;x \ne y}}^{k} \begin{array}{*{20}c} {\left\{ {\left[ {VA_{{xi}}^{ + } } \right]^{\sigma } \left[ {VA_{{yi}}^{ + } } \right]^{\tau } + 3\left[ {VS_{{xi}}^{ + } } \right]^{\sigma } \left[ {VS_{{yi}}^{ + } } \right]^{\tau } } \right\}} \\ \end{array} + } \\ {\mathop \prod \limits_{{x,y = 1;x \ne y}}^{k} \left\{ {\left[ {VA_{{xi}}^{ + } } \right]^{\sigma } \left[ {VA_{{yi}}^{ + } } \right]^{\tau } - \left[ {VS_{{xi}}^{ + } } \right]^{\sigma } \left[ {VS_{{yi}}^{ + } } \right]^{\tau } } \right\}} \\ \end{array} } \right)^{{ - 1}} } \hfill \\ \end{array} }}} \right]} \hfill \\ \end{array} } \right)$$

when $n = k + 1$, we can conclude the equation below:

$$\begin{aligned} & \begin{array}{*{20}c} {k + 1} \\ \oplus \\ {x,y = 1;x \ne y} \\ \end{array} \left( {{\mathcalligra{f}}_{x}^{\sigma } \otimes {\mathcalligra{f}}_{y}^{\tau } } \right) \\ & \quad = \left[ {\begin{array}{*{20}c} k \\ \oplus \\ {x,y = 1;x \ne y} \\ \end{array} \left( {{\mathcalligra{f}}_{x}^{\sigma } \otimes {\mathcalligra{f}}_{y}^{\tau } } \right)} \right] \oplus \left[ {\begin{array}{*{20}c} k \\ \oplus \\ {x = 1} \\ \end{array} \left( {{\mathcalligra{f}}_{x}^{\sigma } \otimes {\mathcalligra{f}}_{k + 1}^{\tau } } \right)} \right] \oplus \left[ {\begin{array}{*{20}c} k \\ \oplus \\ {y = 1} \\ \end{array} \left( {{\mathcalligra{f}}_{k + 1}^{\sigma } \otimes {\mathcalligra{f}}_{y}^{\tau } } \right)} \right] \\ \end{aligned}$$

Firstly, according to the mathematical induction, we can obtain:

$$\begin{array}{*{20}c} k \\ \oplus \\ {x = 1} \\ \end{array} \left( {{\mathcalligra{f}}_{x}^{\sigma } \otimes {\mathcalligra{f}}_{k + 1}^{\tau } } \right) = {\mathcalligra{f}}_{{([\mu_{i}^{ - } ,\mu_{i}^{ + } \left] {,[\nu_{i}^{ - } ,\nu_{i}^{ + } } \right]) \in {\mathcalligra{f}}}}$$

$$\left( {\begin{array}{*{20}l} {\left[ {\sqrt[3]{{\begin{array}{*{20}l} {\left( {\begin{array}{*{20}c} {\mathop \prod \limits_{{x = 1}}^{k} \begin{array}{*{20}c} {\left\{ {\left[ {U_{{xi}}^{ - } } \right]^{\sigma } \left[ {U_{{\left( {k + 1} \right)i}}^{ - } } \right]^{\tau } + 3\left[ {\left( {\mu _{{xi}}^{ - } } \right)^{3} } \right]^{\sigma } \left[ {\left( {\mu _{{\left( {k + 1} \right)i}}^{ - } } \right)^{3} } \right]^{\tau } } \right\}} \\ \end{array} - } \\ {\mathop \prod \limits_{{x = 1}}^{k} \left\{ {\left[ {U_{{xi}}^{ - } } \right]^{\sigma } \left[ {U_{{\left( {k + 1} \right)i}}^{ - } } \right]^{\tau } - \left[ {\left( {\mu _{{xi}}^{ - } } \right)^{3} } \right]^{\sigma } \left[ {\left( {\mu _{{\left( {k + 1} \right)i}}^{ - } } \right)^{3} } \right]^{\tau } } \right\}} \\ \end{array} } \right)\cdot} \hfill \\ {\left( {\begin{array}{*{20}c} {\mathop \prod \limits_{{x = 1}}^{k} \begin{array}{*{20}c} {\left\{ {\left[ {U_{{xi}}^{ - } } \right]^{\sigma } \left[ {U_{{\left( {k + 1} \right)i}}^{ - } } \right]^{\tau } + 3\left[ {\left( {\mu _{{xi}}^{ - } } \right)^{3} } \right]^{\sigma } \left[ {\left( {\mu _{{\left( {k + 1} \right)i}}^{ - } } \right)^{3} } \right]^{\tau } } \right\}} \\ \end{array} + } \\ {\mathop \prod \limits_{{x = 1}}^{k} \left\{ {\left[ {U_{{xi}}^{ - } } \right]^{\sigma } \left[ {U_{{\left( {k + 1} \right)i}}^{ - } } \right]^{\tau } - \left[ {\left( {\mu _{{xi}}^{ - } } \right)^{3} } \right]^{\sigma } \left[ {\left( {\mu _{{\left( {k + 1} \right)i}}^{ - } } \right)^{3} } \right]^{\tau } } \right\}} \\ \end{array} } \right)^{{ - 1}} } \hfill \\ \end{array} ,}}\sqrt[3]{{\begin{array}{*{20}l} {\left( {\begin{array}{*{20}c} {\mathop \prod \limits_{{x = 1}}^{k} \begin{array}{*{20}c} {\left\{ {\left[ {U_{{xi}}^{ + } } \right]^{\sigma } \left[ {U_{{\left( {k + 1} \right)i}}^{ + } } \right]^{\tau } + 3\left[ {\left( {\mu _{{xi}}^{ + } } \right)^{3} } \right]^{\sigma } \left[ {\left( {\mu _{{\left( {k + 1} \right)i}}^{ + } } \right)^{3} } \right]^{\tau } } \right\}} \\ \end{array} - } \\ {\mathop \prod \limits_{{x = 1}}^{k} \left\{ {\left[ {U_{{xi}}^{ + } } \right]^{\sigma } \left[ {U_{{\left( {k + 1} \right)i}}^{ + } } \right]^{\tau } - \left[ {\left( {\mu _{{xi}}^{ + } } \right)^{3} } \right]^{\sigma } \left[ {\left( {\mu _{{\left( {k + 1} \right)i}}^{ + } } \right)^{3} } \right]^{\tau } } \right\}} \\ \end{array} } \right)\cdot} \hfill \\ {\left( {\begin{array}{*{20}c} {\mathop \prod \limits_{{x = 1}}^{k} \begin{array}{*{20}c} {\left\{ {\left[ {U_{{xi}}^{ + } } \right]^{\sigma } \left[ {U_{{\left( {k + 1} \right)i}}^{ + } } \right]^{\tau } + 3\left[ {\left( {\mu _{{xi}}^{ + } } \right)^{3} } \right]^{\sigma } \left[ {\left( {\mu _{{\left( {k + 1} \right)i}}^{ + } } \right)^{3} } \right]^{\tau } } \right\}} \\ \end{array} + } \\ {\mathop \prod \limits_{{x = 1}}^{k} \left\{ {\left[ {U_{{xi}}^{ + } } \right]^{\sigma } \left[ {U_{{\left( {k + 1} \right)i}}^{ + } } \right]^{\tau } - \left[ {\left( {\mu _{{xi}}^{ + } } \right)^{3} } \right]^{\sigma } \left[ {\left( {\mu _{{\left( {k + 1} \right)i}}^{ + } } \right)^{3} } \right]^{\tau } } \right\}} \\ \end{array} } \right)^{{ - 1}} } \hfill \\ \end{array} }}} \right],} \hfill \\ {\left[ {\sqrt[3]{{\begin{array}{*{20}l} {2\left( {\begin{array}{*{20}c} {\mathop \prod \limits_{{x = 1}}^{k} \begin{array}{*{20}c} {\left\{ {\left[ {VA_{{xi}}^{ - } } \right]^{\sigma } \left[ {VA_{{\left( {k + 1} \right)i}}^{ - } } \right]^{\tau } - \left[ {VS_{{xi}}^{ - } } \right]^{\sigma } \left[ {VS_{{\left( {k + 1} \right)i}}^{ - } } \right]^{\tau } } \right\}} \\ \end{array} } \\ \end{array} } \right)\cdot} \hfill \\ {\left( {\begin{array}{*{20}c} {\mathop \prod \limits_{{x = 1}}^{k} \begin{array}{*{20}c} {\left\{ {\left[ {VA_{{xi}}^{ - } } \right]^{\sigma } \left[ {VA_{{\left( {k + 1} \right)i}}^{ - } } \right]^{\tau } + 3\left[ {VS_{{xi}}^{ - } } \right]^{\sigma } \left[ {VS_{{\left( {k + 1} \right)i}}^{ - } } \right]^{\tau } } \right\}} \\ \end{array} + } \\ {\mathop \prod \limits_{{x = 1}}^{k} \left\{ {\left[ {VA_{{xi}}^{ - } } \right]^{\sigma } \left[ {VA_{{\left( {k + 1} \right)i}}^{ - } } \right]^{\tau } - \left[ {VS_{{xi}}^{ - } } \right]^{\sigma } \left[ {VS_{{\left( {k + 1} \right)i}}^{ - } } \right]^{\tau } } \right\}} \\ \end{array} } \right)^{{ - 1}} } \hfill \\ \end{array} ,}}\sqrt[3]{{\begin{array}{*{20}l} {2\left( {\begin{array}{*{20}c} {\mathop \prod \limits_{{x = 1}}^{k} \begin{array}{*{20}c} {\left\{ {\left[ {VA_{{xi}}^{ + } } \right]^{\sigma } \left[ {VA_{{\left( {k + 1} \right)i}}^{ + } } \right]^{\tau } - \left[ {VS_{{xi}}^{ + } } \right]^{\sigma } \left[ {VS_{{\left( {k + 1} \right)i}}^{ + } } \right]^{\tau } } \right\}} \\ \end{array} } \\ \end{array} } \right)\cdot} \hfill \\ {\left( {\begin{array}{*{20}c} {\mathop \prod \limits_{{x = 1}}^{k} \begin{array}{*{20}c} {\left\{ {\left[ {VA_{{xi}}^{ + } } \right]^{\sigma } \left[ {VA_{{\left( {k + 1} \right)i}}^{ + } } \right]^{\tau } + 3\left[ {VS_{{xi}}^{ + } } \right]^{\sigma } \left[ {VS_{{\left( {k + 1} \right)i}}^{ + } } \right]^{\tau } } \right\}} \\ \end{array} + } \\ {\mathop \prod \limits_{{x = 1}}^{k} \left\{ {\left[ {VA_{{xi}}^{ + } } \right]^{\sigma } \left[ {VA_{{\left( {k + 1} \right)i}}^{ + } } \right]^{\tau } - \left[ {VS_{{xi}}^{ + } } \right]^{\sigma } \left[ {VS_{{\left( {k + 1} \right)i}}^{ + } } \right]^{\tau } } \right\}} \\ \end{array} } \right)^{{ - 1}} } \hfill \\ \end{array} }}} \right]} \hfill \\ \end{array} } \right)$$

Secondly, we can get the following equation, similarly:

$$\begin{array}{*{20}c} k \\ \oplus \\ {y = 1} \\ \end{array} \left( {{\mathcalligra{f}}_{k + 1}^{\sigma } \otimes {\mathcalligra{f}}_{y}^{\tau } } \right) = {\mathcalligra{f}}_{{([\mu_{i}^{ - } ,\mu_{i}^{ + } \left] {,[\nu_{i}^{ - } ,\nu_{i}^{ + } } \right]) \in {\mathcalligra{f}}}}$$

$$\left( {\begin{array}{*{20}l} {\left[ {\sqrt[3]{{\begin{array}{*{20}l} {\left( {\begin{array}{*{20}c} {\mathop \prod \limits_{{y = 1}}^{k} \begin{array}{*{20}c} {\left\{ {\left[ {U_{{\left( {k + 1} \right)i}}^{ - } } \right]^{\sigma } \left[ {U_{{yi}}^{ - } } \right]^{\tau } + 3\left[ {\left( {\mu _{{\left( {k + 1} \right)i}}^{ - } } \right)^{3} } \right]^{\sigma } \left[ {\left( {\mu _{{yi}}^{ - } } \right)^{3} } \right]^{\tau } } \right\}} \\ \end{array} - } \\ {\mathop \prod \limits_{{y = 1}}^{k} \left\{ {\left[ {U_{{\left( {k + 1} \right)i}}^{ - } } \right]^{\sigma } \left[ {U_{{yi}}^{ - } } \right]^{\tau } - \left[ {\left( {\mu _{{\left( {k + 1} \right)i}}^{ - } } \right)^{3} } \right]^{\sigma } \left[ {\left( {\mu _{{yi}}^{ - } } \right)^{3} } \right]^{\tau } } \right\}} \\ \end{array} } \right)\cdot} \hfill \\ {\left( {\begin{array}{*{20}c} {\mathop \prod \limits_{{y = 1}}^{k} \begin{array}{*{20}c} {\left\{ {\left[ {U_{{\left( {k + 1} \right)i}}^{ - } } \right]^{\sigma } \left[ {U_{{yi}}^{ - } } \right]^{\tau } + 3\left[ {\left( {\mu _{{\left( {k + 1} \right)i}}^{ - } } \right)^{3} } \right]^{\sigma } \left[ {\left( {\mu _{{yi}}^{ - } } \right)^{3} } \right]^{\tau } } \right\}} \\ \end{array} + } \\ {\mathop \prod \limits_{{y = 1}}^{k} \left\{ {\left[ {U_{{\left( {k + 1} \right)i}}^{ - } } \right]^{\sigma } \left[ {U_{{yi}}^{ - } } \right]^{\tau } - \left[ {\left( {\mu _{{\left( {k + 1} \right)i}}^{ - } } \right)^{3} } \right]^{\sigma } \left[ {\left( {\mu _{{yi}}^{ - } } \right)^{3} } \right]^{\tau } } \right\}} \\ \end{array} } \right)^{{ - 1}} } \hfill \\ \end{array} ,}}\sqrt[3]{{\begin{array}{*{20}l} {\left( {\begin{array}{*{20}c} {\mathop \prod \limits_{{y = 1}}^{k} \begin{array}{*{20}c} {\left\{ {\left[ {U_{{\left( {k + 1} \right)i}}^{ + } } \right]^{\sigma } \left[ {U_{{yi}}^{ + } } \right]^{\tau } + 3\left[ {\left( {\mu _{{\left( {k + 1} \right)i}}^{ + } } \right)^{3} } \right]^{\sigma } \left[ {\left( {\mu _{{yi}}^{ + } } \right)^{3} } \right]^{\tau } } \right\}} \\ \end{array} - } \\ {\mathop \prod \limits_{{y = 1}}^{k} \left\{ {\left[ {U_{{\left( {k + 1} \right)i}}^{ + } } \right]^{\sigma } \left[ {U_{{yi}}^{ + } } \right]^{\tau } - \left[ {\left( {\mu _{{\left( {k + 1} \right)i}}^{ + } } \right)^{3} } \right]^{\sigma } \left[ {\left( {\mu _{{yi}}^{ + } } \right)^{3} } \right]^{\tau } } \right\}} \\ \end{array} } \right)\cdot} \hfill \\ {\left( {\begin{array}{*{20}c} {\mathop \prod \limits_{{y = 1}}^{k} \begin{array}{*{20}c} {\left\{ {\left[ {U_{{\left( {k + 1} \right)i}}^{ + } } \right]^{\sigma } \left[ {U_{{yi}}^{ + } } \right]^{\tau } + 3\left[ {\left( {\mu _{{\left( {k + 1} \right)i}}^{ + } } \right)^{3} } \right]^{\sigma } \left[ {\left( {\mu _{{yi}}^{ + } } \right)^{3} } \right]^{\tau } } \right\}} \\ \end{array} + } \\ {\mathop \prod \limits_{{y = 1}}^{k} \left\{ {\left[ {U_{{\left( {k + 1} \right)i}}^{ + } } \right]^{\sigma } \left[ {U_{{yi}}^{ + } } \right]^{\tau } - \left[ {\left( {\mu _{{\left( {k + 1} \right)i}}^{ + } } \right)^{3} } \right]^{\sigma } \left[ {\left( {\mu _{{yi}}^{ + } } \right)^{3} } \right]^{\tau } } \right\}} \\ \end{array} } \right)^{{ - 1}} } \hfill \\ \end{array} }}} \right],} \hfill \\ {\left[ {\sqrt[3]{{\begin{array}{*{20}l} {2\left( {\begin{array}{*{20}c} {\mathop \prod \limits_{{y = 1}}^{k} \begin{array}{*{20}c} {\left\{ {\left[ {VA_{{\left( {k + 1} \right)i}}^{ - } } \right]^{\sigma } \left[ {VA_{{yi}}^{ - } } \right]^{\tau } - \left[ {VS_{{\left( {k + 1} \right)i}}^{ - } } \right]^{\sigma } \left[ {VS_{{yi}}^{ - } } \right]^{\tau } } \right\}} \\ \end{array} } \\ \end{array} } \right)\cdot} \hfill \\ {\left( {\begin{array}{*{20}c} {\mathop \prod \limits_{{y = 1}}^{k} \begin{array}{*{20}c} {\left\{ {\left[ {VA_{{\left( {k + 1} \right)i}}^{ - } } \right]^{\sigma } \left[ {VA_{{yi}}^{ - } } \right]^{\tau } + 3\left[ {VS_{{\left( {k + 1} \right)i}}^{ - } } \right]^{\sigma } \left[ {VS_{{yi}}^{ - } } \right]^{\tau } } \right\}} \\ \end{array} + } \\ {\mathop \prod \limits_{{y = 1}}^{k} \left\{ {\left[ {VA_{{\left( {k + 1} \right)i}}^{ - } } \right]^{\sigma } \left[ {VA_{{yi}}^{ - } } \right]^{\tau } - \left[ {VS_{{\left( {k + 1} \right)i}}^{ - } } \right]^{\sigma } \left[ {VS_{{yi}}^{ - } } \right]^{\tau } } \right\}} \\ \end{array} } \right)^{{ - 1}} } \hfill \\ \end{array} ,}}\sqrt[3]{{\begin{array}{*{20}l} {2\left( {\begin{array}{*{20}c} {\mathop \prod \limits_{{y = 1}}^{k} \begin{array}{*{20}c} {\left\{ {\left[ {VA_{{\left( {k + 1} \right)i}}^{ + } } \right]^{\sigma } \left[ {VA_{{yi}}^{ + } } \right]^{\tau } - \left[ {VS_{{\left( {k + 1} \right)i}}^{ + } } \right]^{\sigma } \left[ {VS_{{yi}}^{ + } } \right]^{\tau } } \right\}} \\ \end{array} } \\ \end{array} } \right)\cdot} \hfill \\ {\left( {\begin{array}{*{20}c} {\mathop \prod \limits_{{y = 1}}^{k} \begin{array}{*{20}c} {\left\{ {\left[ {VA_{{\left( {k + 1} \right)i}}^{ + } } \right]^{\sigma } \left[ {VA_{{yi}}^{ + } } \right]^{\tau } + 3\left[ {VS_{{\left( {k + 1} \right)i}}^{ + } } \right]^{\sigma } \left[ {VS_{{yi}}^{ + } } \right]^{\tau } } \right\}} \\ \end{array} + } \\ {\mathop \prod \limits_{{y = 1}}^{k} \left\{ {\left[ {VA_{{\left( {k + 1} \right)i}}^{ + } } \right]^{\sigma } \left[ {VA_{{yi}}^{ + } } \right]^{\tau } - \left[ {VS_{{\left( {k + 1} \right)i}}^{ + } } \right]^{\sigma } \left[ {VS_{{yi}}^{ + } } \right]^{\tau } } \right\}} \\ \end{array} } \right)^{{ - 1}} } \hfill \\ \end{array} }}} \right]} \hfill \\ \end{array} } \right)$$

Therefore,

$$\begin{aligned} & \begin{array}{*{20}c} {k + 1} \\ \oplus \\ {x,y = 1;x \ne y} \\ \end{array} \left( {{\mathcalligra{f}}_{x}^{\sigma } \otimes {\mathcalligra{f}}_{y}^{\tau } } \right) \\ & \quad = \left[ {\begin{array}{*{20}c} k \\ \oplus \\ {x,y = 1;x \ne y} \\ \end{array} \left( {{\mathcalligra{f}}_{x}^{\sigma } \otimes {\mathcalligra{f}}_{y}^{\tau } } \right)} \right] \oplus \left[ {\begin{array}{*{20}c} k \\ \oplus \\ {x = 1} \\ \end{array} \left( {{\mathcalligra{f}}_{x}^{\sigma } \otimes {\mathcalligra{f}}_{k + 1}^{\tau } } \right)} \right] \oplus \left[ {\begin{array}{*{20}c} k \\ \oplus \\ {y = 1} \\ \end{array} \left( {{\mathcalligra{f}}_{k + 1}^{\sigma } \otimes {\mathcalligra{f}}_{y}^{\tau } } \right)} \right] = {\mathcalligra{f}}_{{([\mu_{i}^{ - } ,\mu_{i}^{ + } \left] {,[\nu_{i}^{ - } ,\nu_{i}^{ + } } \right]) \in {\mathcalligra{f}}}} \\ \end{aligned}$$

$$\left( {\begin{array}{*{20}l} {\left[ {\sqrt[3]{{\begin{array}{*{20}l} {\left( {\begin{array}{*{20}c} {\mathop \prod \limits_{{x,y = 1;x \ne y}}^{k} \begin{array}{*{20}c} {\left\{ {\left[ {U_{{xi}}^{ - } } \right]^{\sigma } \left[ {U_{{yi}}^{ - } } \right]^{\tau } + 3\left[ {\left( {\mu _{{xi}}^{ - } } \right)^{3} } \right]^{\sigma } \left[ {\left( {\mu _{{yi}}^{ - } } \right)^{3} } \right]^{\tau } } \right\}} \\ \end{array} - } \\ {\mathop \prod \limits_{{x,y = 1;x \ne y}}^{k} \left\{ {\left[ {U_{{xi}}^{ - } } \right]^{\sigma } \left[ {U_{{yi}}^{ - } } \right]^{\tau } - \left[ {\left( {\mu _{{xi}}^{ - } } \right)^{3} } \right]^{\sigma } \left[ {\left( {\mu _{{yi}}^{ - } } \right)^{3} } \right]^{\tau } } \right\}} \\ \end{array} } \right)\cdot} \hfill \\ {\left( {\begin{array}{*{20}c} {\mathop \prod \limits_{{x,y = 1;x \ne y}}^{k} \begin{array}{*{20}c} {\left\{ {\left[ {U_{{xi}}^{ - } } \right]^{\sigma } \left[ {U_{{yi}}^{ - } } \right]^{\tau } + 3\left[ {\left( {\mu _{{xi}}^{ - } } \right)^{3} } \right]^{\sigma } \left[ {\left( {\mu _{{yi}}^{ - } } \right)^{3} } \right]^{\tau } } \right\}} \\ \end{array} + } \\ {\mathop \prod \limits_{{x,y = 1;x \ne y}}^{k} \left\{ {\left[ {U_{{xi}}^{ - } } \right]^{\sigma } \left[ {U_{{yi}}^{ - } } \right]^{\tau } - \left[ {\left( {\mu _{{xi}}^{ - } } \right)^{3} } \right]^{\sigma } \left[ {\left( {\mu _{{yi}}^{ - } } \right)^{3} } \right]^{\tau } } \right\}} \\ \end{array} } \right)^{{ - 1}} } \hfill \\ \end{array} ,}}\sqrt[3]{{\begin{array}{*{20}l} {\left( {\begin{array}{*{20}c} {\mathop \prod \limits_{{x,y = 1;x \ne y}}^{k} \begin{array}{*{20}c} {\left\{ {\left[ {U_{{xi}}^{ + } } \right]^{\sigma } \left[ {U_{{yi}}^{ + } } \right]^{\tau } + 3\left[ {\left( {\mu _{{xi}}^{ + } } \right)^{3} } \right]^{\sigma } \left[ {\left( {\mu _{{yi}}^{ + } } \right)^{3} } \right]^{\tau } } \right\}} \\ \end{array} - } \\ {\mathop \prod \limits_{{x,y = 1;x \ne y}}^{k} \left\{ {\left[ {U_{{xi}}^{ + } } \right]^{\sigma } \left[ {U_{{yi}}^{ + } } \right]^{\tau } - \left[ {\left( {\mu _{{xi}}^{ + } } \right)^{3} } \right]^{\sigma } \left[ {\left( {\mu _{{yi}}^{ + } } \right)^{3} } \right]^{\tau } } \right\}} \\ \end{array} } \right)\cdot} \hfill \\ {\left( {\begin{array}{*{20}c} {\mathop \prod \limits_{{x,y = 1;x \ne y}}^{k} \begin{array}{*{20}c} {\left\{ {\left[ {U_{{xi}}^{ + } } \right]^{\sigma } \left[ {U_{{yi}}^{ + } } \right]^{\tau } + 3\left[ {\left( {\mu _{{xi}}^{ + } } \right)^{3} } \right]^{\sigma } \left[ {\left( {\mu _{{yi}}^{ + } } \right)^{3} } \right]^{\tau } } \right\}} \\ \end{array} + } \\ {\mathop \prod \limits_{{x,y = 1;x \ne y}}^{k} \left\{ {\left[ {U_{{xi}}^{ + } } \right]^{\sigma } \left[ {U_{{yi}}^{ + } } \right]^{\tau } - \left[ {\left( {\mu _{{xi}}^{ + } } \right)^{3} } \right]^{\sigma } \left[ {\left( {\mu _{{yi}}^{ + } } \right)^{3} } \right]^{\tau } } \right\}} \\ \end{array} } \right)^{{ - 1}} } \hfill \\ \end{array} }}} \right],} \hfill \\ {\left[ {\sqrt[3]{{\begin{array}{*{20}l} {2\left( {\begin{array}{*{20}c} {\mathop \prod \limits_{{x,y = 1;x \ne y}}^{k} \begin{array}{*{20}c} {\left\{ {\left[ {VA_{{xi}}^{ - } } \right]^{\sigma } \left[ {VA_{{yi}}^{ - } } \right]^{\tau } - \left[ {VS_{{xi}}^{ - } } \right]^{\sigma } \left[ {VS_{{yi}}^{ - } } \right]^{\tau } } \right\}} \\ \end{array} } \\ \end{array} } \right)\cdot} \hfill \\ {\left( {\begin{array}{*{20}c} {\mathop \prod \limits_{{x,y = 1;x \ne y}}^{k} \begin{array}{*{20}c} {\left\{ {\left[ {VA_{{xi}}^{ - } } \right]^{\sigma } \left[ {VA_{{yi}}^{ - } } \right]^{\tau } + 3\left[ {VS_{{xi}}^{ - } } \right]^{\sigma } \left[ {VS_{{yi}}^{ - } } \right]^{\tau } } \right\}} \\ \end{array} + } \\ {\mathop \prod \limits_{{x,y = 1;x \ne y}}^{k} \left\{ {\left[ {VA_{{xi}}^{ - } } \right]^{\sigma } \left[ {VA_{{yi}}^{ - } } \right]^{\tau } - \left[ {VS_{{xi}}^{ - } } \right]^{\sigma } \left[ {VS_{{yi}}^{ - } } \right]^{\tau } } \right\}} \\ \end{array} } \right)^{{ - 1}} } \hfill \\ \end{array} ,}}\sqrt[3]{{\begin{array}{*{20}l} {2\left( {\begin{array}{*{20}c} {\mathop \prod \limits_{{x,y = 1;x \ne y}}^{k} \begin{array}{*{20}c} {\left\{ {\left[ {VA_{{xi}}^{ + } } \right]^{\sigma } \left[ {VA_{{yi}}^{ + } } \right]^{\tau } - \left[ {VS_{{xi}}^{ + } } \right]^{\sigma } \left[ {VS_{{yi}}^{ + } } \right]^{\tau } } \right\}} \\ \end{array} } \\ \end{array} } \right)\cdot} \hfill \\ {\left( {\begin{array}{*{20}c} {\mathop \prod \limits_{{x,y = 1;x \ne y}}^{k} \begin{array}{*{20}c} {\left\{ {\left[ {VA_{{xi}}^{ + } } \right]^{\sigma } \left[ {VA_{{yi}}^{ + } } \right]^{\tau } + 3\left[ {VS_{{xi}}^{ + } } \right]^{\sigma } \left[ {VS_{{yi}}^{ + } } \right]^{\tau } } \right\}} \\ \end{array} + } \\ {\mathop \prod \limits_{{x,y = 1;x \ne y}}^{k} \left\{ {\left[ {VA_{{xi}}^{ + } } \right]^{\sigma } \left[ {VA_{{yi}}^{ + } } \right]^{\tau } - \left[ {VS_{{xi}}^{ + } } \right]^{\sigma } \left[ {VS_{{yi}}^{ + } } \right]^{\tau } } \right\}} \\ \end{array} } \right)^{{ - 1}} } \hfill \\ \end{array} }}} \right]} \hfill \\ \end{array} } \right)$$

Finally, the statement of Lemma 3.2 holds.

#### Lemma 3.3.

Assume that ${\mathcalligra{f}}_{x} = ([\mu_{xi}^{ - } ,\mu_{xi}^{ + } \left] {,[\nu_{xi}^{ - } ,\nu_{xi}^{ + } } \right])\left( {x = 1,2, \ldots ,n,i = 1,2, \ldots ,k} \right)$ consists of a group of IVFHFNs, and $\sigma ,\tau \ge 0$. we can obtain the following:

3.4 $$\begin{aligned} & \frac{1}{{n\left( {n - 1} \right)}}\left[ {\begin{array}{*{20}c} n \\ \oplus \\ {x,y = 1;x \ne y} \\ \end{array} \left( {{\mathcalligra{f}}_{x}^{\sigma } \otimes {\mathcalligra{f}}_{y}^{\tau } } \right)} \right] \\ & \quad = \bigcup\limits_{{([\mu_{i}^{ - } ,\mu_{i}^{ + } \left] {,[\nu_{i}^{ - } ,\nu_{i}^{ + } } \right]) \in {\mathcalligra{f}}}} {\left( {\begin{array}{*{20}l} {\left[ {\begin{array}{*{20}c} {\sqrt[3]{{\begin{array}{*{20}c} {\frac{{\begin{array}{*{20}c} {(r^{ - } )^{{\frac{1}{{n\left( {n - 1} \right)}}}} - (s^{ - } )^{{\frac{1}{{n\left( {n - 1} \right)}}}} } \\ \end{array} }}{{\begin{array}{*{20}c} {(r^{ - } )^{{\frac{1}{{n\left( {n - 1} \right)}}}} + (s^{ - } )^{{\frac{1}{{n\left( {n - 1} \right)}}}} } \\ \end{array} }}} \\ \end{array} }},\sqrt[3]{{\begin{array}{*{20}c} {\frac{{\begin{array}{*{20}c} {(r^{ + } )^{{\frac{1}{{n\left( {n - 1} \right)}}}} - (s^{ + } )^{{\frac{1}{{n\left( {n - 1} \right)}}}} } \\ \end{array} }}{{\begin{array}{*{20}c} {(r^{ + } )^{{\frac{1}{{n\left( {n - 1} \right)}}}} + (s^{ + } )^{{\frac{1}{{n\left( {n - 1} \right)}}}} } \\ \end{array} }}} \\ \end{array} }}} \\ \end{array} } \right],} \hfill \\ {\left[ {\sqrt[3]{{\frac{{2(t^{ - } )^{{\frac{1}{{n\left( {n - 1} \right)}}}} }}{{(o^{ - } )^{{\frac{1}{{n\left( {n - 1} \right)}}}} + (t^{ - } )^{{\frac{1}{{n\left( {n - 1} \right)}}}} }}}}, \sqrt[3]{{\frac{{2(t^{ + } )^{{\frac{1}{{n\left( {n - 1} \right)}}}} }}{{(o^{ + } )^{{\frac{1}{{n\left( {n - 1} \right)}}}} + (t^{ + } )^{{\frac{1}{{n\left( {n - 1} \right)}}}} }}}}} \right]} \hfill \\ \end{array} } \right)} \\ \end{aligned}$$

where $$r^{ - } = \mathop \prod \limits_{x,y = 1;x \ne y}^{n} \begin{array}{*{20}c} {\left\{ {\left[ {2 - \left( {\mu_{xi}^{ - } } \right)^{3} } \right]^{\sigma } \left[ {2 - \left( {\mu_{yi}^{ - } } \right)^{3} } \right]^{\tau } + 3\left[ {\left( {\mu_{xi}^{ - } } \right)^{3} } \right]^{\sigma } \left[ {\left( {\mu_{yi}^{ - } } \right)^{3} } \right]^{\tau } } \right\}} \\ \end{array}$$*,* and $r^{ + }$ is to replace $-$ with $+$ in $r^{ - }$; $s^{ - } = \mathop \prod \limits_{x,y = 1;x \ne y}^{n} \left\{ {\left[ {2 - \left( {\mu_{xi}^{ - } } \right)^{3} } \right]^{\sigma } \left[ {2 - \left( {\mu_{yi}^{ - } } \right)^{3} } \right]^{\tau } - \left[ {\left( {\mu_{xi}^{ - } } \right)^{3} } \right]^{\sigma } \left[ {\left( {\mu_{yi}^{ - } } \right)^{3} } \right]^{\tau } } \right\}$*,* and $s^{ + }$ is to replace $-$ with $+$ in $s^{ - }$; $$t^{ - } = \begin{array}{*{20}c} {\mathop \prod \limits_{x,y = 1;x \ne y}^{n} \begin{array}{*{20}c} {\left\{ {\left[ {1 + \left( {\nu_{xi}^{ - } } \right)^{3} } \right]^{\sigma } \left[ {1 + \left( {\nu_{yi}^{ - } } \right)^{3} } \right]^{\tau } - \left[ {1 - \left( {\nu_{xi}^{ - } } \right)^{3} } \right]^{\sigma } \left[ {1 - \left( {\nu_{yi}^{ - } } \right)^{3} } \right]^{\tau } } \right\}} \\ \end{array} } \\ \end{array}$$*,* and $t^{ + }$ is to replace $-$ with $+$ in $t^{ - }$; $$o^{ - } = \mathop \prod \limits_{x,y = 1;x \ne y}^{n} \begin{array}{*{20}c} {\left\{ {\left[ {1 + \left( {\nu_{xi}^{ - } } \right)^{3} } \right]^{\sigma } \left[ {1 + \left( {\nu_{yi}^{ - } } \right)^{3} } \right]^{\tau } + 3\left[ {1 - \left( {\nu_{xi}^{ - } } \right)^{3} } \right]^{\sigma } \left[ {1 - \left( {\nu_{yi}^{ - } } \right)^{3} } \right]^{\tau } } \right\}} \\ \end{array}$$*,* and $o^{ + }$ is to replace $-$ with $+$ in $o^{ - }$.

#### Proof.

On the basis of Lemma 3.2 and Definition 3.2, it can be shown as the following expression:

$$\begin{aligned} \frac{1}{{n\left( {n - 1} \right)}}\left[ {\begin{array}{*{20}c} n \\ \oplus \\ {x,y = 1;x \ne y} \\ \end{array} \left( {{\mathcalligra{f}}_{x}^{\sigma } \otimes {\mathcalligra{f}}_{y}^{\tau } } \right)} \right] = & \bigcup\limits_{{([\mu_{i}^{ - } ,\mu_{i}^{ + } \left] {,[\nu_{i}^{ - } ,\nu_{i}^{ + } } \right]) \in {\mathcalligra{f}}}} {\frac{1}{{n\left( {n - 1} \right)}}{\mathcalligra{f}}\left( {\begin{array}{*{20}c} {\left[ {\begin{array}{*{20}c} {\sqrt[3]{{\begin{array}{*{20}c} {\frac{{\begin{array}{*{20}c} {r^{ - } - s^{ - } } \\ \end{array} }}{{\begin{array}{*{20}c} {r^{ - } + s^{ - } } \\ \end{array} }}} \\ \end{array} }},\sqrt[3]{{\begin{array}{*{20}c} {\frac{{\begin{array}{*{20}c} {r^{ + } - s^{ + } } \\ \end{array} }}{{\begin{array}{*{20}c} {r^{ + } + s^{ + } } \\ \end{array} }}} \\ \end{array} }}} \\ \end{array} } \right],} \\ {\left[ {\sqrt[3]{{\frac{{2t^{ - } }}{{o^{ - } + t^{ - } }}}}, \sqrt[3]{{\frac{{2t^{ + } }}{{o^{ + } + t^{ + } }}}}} \right]} \\ \end{array} } \right)} \\ = & \bigcup\limits_{{([\mu_{i}^{ - } ,\mu_{i}^{ + } \left] {,[\nu_{i}^{ - } ,\nu_{i}^{ + } } \right]) \in {\mathcalligra{f}}}} {\left( {\begin{array}{*{20}l} {\left[ {\sqrt[3]{{\begin{array}{*{20}c} {\frac{{\begin{array}{*{20}c} {(r^{ - } )^{{\frac{1}{{n\left( {n - 1} \right)}}}} - (s^{ - } )^{{\frac{1}{{n\left( {n - 1} \right)}}}} } \\ \end{array} }}{{\begin{array}{*{20}c} {(r^{ - } )^{{\frac{1}{{n\left( {n - 1} \right)}}}} + (s^{ - } )^{{\frac{1}{{n\left( {n - 1} \right)}}}} } \\ \end{array} }}} \\ \end{array} }},\sqrt[3]{{\begin{array}{*{20}c} {\frac{{\begin{array}{*{20}c} {(r^{ + } )^{{\frac{1}{{n\left( {n - 1} \right)}}}} - (s^{ + } )^{{\frac{1}{{n\left( {n - 1} \right)}}}} } \\ \end{array} }}{{\begin{array}{*{20}c} {(r^{ + } )^{{\frac{1}{{n\left( {n - 1} \right)}}}} + (s^{ + } )^{{\frac{1}{{n\left( {n - 1} \right)}}}} } \\ \end{array} }}} \\ \end{array} }}} \right],} \hfill \\ {\left[ {\sqrt[3]{{\frac{{2(t^{ - } )^{{\frac{1}{{n\left( {n - 1} \right)}}}} }}{{(o^{ - } )^{{\frac{1}{{n\left( {n - 1} \right)}}}} + (t^{ - } )^{{\frac{1}{{n\left( {n - 1} \right)}}}} }}}}, \sqrt[3]{{\frac{{2(t^{ + } )^{{\frac{1}{{n\left( {n - 1} \right)}}}} }}{{(o^{ + } )^{{\frac{1}{{n\left( {n - 1} \right)}}}} + (t^{ + } )^{{\frac{1}{{n\left( {n - 1} \right)}}}} }}}}} \right]} \hfill \\ \end{array} } \right)} \\ \end{aligned}$$

Hence, we complete the proof of Lemma 3.3.

Combining Definition 3.3, Lemma 3.3, and the E-TNs constant power function, we can obtain the theorem of the AO of IVFHFBM conclusively:

#### Theorem 3.3.

Assume that ${\mathcalligra{f}}_{x} = ([\mu_{xi}^{ - } ,\mu_{xi}^{ + } \left] {,[\nu_{xi}^{ - } ,\nu_{xi}^{ + } } \right])\left( {x = 1,2, \ldots ,n,i = 1,2, \ldots ,k} \right)$ consists of a group of IVFHFNs, and $\sigma ,\tau \ge 0$. Further, we can yield the AO of IVFHFEBM:

3.5 $$\begin{aligned} & IVFHFEBM^{\sigma ,\tau } \left( {{\mathcalligra{f}}_{1} ,{\mathcalligra{f}}_{2} , \ldots ,{\mathcalligra{f}}_{n} } \right) = \left\{ {\frac{1}{{n\left( {n - 1} \right)}}\left[ {\begin{array}{*{20}c} n \\ \oplus \\ {x,y = 1;x \ne y} \\ \end{array} \left( {{\mathcalligra{f}}_{x}^{\sigma } \otimes {\mathcalligra{f}}_{y}^{\tau } } \right)} \right]} \right\}^{{\frac{1}{\sigma + \tau }}} \\ & \quad = \bigcup\limits_{{([\mu_{i}^{ - } ,\mu_{i}^{ + } \left] {,[\nu_{i}^{ - } ,\nu_{i}^{ + } } \right]) \in {\mathcalligra{f}}}} {\left( {\begin{array}{*{20}l} {\left[ {\begin{array}{*{20}l} {\sqrt[3]{{\frac{{2\begin{array}{*{20}c} {\left[ {(r^{ - } )^{{\frac{1}{{n\left( {n - 1} \right)}}}} - (s^{ - } )^{{\frac{1}{{n\left( {n - 1} \right)}}}} } \right]^{{\frac{1}{\sigma + \tau }}} } \\ \end{array} }}{{\begin{array}{*{20}c} {\begin{array}{*{20}c} {\left[ {(r^{ - } )^{{\frac{1}{{n\left( {n - 1} \right)}}}} + 3(s^{ - } )^{{\frac{1}{{n\left( {n - 1} \right)}}}} } \right]^{{\frac{1}{\sigma + \tau }}} } \\ \end{array} + \begin{array}{*{20}c} {\left[ {(r^{ - } )^{{\frac{1}{{n\left( {n - 1} \right)}}}} - (s^{ - } )^{{\frac{1}{{n\left( {n - 1} \right)}}}} } \right]^{{\frac{1}{\sigma + \tau }}} } \\ \end{array} } \\ \end{array} }},}}} \hfill \\ {\sqrt[3]{{\frac{{2\begin{array}{*{20}c} {\left[ {(r^{ + } )^{{\frac{1}{{n\left( {n - 1} \right)}}}} - (s^{ + } )^{{\frac{1}{{n\left( {n - 1} \right)}}}} } \right]^{{\frac{1}{\sigma + \tau }}} } \\ \end{array} }}{{\begin{array}{*{20}c} {\begin{array}{*{20}c} {\left[ {(r^{ + } )^{{\frac{1}{{n\left( {n - 1} \right)}}}} + 3(s^{ + } )^{{\frac{1}{{n\left( {n - 1} \right)}}}} } \right]^{{\frac{1}{\sigma + \tau }}} } \\ \end{array} + \begin{array}{*{20}c} {\left[ {(r^{ + } )^{{\frac{1}{{n\left( {n - 1} \right)}}}} - (s^{ + } )^{{\frac{1}{{n\left( {n - 1} \right)}}}} } \right]^{{\frac{1}{\sigma + \tau }}} } \\ \end{array} } \\ \end{array} }}}}} \hfill \\ \end{array} } \right]} \hfill \\ {\left[ {\begin{array}{*{20}l} {\sqrt[3]{{\frac{{\left[ {(o^{ - } )^{{\frac{1}{{n\left( {n - 1} \right)}}}} + 3(t^{ - } )^{{\frac{1}{{n\left( {n - 1} \right)}}}} } \right]^{{\frac{1}{\sigma + \tau }}} - \left[ {(o^{ - } )^{{\frac{1}{{n\left( {n - 1} \right)}}}} - (t^{ - } )^{{\frac{1}{{n\left( {n - 1} \right)}}}} } \right]^{{\frac{1}{\sigma + \tau }}} }}{{\left[ {(o^{ - } )^{{\frac{1}{{n\left( {n - 1} \right)}}}} + 3(t^{ - } )^{{\frac{1}{{n\left( {n - 1} \right)}}}} } \right]^{{\frac{1}{\sigma + \tau }}} + \left[ {(o^{ - } )^{{\frac{1}{{n\left( {n - 1} \right)}}}} - (t^{ - } )^{{\frac{1}{{n\left( {n - 1} \right)}}}} } \right]^{{\frac{1}{\sigma + \tau }}} }}}}} \hfill \\ {\sqrt[3]{{\frac{{\left[ {(o^{ + } )^{{\frac{1}{{n\left( {n - 1} \right)}}}} + 3(t^{ + } )^{{\frac{1}{{n\left( {n - 1} \right)}}}} } \right]^{{\frac{1}{\sigma + \tau }}} - \left[ {(o^{ + } )^{{\frac{1}{{n\left( {n - 1} \right)}}}} - (t^{ + } )^{{\frac{1}{{n\left( {n - 1} \right)}}}} } \right]^{{\frac{1}{\sigma + \tau }}} }}{{\left[ {(o^{ + } )^{{\frac{1}{{n\left( {n - 1} \right)}}}} + 3(t^{ + } )^{{\frac{1}{{n\left( {n - 1} \right)}}}} } \right]^{{\frac{1}{\sigma + \tau }}} + \left[ {(o^{ + } )^{{\frac{1}{{n\left( {n - 1} \right)}}}} - (t^{ + } )^{{\frac{1}{{n\left( {n - 1} \right)}}}} } \right]^{{\frac{1}{\sigma + \tau }}} }}}}} \hfill \\ \end{array} } \right]} \hfill \\ \end{array} } \right)} \\ \end{aligned}$$

where $$r^{ - } = \mathop \prod \limits_{x,y = 1;x \ne y}^{n} \begin{array}{*{20}c} {\left\{ {\left[ {2 - \left( {\mu_{xi}^{ - } } \right)^{3} } \right]^{\sigma } \left[ {2 - \left( {\mu_{yi}^{ - } } \right)^{3} } \right]^{\tau } + 3\left[ {\left( {\mu_{xi}^{ - } } \right)^{3} } \right]^{\sigma } \left[ {\left( {\mu_{yi}^{ - } } \right)^{3} } \right]^{\tau } } \right\}} \\ \end{array}$$*,* and $r^{ + }$ is to replace $-$ with $+$ in $r^{ - }$; $s^{ - } = \mathop \prod \limits_{x,y = 1;x \ne y}^{n} \left\{ {\left[ {2 - \left( {\mu_{xi}^{ - } } \right)^{3} } \right]^{\sigma } \left[ {2 - \left( {\mu_{yi}^{ - } } \right)^{3} } \right]^{\tau } - \left[ {\left( {\mu_{xi}^{ - } } \right)^{3} } \right]^{\sigma } \left[ {\left( {\mu_{yi}^{ - } } \right)^{3} } \right]^{\tau } } \right\}$*,* and $s^{ + }$ is to replace $-$ with $+$ in $s^{ - }$; $$t^{ - } = \begin{array}{*{20}c} {\mathop \prod \limits_{x,y = 1;x \ne y}^{n} \begin{array}{*{20}c} {\left\{ {\left[ {1 + \left( {\nu_{xi}^{ - } } \right)^{3} } \right]^{\sigma } \left[ {1 + \left( {\nu_{yi}^{ - } } \right)^{3} } \right]^{\tau } - \left[ {1 - \left( {\nu_{xi}^{ - } } \right)^{3} } \right]^{\sigma } \left[ {1 - \left( {\nu_{yi}^{ - } } \right)^{3} } \right]^{\tau } } \right\}} \\ \end{array} } \\ \end{array}$$*,* and $t^{ + }$ is to replace $-$ with $+$ in $t^{ - }$; $$o^{ - } = \mathop \prod \limits_{x,y = 1;x \ne y}^{n} \begin{array}{*{20}c} {\left\{ {\left[ {1 + \left( {\nu_{xi}^{ - } } \right)^{3} } \right]^{\sigma } \left[ {1 + \left( {\nu_{yi}^{ - } } \right)^{3} } \right]^{\tau } + 3\left[ {1 - \left( {\nu_{xi}^{ - } } \right)^{3} } \right]^{\sigma } \left[ {1 - \left( {\nu_{yi}^{ - } } \right)^{3} } \right]^{\tau } } \right\}} \\ \end{array}$$*,* and $o^{ + }$ is to replace $-$ with $+$ in $o^{ - }$.

#### Proof.

By carrying the conclusion of Lemma 3.3, we get:

$$\begin{aligned} & IVFHFEBM^{\sigma ,\tau } \left( {{\mathcalligra{f}}_{1} ,{\mathcalligra{f}}_{2} , \ldots ,{\mathcalligra{f}}_{n} } \right) = \left\{ {\frac{1}{{n\left( {n - 1} \right)}}\left[ {\begin{array}{*{20}c} n \\ \oplus \\ {x,y = 1;x \ne y} \\ \end{array} \left( {{\mathcalligra{f}}_{x}^{\sigma } \otimes {\mathcalligra{f}}_{y}^{\tau } } \right)} \right]} \right\}^{{\frac{1}{\sigma + \tau }}} \\ & \quad = \bigcup\limits_{{([\mu_{i}^{ - } ,\mu_{i}^{ + } \left] {,[\nu_{i}^{ - } ,\nu_{i}^{ + } } \right]) \in {\mathcalligra{f}}}} {\left( {\begin{array}{*{20}l} {\left[ {\sqrt[3]{{\begin{array}{*{20}c} {\frac{{\begin{array}{*{20}c} {(r^{ - } )^{{\frac{1}{{n\left( {n - 1} \right)}}}} - (s^{ - } )^{{\frac{1}{{n\left( {n - 1} \right)}}}} } \\ \end{array} }}{{\begin{array}{*{20}c} {(r^{ - } )^{{\frac{1}{{n\left( {n - 1} \right)}}}} + (s^{ - } )^{{\frac{1}{{n\left( {n - 1} \right)}}}} } \\ \end{array} }}} \\ \end{array} }},\sqrt[3]{{\begin{array}{*{20}c} {\frac{{\begin{array}{*{20}c} {(r^{ + } )^{{\frac{1}{{n\left( {n - 1} \right)}}}} - (s^{ + } )^{{\frac{1}{{n\left( {n - 1} \right)}}}} } \\ \end{array} }}{{\begin{array}{*{20}c} {(r^{ + } )^{{\frac{1}{{n\left( {n - 1} \right)}}}} + (s^{ + } )^{{\frac{1}{{n\left( {n - 1} \right)}}}} } \\ \end{array} }}} \\ \end{array} }}} \right],} \hfill \\ {\left[ {\sqrt[3]{{\frac{{2(t^{ - } )^{{\frac{1}{{n\left( {n - 1} \right)}}}} }}{{(o^{ - } )^{{\frac{1}{{n\left( {n - 1} \right)}}}} + (t^{ - } )^{{\frac{1}{{n\left( {n - 1} \right)}}}} }}}}, \sqrt[3]{{\frac{{2(t^{ + } )^{{\frac{1}{{n\left( {n - 1} \right)}}}} }}{{(o^{ + } )^{{\frac{1}{{n\left( {n - 1} \right)}}}} + (t^{ + } )^{{\frac{1}{{n\left( {n - 1} \right)}}}} }}}}} \right]} \hfill \\ \end{array} } \right)^{{1/\left( {\sigma + \tau } \right)}} } \\ & \quad = \bigcup\limits_{{([\mu_{i}^{ - } ,\mu_{i}^{ + } \left] {,[\nu_{i}^{ - } ,\nu_{i}^{ + } } \right]) \in {\mathcalligra{f}}}} {\left( {\begin{array}{*{20}l} {\left[ {\begin{array}{*{20}l} {\sqrt[3]{{\frac{{2\begin{array}{*{20}c} {\left[ {(r^{ - } )^{{\frac{1}{{n\left( {n - 1} \right)}}}} - (s^{ - } )^{{\frac{1}{{n\left( {n - 1} \right)}}}} } \right]^{{\frac{1}{\sigma + \tau }}} } \\ \end{array} }}{{\begin{array}{*{20}c} {\begin{array}{*{20}c} {\left[ {(r^{ - } )^{{\frac{1}{{n\left( {n - 1} \right)}}}} + 3(s^{ - } )^{{\frac{1}{{n\left( {n - 1} \right)}}}} } \right]^{{\frac{1}{\sigma + \tau }}} } \\ \end{array} + \begin{array}{*{20}c} {\left[ {(r^{ - } )^{{\frac{1}{{n\left( {n - 1} \right)}}}} - (s^{ - } )^{{\frac{1}{{n\left( {n - 1} \right)}}}} } \right]^{{\frac{1}{\sigma + \tau }}} } \\ \end{array} } \\ \end{array} }}}}} \hfill \\ {\sqrt[3]{{\frac{{2\begin{array}{*{20}c} {\left[ {(r^{ + } )^{{\frac{1}{{n\left( {n - 1} \right)}}}} - (s^{ + } )^{{\frac{1}{{n\left( {n - 1} \right)}}}} } \right]^{{\frac{1}{\sigma + \tau }}} } \\ \end{array} }}{{\begin{array}{*{20}c} {\begin{array}{*{20}c} {\left[ {(r^{ + } )^{{\frac{1}{{n\left( {n - 1} \right)}}}} + 3(s^{ + } )^{{\frac{1}{{n\left( {n - 1} \right)}}}} } \right]^{{\frac{1}{\sigma + \tau }}} } \\ \end{array} + \begin{array}{*{20}c} {\left[ {(r^{ + } )^{{\frac{1}{{n\left( {n - 1} \right)}}}} - (s^{ + } )^{{\frac{1}{{n\left( {n - 1} \right)}}}} } \right]^{{\frac{1}{\sigma + \tau }}} } \\ \end{array} } \\ \end{array} }}}}} \hfill \\ \end{array} } \right]} \hfill \\ {\left[ {\begin{array}{*{20}l} {\sqrt[3]{{\frac{{\left[ {(o^{ - } )^{{\frac{1}{{n\left( {n - 1} \right)}}}} + 3(t^{ - } )^{{\frac{1}{{n\left( {n - 1} \right)}}}} } \right]^{{\frac{1}{\sigma + \tau }}} - \left[ {(o^{ - } )^{{\frac{1}{{n\left( {n - 1} \right)}}}} - (t^{ - } )^{{\frac{1}{{n\left( {n - 1} \right)}}}} } \right]^{{\frac{1}{\sigma + \tau }}} }}{{\left[ {(o^{ - } )^{{\frac{1}{{n\left( {n - 1} \right)}}}} + 3(t^{ - } )^{{\frac{1}{{n\left( {n - 1} \right)}}}} } \right]^{{\frac{1}{\sigma + \tau }}} + \left[ {(o^{ - } )^{{\frac{1}{{n\left( {n - 1} \right)}}}} - (t^{ - } )^{{\frac{1}{{n\left( {n - 1} \right)}}}} } \right]^{{\frac{1}{\sigma + \tau }}} }},}}} \hfill \\ {\sqrt[3]{{\frac{{\left[ {(o^{ + } )^{{\frac{1}{{n\left( {n - 1} \right)}}}} + 3(t^{ + } )^{{\frac{1}{{n\left( {n - 1} \right)}}}} } \right]^{{\frac{1}{\sigma + \tau }}} - \left[ {(o^{ + } )^{{\frac{1}{{n\left( {n - 1} \right)}}}} - (t^{ + } )^{{\frac{1}{{n\left( {n - 1} \right)}}}} } \right]^{{\frac{1}{\sigma + \tau }}} }}{{\left[ {(o^{ + } )^{{\frac{1}{{n\left( {n - 1} \right)}}}} + 3(t^{ + } )^{{\frac{1}{{n\left( {n - 1} \right)}}}} } \right]^{{\frac{1}{\sigma + \tau }}} + \left[ {(o^{ + } )^{{\frac{1}{{n\left( {n - 1} \right)}}}} - (t^{ + } )^{{\frac{1}{{n\left( {n - 1} \right)}}}} } \right]^{{\frac{1}{\sigma + \tau }}} }}}}} \hfill \\ \end{array} } \right]} \hfill \\ \end{array} ,} \right)} \\ \end{aligned}$$

Finally, the Theorem 3.3 is demonstrated.

In the following, the corollary of the IVFHFEBM operator will be proved.

#### Corollary 1.

(Idempotency). If all of IVFHFNs ${\mathcalligra{f}}_{x} = ([\mu_{xi}^{ - } ,\mu_{xi}^{ + } \left] {,[\nu_{xi}^{ - } ,\nu_{xi}^{ + } } \right])\left( {x = 1,2, \ldots ,n,i = 1,2, \ldots ,k} \right)$ are equal such as ${\mathcalligra{f}}_{1} = {\mathcalligra{f}}_{2} = \cdots = {\mathcalligra{f}}_{n}$, we have $IVFHFEBM^{\sigma ,\tau } \left( {{\mathcalligra{f}}_{1} ,{\mathcalligra{f}}_{2} , \ldots ,{\mathcalligra{f}}_{n} } \right) = {\mathcalligra{f}} = ([\mu_{i}^{ - } ,\mu_{i}^{ + } \left] {,[\nu_{i}^{ - } ,\nu_{i}^{ + } } \right])$.

#### Proof.

Based on Theorem 3.2, owing to ${\mathcalligra{f}}_{1} = {\mathcalligra{f}}_{2} = \cdots = {\mathcalligra{f}}_{n}$, the formula in Theorem 3.3 will be varied as follows:

$$\begin{aligned} IVFHFEBM^{\sigma ,\tau } \left( {{\mathcalligra{f}}_{1} ,{\mathcalligra{f}}_{2} , \ldots ,{\mathcalligra{f}}_{n} } \right) = & \left\{ {\frac{1}{{n\left( {n - 1} \right)}}\left[ {\begin{array}{*{20}c} n \\ \oplus \\ {x,y = 1;x \ne y} \\ \end{array} \left( {{\mathcalligra{f}}_{x}^{\sigma } \otimes {\mathcalligra{f}}_{y}^{\tau } } \right)} \right]} \right\}^{{\frac{1}{\sigma + \tau }}} \\ = & \left\{ {\frac{1}{{n\left( {n - 1} \right)}}\left[ {\begin{array}{*{20}c} n \\ \oplus \\ {x,y = 1;x \ne y} \\ \end{array} \left( {{\mathcalligra{f}}^{\sigma } \otimes {\mathcalligra{f}}^{\tau } } \right)} \right]} \right\}^{{\frac{1}{\sigma + \tau }}} \\ = & \left\{ {\frac{1}{{n\left( {n - 1} \right)}}\left[ {\begin{array}{*{20}c} n \\ \oplus \\ {x,y = 1;x \ne y} \\ \end{array} {\mathcalligra{f}}^{\sigma + \tau } } \right]} \right\}^{{\frac{1}{\sigma + \tau }}} \\ = & \left\{ {\frac{1}{{n\left( {n - 1} \right)}}\left[ {n\left( {n - 1} \right){\mathcalligra{f}}^{\sigma + \tau } } \right]} \right\}^{{\frac{1}{\sigma + \tau }}} = {\mathcalligra{f}} \\ \end{aligned}$$

#### Corollary 2.

(Commutativity). If ${\mathcalligra{f}}_{1}{\prime} ,{\mathcalligra{f}}_{2}{\prime} , \ldots ,{\mathcalligra{f}}_{n}{\prime}$ are any permutation of ${\mathcalligra{f}}_{1} ,{\mathcalligra{f}}_{2} , \ldots ,{\mathcalligra{f}}_{n}$, then $IVFHFEBM^{\sigma ,\tau } \left( {{\mathcalligra{f}}_{1} ,{\mathcalligra{f}}_{2} , \ldots ,{\mathcalligra{f}}_{n} } \right) = IVFHFEBM^{\sigma ,\tau } \left( {{\mathcalligra{f}}_{1}{\prime} ,{\mathcalligra{f}}_{2}{\prime} , \ldots ,{\mathcalligra{f}}_{n}{\prime} } \right)$.

#### Proof.

Along with Theorem 3.2, prove the corollary simply. Therefore, we omit the proof.

#### Example 2.

There are three IVFHFNs, ${\mathcalligra{f}}_{1} = \left\{ {\left( {\left[ {0.6,0.8} \right],\left[ {0.4,0.5} \right]} \right),\left( {\left[ {0.5,0.8} \right],\left[ {0.1,0.3} \right]} \right)} \right\}$, ${\mathcalligra{f}}_{2} = \left\{ {\left( {\left[ {0.5,0.7} \right],\left[ {0.1,0.3} \right]} \right),\left( {\left[ {0.4,0.6} \right],\left[ {0.2,0.5} \right]} \right)} \right\}$, ${\mathcalligra{f}}_{3} = \left\{ {\left( {\left[ {0.3,0.4} \right],\left[ {0.5,0.7} \right]} \right)} \right\}$ , with parameters $\sigma = 1, \tau = 1$. By using the AO of IVFHFEWBM, we obtain the following aggregation result:

$$\begin{aligned} & IVFHFEWBM^{1,1} \left( {{\mathcalligra{f}}_{1} ,{\mathcalligra{f}}_{2} ,{\mathcalligra{f}}_{3} } \right) \\ & \quad = \left\{ {\begin{array}{*{20}c} {\left( {\left[ {0.4824,0.6630} \right],\left[ {0.3876,0.5338} \right]} \right),\left( {\left[ {0.4420,0.6223} \right],\left[ {0.3942,0.5769} \right]} \right),} \\ {\left( {\left[ {0.4437,0.0.6630} \right],\left[ {0.0.2534,0.4666} \right]} \right),\left( {\left[ {0.4055,0.6223} \right],\left[ {0.3005,0.5338} \right]} \right)} \\ \end{array} } \right\} \\ \end{aligned}$$

### The AO of IVFHFEWBM

It is common among practical problems to find that there are frequently different degrees of importance between the attributes of an object in our perception. Considering the weights of attributes is one of the things that often occurs when we are dealing with practical problems. However, as we know from Theorem 3.3, Theorem 3.3 does not take the influence of weights into account. Therefore, in order to be able to deal with such problems efficiently, we subsequently propose the IVFHFWBM operator as follows:

#### Definition 3.4.

Assume that ${\mathcalligra{f}}_{x} = ([\mu_{xi}^{ - } ,\mu_{xi}^{ + } \left] {,[\nu_{xi}^{ - } ,\nu_{xi}^{ + } } \right])\left( {x = 1,2, \ldots ,n,i = 1,2, \ldots ,k} \right)$ consists of a group of IVFHFNs, and let $\omega_{x} = \left( {\omega_{1} ,\omega_{2} , \ldots ,\omega_{n} } \right)^{T}$ be the weight vector of ${\mathcalligra{f}}_{x}$, where $\omega_{x}$ satisfies $\omega_{x} \in \left[ {0,1} \right]$ and $\mathop \sum \limits_{x = 1}^{n} \omega_{x} = 1$. Thus, the IVFHFEWBM is presented below:

3.6 $$\begin{array}{*{20}c} \begin{aligned} & IVFHFEWBM^{\sigma ,\tau } \left( {{\mathcalligra{f}}_{1} ,{\mathcalligra{f}}_{2} , \ldots ,{\mathcalligra{f}}_{n} } \right) \\ & \quad = \left( {\frac{1}{{n\left( {n - 1} \right)}}\left\{ {\begin{array}{*{20}c} n \\ \oplus \\ {x,y = 1;x \ne y} \\ \end{array} \left[ {\left( {\omega_{x} {\mathcalligra{f}}_{x} } \right)^{\sigma } \otimes \left( {\omega_{y} {\mathcalligra{f}}_{y} } \right)^{\tau } } \right]} \right\}} \right)^{{\frac{1}{\sigma + \tau }}} \\ \end{aligned} \\ \end{array}$$

where the parameters $\sigma ,\tau > 0$ and $n > 1$.

On the basis of Definition 3.3, Theorem 3.3, and the E-TNs operations on IVFHFNs, Theorem 2 is obtained.

#### Theorem 3.4.

Assume that ${\mathcalligra{f}}_{x} = ([\mu_{xi}^{ - } ,\mu_{xi}^{ + } \left] {,[\nu_{xi}^{ - } ,\nu_{xi}^{ + } } \right])\left( {x = 1,2, \ldots ,n,i = 1,2, \ldots ,k} \right)$ consists of a group of IVFHFNs, and let $\omega_{x} = \left( {\omega_{1} ,\omega_{2} , \ldots ,\omega_{n} } \right)^{T}$ be the weight vector of ${\mathcalligra{f}}_{x}$, where $\omega_{x}$ satisfies $\omega_{x} \in \left[ {0,1} \right]$ and $\sum\nolimits_{x = 1}^{n} {\omega_{x} = 1}$.

3.7 $$\begin{aligned} & IVFHFEWBM^{\sigma ,\tau } \left( {{\mathcalligra{f}}_{1} ,{\mathcalligra{f}}_{2} , \ldots ,{\mathcalligra{f}}_{n} } \right) \\ & \quad = \bigcup\limits_{{([\mu_{i}^{ - } ,\mu_{i}^{ + } \left] {,[\nu_{i}^{ - } ,\nu_{i}^{ + } } \right]) \in {\mathcalligra{f}}}} {\left( {\begin{array}{*{20}l} {\left[ {\begin{array}{*{20}l} {\sqrt[3]{{\frac{{2\begin{array}{*{20}c} {\left[ {(R^{ - } )^{{\frac{1}{{n\left( {n - 1} \right)}}}} - (S^{ - } )^{{\frac{1}{{n\left( {n - 1} \right)}}}} } \right]^{{\frac{1}{\sigma + \tau }}} } \\ \end{array} }}{{\begin{array}{*{20}c} {\begin{array}{*{20}c} {\left[ {(R^{ - } )^{{\frac{1}{{n\left( {n - 1} \right)}}}} + 3(S^{ - } )^{{\frac{1}{{n\left( {n - 1} \right)}}}} } \right]^{{\frac{1}{\sigma + \tau }}} } \\ \end{array} + \begin{array}{*{20}c} {\left[ {(R^{ - } )^{{\frac{1}{{n\left( {n - 1} \right)}}}} - (S^{ - } )^{{\frac{1}{{n\left( {n - 1} \right)}}}} } \right]^{{\frac{1}{\sigma + \tau }}} } \\ \end{array} } \\ \end{array} }},}}} \hfill \\ {\sqrt[3]{{\frac{{2\begin{array}{*{20}c} {\left[ {(R^{ + } )^{{\frac{1}{{n\left( {n - 1} \right)}}}} - (S^{ + } )^{{\frac{1}{{n\left( {n - 1} \right)}}}} } \right]^{{\frac{1}{\sigma + \tau }}} } \\ \end{array} }}{{\begin{array}{*{20}c} {\begin{array}{*{20}c} {\left[ {(R^{ + } )^{{\frac{1}{{n\left( {n - 1} \right)}}}} + 3(S^{ + } )^{{\frac{1}{{n\left( {n - 1} \right)}}}} } \right]^{{\frac{1}{\sigma + \tau }}} } \\ \end{array} + \begin{array}{*{20}c} {\left[ {(R^{ + } )^{{\frac{1}{{n\left( {n - 1} \right)}}}} - (S^{ + } )^{{\frac{1}{{n\left( {n - 1} \right)}}}} } \right]^{{\frac{1}{\sigma + \tau }}} } \\ \end{array} } \\ \end{array} }}}}} \hfill \\ \end{array} } \right],} \hfill \\ {\left[ {\begin{array}{*{20}l} {\sqrt[3]{{\frac{{\left[ {(O^{ - } )^{{\frac{1}{{n\left( {n - 1} \right)}}}} + 3(T^{ - } )^{{\frac{1}{{n\left( {n - 1} \right)}}}} } \right]^{{\frac{1}{\sigma + \tau }}} - \left[ {(O^{ - } )^{{\frac{1}{{n\left( {n - 1} \right)}}}} - (T^{ - } )^{{\frac{1}{{n\left( {n - 1} \right)}}}} } \right]^{{\frac{1}{\sigma + \tau }}} }}{{\left[ {(O^{ - } )^{{\frac{1}{{n\left( {n - 1} \right)}}}} + 3(T^{ - } )^{{\frac{1}{{n\left( {n - 1} \right)}}}} } \right]^{{\frac{1}{\sigma + \tau }}} + \left[ {(O^{ - } )^{{\frac{1}{{n\left( {n - 1} \right)}}}} - (T^{ - } )^{{\frac{1}{{n\left( {n - 1} \right)}}}} } \right]^{{\frac{1}{\sigma + \tau }}} }},}}} \hfill \\ {\sqrt[3]{{\frac{{\left[ {(O^{ + } )^{{\frac{1}{{n\left( {n - 1} \right)}}}} + 3(T^{ + } )^{{\frac{1}{{n\left( {n - 1} \right)}}}} } \right]^{{\frac{1}{\sigma + \tau }}} - \left[ {(O^{ + } )^{{\frac{1}{{n\left( {n - 1} \right)}}}} - (T^{ + } )^{{\frac{1}{{n\left( {n - 1} \right)}}}} } \right]^{{\frac{1}{\sigma + \tau }}} }}{{\left[ {(O^{ + } )^{{\frac{1}{{n\left( {n - 1} \right)}}}} + 3(T^{ + } )^{{\frac{1}{{n\left( {n - 1} \right)}}}} } \right]^{{\frac{1}{\sigma + \tau }}} + \left[ {(O^{ + } )^{{\frac{1}{{n\left( {n - 1} \right)}}}} - (T^{ + } )^{{\frac{1}{{n\left( {n - 1} \right)}}}} } \right]^{{\frac{1}{\sigma + \tau }}} }}}}} \hfill \\ \end{array} } \right]} \hfill \\ \end{array} } \right)} \\ \end{aligned}$$

where

$$R^{ - } = \prod\nolimits_{x,y = 1;x \ne y}^{n} {\left( {\begin{array}{*{20}l} {\left\{ {2 - \frac{{\left[ {1 + \left( {\mu_{xi}^{ - } } \right)^{3} } \right]^{{\omega_{x} }} - \left[ {1 - \left( {\mu_{xi}^{ - } } \right)^{3} } \right]^{{\omega_{x} }} }}{{\left[ {1 + \left( {\mu_{xi}^{ - } } \right)^{3} } \right]^{{\omega_{x} }} + \left[ {1 - \left( {\mu_{xi}^{ - } } \right)^{3} } \right]^{{\omega_{x} }} }}} \right\}^{\sigma } \left\{ {2 - \frac{{\left[ {1 + \left( {\mu_{yi}^{ - } } \right)^{3} } \right]^{{\omega_{y} }} - \left[ {1 - \left( {\mu_{yi}^{ - } } \right)^{3} } \right]^{{\omega_{y} }} }}{{\left[ {1 + \left( {\mu_{yi}^{ - } } \right)^{3} } \right]^{{\omega_{y} }} + \left[ {1 - \left( {\mu_{yi}^{ - } } \right)^{3} } \right]^{{\omega_{y} }} }}} \right\}^{\tau } + } \hfill \\ {3\left\{ {\frac{{\left[ {1 + \left( {\mu_{xi}^{ - } } \right)^{3} } \right]^{{\omega_{x} }} - \left[ {1 - \left( {\mu_{xi}^{ - } } \right)^{3} } \right]^{{\omega_{x} }} }}{{\left[ {1 + \left( {\mu_{xi}^{ - } } \right)^{3} } \right]^{{\omega_{x} }} + \left[ {1 - \left( {\mu_{xi}^{ - } } \right)^{3} } \right]^{{\omega_{x} }} }}} \right\}^{\sigma } \left\{ {\frac{{\left[ {1 + \left( {\mu_{yi}^{ - } } \right)^{3} } \right]^{{\omega_{y} }} - \left[ {1 - \left( {\mu_{yi}^{ - } } \right)^{3} } \right]^{{\omega_{y} }} }}{{\left[ {1 + \left( {\mu_{yi}^{ - } } \right)^{3} } \right]^{{\omega_{y} }} + \left[ {1 - \left( {\mu_{yi}^{ - } } \right)^{3} } \right]^{{\omega_{y} }} }}} \right\}^{\tau } } \hfill \\ \end{array} } \right)}$$ and $R^{ + }$ is to replace $-$ with $+$ in $R^{ - }$;

$$S^{ - } = \prod\nolimits_{x,y = 1;x \ne y}^{n} {\left( {\begin{array}{*{20}l} {\left\{ {1 + \frac{{2\left[ {\left( {\nu_{xi}^{ - } } \right)^{3} } \right]^{{\omega_{x} }} }}{{\left[ {2 - \left( {\nu_{xi}^{ - } } \right)^{3} } \right]^{{\omega_{x} }} + \left[ {\left( {\nu_{xi}^{ - } } \right)^{3} } \right]^{{\omega_{x} }} }}} \right\}^{\sigma } \left\{ {1 + \frac{{2\left[ {\left( {\nu_{yi}^{ - } } \right)^{3} } \right]^{{\omega_{y} }} }}{{\left[ {2 - \left( {\nu_{yi}^{ - } } \right)^{3} } \right]^{{\omega_{y} }} + \left[ {\left( {\nu_{yi}^{ - } } \right)^{3} } \right]^{{\omega_{y} }} }}} \right\}^{\tau } - } \hfill \\ {\left\{ {1 - \frac{{2\left[ {\left( {\nu_{xi}^{ - } } \right)^{3} } \right]^{{\omega_{x} }} }}{{\left[ {2 - \left( {\nu_{xi}^{ - } } \right)^{3} } \right]^{{\omega_{x} }} + \left[ {\left( {\nu_{xi}^{ - } } \right)^{3} } \right]^{{\omega_{x} }} }}} \right\}^{\sigma } \left\{ {1 - \frac{{2\left[ {\left( {\nu_{yi}^{ - } } \right)^{3} } \right]^{{\omega_{y} }} }}{{\left[ {2 - \left( {\nu_{yi}^{ - } } \right)^{3} } \right]^{{\omega_{y} }} + \left[ {\left( {\nu_{yi}^{ - } } \right)^{3} } \right]^{{\omega_{y} }} }}} \right\}^{\tau } } \hfill \\ \end{array} } \right)}$$, and $S^{ + }$ is to replace $-$ with $+$ in $S^{ - }$;

$$T^{ - } = \prod\nolimits_{x,y = 1;x \ne y}^{n} {\left( {\begin{array}{*{20}l} {\left\{ {1 + \frac{{2\left[ {\left( {\nu_{xi}^{ - } } \right)^{3} } \right]^{{\omega_{x} }} }}{{\left[ {2 - \left( {\nu_{xi}^{ - } } \right)^{3} } \right]^{{\omega_{x} }} + \left[ {\left( {\nu_{xi}^{ - } } \right)^{3} } \right]^{{\omega_{x} }} }}} \right\}^{\sigma } \left\{ {1 + \frac{{2\left[ {\left( {\nu_{yi}^{ - } } \right)^{3} } \right]^{{\omega_{y} }} }}{{\left[ {2 - \left( {\nu_{yi}^{ - } } \right)^{3} } \right]^{{\omega_{y} }} + \left[ {\left( {\nu_{yi}^{ - } } \right)^{3} } \right]^{{\omega_{y} }} }}} \right\}^{\tau } - } \hfill \\ {\left\{ {1 - \frac{{2\left[ {\left( {\nu_{xi}^{ - } } \right)^{3} } \right]^{{\omega_{x} }} }}{{\left[ {2 - \left( {\nu_{xi}^{ - } } \right)^{3} } \right]^{{\omega_{x} }} + \left[ {\left( {\nu_{xi}^{ - } } \right)^{3} } \right]^{{\omega_{x} }} }}} \right\}^{\sigma } \left\{ {1 - \frac{{2\left[ {\left( {\nu_{yi}^{ - } } \right)^{3} } \right]^{{\omega_{y} }} }}{{\left[ {2 - \left( {\nu_{yi}^{ - } } \right)^{3} } \right]^{{\omega_{y} }} + \left[ {\left( {\nu_{yi}^{ - } } \right)^{3} } \right]^{{\omega_{y} }} }}} \right\}^{\tau } } \hfill \\ \end{array} } \right)}$$, and $T^{ + }$ is to replace $-$ with $+$ in $T^{ - }$;

$$O^{ - } = \prod\nolimits_{x,y = 1;x \ne y}^{n} {\left( {\begin{array}{*{20}l} {\left\{ {1 + \frac{{2\left[ {\left( {\nu_{xi}^{ - } } \right)^{3} } \right]^{{\omega_{x} }} }}{{\left[ {2 - \left( {\nu_{xi}^{ - } } \right)^{3} } \right]^{{\omega_{x} }} + \left[ {\left( {\nu_{xi}^{ - } } \right)^{3} } \right]^{{\omega_{x} }} }}} \right\}^{\sigma } \left\{ {1 + \frac{{2\left[ {\left( {\nu_{yi}^{ - } } \right)^{3} } \right]^{{\omega_{y} }} }}{{\left[ {2 - \left( {\nu_{yi}^{ - } } \right)^{3} } \right]^{{\omega_{y} }} + \left[ {\left( {\nu_{yi}^{ - } } \right)^{3} } \right]^{{\omega_{y} }} }}} \right\}^{\tau } + } \hfill \\ {3\left\{ {1 - \frac{{2\left[ {\left( {\nu_{xi}^{ - } } \right)^{3} } \right]^{{\omega_{x} }} }}{{\left[ {2 - \left( {\nu_{xi}^{ - } } \right)^{3} } \right]^{{\omega_{x} }} + \left[ {\left( {\nu_{xi}^{ - } } \right)^{3} } \right]^{{\omega_{x} }} }}} \right\}^{\sigma } \left\{ {1 - \frac{{2\left[ {\left( {\nu_{yi}^{ - } } \right)^{3} } \right]^{{\omega_{y} }} }}{{\left[ {2 - \left( {\nu_{yi}^{ - } } \right)^{3} } \right]^{{\omega_{y} }} + \left[ {\left( {\nu_{yi}^{ - } } \right)^{3} } \right]^{{\omega_{y} }} }}} \right\}^{\tau } } \hfill \\ \end{array} } \right)}$$, and $O^{ + }$ is to replace $-$ with $+$ in $O^{ - }$.

#### Proof.

According to the Definition 3.2, the element $\omega_{x} {\mathcalligra{f}}_{x}$ is calculated as follows:

$$\omega_{x} {\mathcalligra{f}}_{x} = \bigcup\limits_{{([\mu_{i}^{ - } ,\mu_{i}^{ + } \left] {,[\nu_{i}^{ - } ,\nu_{i}^{ + } } \right]) \in {\mathcalligra{f}}}} {\left( {\begin{array}{*{20}l} {\left[ {\sqrt[3]{{\frac{{\left[ {1 + \left( {\mu_{xi}^{ - } } \right)^{3} } \right]^{{\omega_{x} }} - \left[ {1 - \left( {\mu_{xi}^{ - } } \right)^{3} } \right]^{{\omega_{x} }} }}{{\left[ {1 + \left( {\mu_{xi}^{ - } } \right)^{3} } \right]^{{\omega_{x} }} + \left[ {1 - \left( {\mu_{xi}^{ - } } \right)^{3} } \right]^{{\omega_{x} }} }}}},\sqrt[3]{{\frac{{\left[ {1 + \left( {\mu_{xi}^{ + } } \right)^{3} } \right]^{{\omega_{x} }} - \left[ {1 - \left( {\mu_{xi}^{ + } } \right)^{3} } \right]^{{\omega_{x} }} }}{{\left[ {1 + \left( {\mu_{xi}^{ + } } \right)^{3} } \right]^{{\omega_{x} }} + \left[ {1 - \left( {\mu_{xi}^{ + } } \right)^{3} } \right]^{{\omega_{x} }} }}}}} \right],} \hfill \\ {\left[ {\sqrt[3]{{\frac{{2\left[ {\left( {\nu_{xi}^{ - } } \right)^{3} } \right]^{{\omega_{x} }} }}{{\left[ {2 - \left( {\nu_{xi}^{ - } } \right)^{3} } \right]^{{\omega_{x} }} + \left[ {\left( {\nu_{xi}^{ - } } \right)^{3} } \right]^{{\omega_{x} }} }}}},\sqrt[3]{{\frac{{2\left[ {\left( {\nu_{xi}^{ + } } \right)^{3} } \right]^{{\omega_{x} }} }}{{\left[ {2 - \left( {\nu_{xi}^{ + } } \right)^{3} } \right]^{{\omega_{x} }} + \left[ {\left( {\nu_{xi}^{ + } } \right)^{3} } \right]^{{\omega_{x} }} }}}}} \right]} \hfill \\ \end{array} } \right)}$$

and the same calculation is applied to $\omega_{y} {\mathcalligra{f}}_{y}$.

Thus, we substitute ${\mathcalligra{f}}_{x} ,{\mathcalligra{f}}_{y}$ of $IVFHFEBM$ with $\omega_{x} {\mathcalligra{f}}_{x} ,\omega_{y} {\mathcalligra{f}}_{y}$, separately. Then we can obtain the following substitution formula:

$$R^{ - } = \prod\nolimits_{x,y = 1;x \ne y}^{n} {\left( {\begin{array}{*{20}l} {\left\{ {2 - \frac{{\left[ {1 + \left( {\mu_{xi}^{ - } } \right)^{3} } \right]^{{\omega_{x} }} - \left[ {1 - \left( {\mu_{xi}^{ - } } \right)^{3} } \right]^{{\omega_{x} }} }}{{\left[ {1 + \left( {\mu_{xi}^{ - } } \right)^{3} } \right]^{{\omega_{x} }} + \left[ {1 - \left( {\mu_{xi}^{ - } } \right)^{3} } \right]^{{\omega_{x} }} }}} \right\}^{\sigma } \left\{ {2 - \frac{{\left[ {1 + \left( {\mu_{yi}^{ - } } \right)^{3} } \right]^{{\omega_{y} }} - \left[ {1 - \left( {\mu_{yi}^{ - } } \right)^{3} } \right]^{{\omega_{y} }} }}{{\left[ {1 + \left( {\mu_{yi}^{ - } } \right)^{3} } \right]^{{\omega_{y} }} + \left[ {1 - \left( {\mu_{yi}^{ - } } \right)^{3} } \right]^{{\omega_{y} }} }}} \right\}^{\tau } + } \hfill \\ {3\left\{ {\frac{{\left[ {1 + \left( {\mu_{xi}^{ - } } \right)^{3} } \right]^{{\omega_{x} }} - \left[ {1 - \left( {\mu_{xi}^{ - } } \right)^{3} } \right]^{{\omega_{x} }} }}{{\left[ {1 + \left( {\mu_{xi}^{ - } } \right)^{3} } \right]^{{\omega_{x} }} + \left[ {1 - \left( {\mu_{xi}^{ - } } \right)^{3} } \right]^{{\omega_{x} }} }}} \right\}^{\sigma } \left\{ {\frac{{\left[ {1 + \left( {\mu_{yi}^{ - } } \right)^{3} } \right]^{{\omega_{y} }} - \left[ {1 - \left( {\mu_{yi}^{ - } } \right)^{3} } \right]^{{\omega_{y} }} }}{{\left[ {1 + \left( {\mu_{yi}^{ - } } \right)^{3} } \right]^{{\omega_{y} }} + \left[ {1 - \left( {\mu_{yi}^{ - } } \right)^{3} } \right]^{{\omega_{y} }} }}} \right\}^{\tau } } \hfill \\ \end{array} } \right)} ,$$

$$S^{ - } = \prod\nolimits_{x,y = 1;x \ne y}^{n} {\left( {\begin{array}{*{20}l} {\left\{ {2 - \frac{{\left[ {1 + \left( {\mu_{xi}^{ - } } \right)^{3} } \right]^{{\omega_{x} }} - \left[ {1 - \left( {\mu_{xi}^{ - } } \right)^{3} } \right]^{{\omega_{x} }} }}{{\left[ {1 + \left( {\mu_{xi}^{ - } } \right)^{3} } \right]^{{\omega_{x} }} + \left[ {1 - \left( {\mu_{xi}^{ - } } \right)^{3} } \right]^{{\omega_{x} }} }}} \right\}^{\sigma } \left\{ {2 - \frac{{\left[ {1 + \left( {\mu_{yi}^{ - } } \right)^{3} } \right]^{{\omega_{y} }} - \left[ {1 - \left( {\mu_{yi}^{ - } } \right)^{3} } \right]^{{\omega_{y} }} }}{{\left[ {1 + \left( {\mu_{yi}^{ - } } \right)^{3} } \right]^{{\omega_{y} }} + \left[ {1 - \left( {\mu_{yi}^{ - } } \right)^{3} } \right]^{{\omega_{y} }} }}} \right\}^{\tau } - } \hfill \\ {\left\{ {\frac{{\left[ {1 + \left( {\mu_{xi}^{ - } } \right)^{3} } \right]^{{\omega_{x} }} - \left[ {1 - \left( {\mu_{xi}^{ - } } \right)^{3} } \right]^{{\omega_{x} }} }}{{\left[ {1 + \left( {\mu_{xi}^{ - } } \right)^{3} } \right]^{{\omega_{x} }} + \left[ {1 - \left( {\mu_{xi}^{ - } } \right)^{3} } \right]^{{\omega_{x} }} }}} \right\}^{\sigma } \left\{ {\frac{{\left[ {1 + \left( {\mu_{yi}^{ - } } \right)^{3} } \right]^{{\omega_{y} }} - \left[ {1 - \left( {\mu_{yi}^{ - } } \right)^{3} } \right]^{{\omega_{y} }} }}{{\left[ {1 + \left( {\mu_{yi}^{ - } } \right)^{3} } \right]^{{\omega_{y} }} + \left[ {1 - \left( {\mu_{yi}^{ - } } \right)^{3} } \right]^{{\omega_{y} }} }}} \right\}^{\tau } } \hfill \\ \end{array} } \right)} ,$$

$$T^{ - } = \prod\nolimits_{x,y = 1;x \ne y}^{n} {\left( {\begin{array}{*{20}l} {\left\{ {1 + \frac{{2\left[ {\left( {\nu_{xi}^{ - } } \right)^{3} } \right]^{{\omega_{x} }} }}{{\left[ {2 - \left( {\nu_{xi}^{ - } } \right)^{3} } \right]^{{\omega_{x} }} + \left[ {\left( {\nu_{xi}^{ - } } \right)^{3} } \right]^{{\omega_{x} }} }}} \right\}^{\sigma } \left\{ {1 + \frac{{2\left[ {\left( {\nu_{yi}^{ - } } \right)^{3} } \right]^{{\omega_{y} }} }}{{\left[ {2 - \left( {\nu_{yi}^{ - } } \right)^{3} } \right]^{{\omega_{y} }} + \left[ {\left( {\nu_{yi}^{ - } } \right)^{3} } \right]^{{\omega_{y} }} }}} \right\}^{\tau } - } \hfill \\ {\left\{ {1 - \frac{{2\left[ {\left( {\nu_{xi}^{ - } } \right)^{3} } \right]^{{\omega_{x} }} }}{{\left[ {2 - \left( {\nu_{xi}^{ - } } \right)^{3} } \right]^{{\omega_{x} }} + \left[ {\left( {\nu_{xi}^{ - } } \right)^{3} } \right]^{{\omega_{x} }} }}} \right\}^{\sigma } \left\{ {1 - \frac{{2\left[ {\left( {\nu_{yi}^{ - } } \right)^{3} } \right]^{{\omega_{y} }} }}{{\left[ {2 - \left( {\nu_{yi}^{ - } } \right)^{3} } \right]^{{\omega_{y} }} + \left[ {\left( {\nu_{yi}^{ - } } \right)^{3} } \right]^{{\omega_{y} }} }}} \right\}^{\tau } } \hfill \\ \end{array} } \right)} ,$$

$$O^{ - } = \prod\nolimits_{x,y = 1;x \ne y}^{n} {\left( {\begin{array}{*{20}l} {\left\{ {1 + \frac{{2\left[ {\left( {\nu_{xi}^{ - } } \right)^{3} } \right]^{{\omega_{x} }} }}{{\left[ {2 - \left( {\nu_{xi}^{ - } } \right)^{3} } \right]^{{\omega_{x} }} + \left[ {\left( {\nu_{xi}^{ - } } \right)^{3} } \right]^{{\omega_{x} }} }}} \right\}^{\sigma } \left\{ {1 + \frac{{2\left[ {\left( {\nu_{yi}^{ - } } \right)^{3} } \right]^{{\omega_{y} }} }}{{\left[ {2 - \left( {\nu_{yi}^{ - } } \right)^{3} } \right]^{{\omega_{y} }} + \left[ {\left( {\nu_{yi}^{ - } } \right)^{3} } \right]^{{\omega_{y} }} }}} \right\}^{\tau } + } \hfill \\ {3\left\{ {1 - \frac{{2\left[ {\left( {\nu_{xi}^{ - } } \right)^{3} } \right]^{{\omega_{x} }} }}{{\left[ {2 - \left( {\nu_{xi}^{ - } } \right)^{3} } \right]^{{\omega_{x} }} + \left[ {\left( {\nu_{xi}^{ - } } \right)^{3} } \right]^{{\omega_{x} }} }}} \right\}^{\sigma } \left\{ {1 - \frac{{2\left[ {\left( {\nu_{yi}^{ - } } \right)^{3} } \right]^{{\omega_{y} }} }}{{\left[ {2 - \left( {\nu_{yi}^{ - } } \right)^{3} } \right]^{{\omega_{y} }} + \left[ {\left( {\nu_{yi}^{ - } } \right)^{3} } \right]^{{\omega_{y} }} }}} \right\}^{\tau } } \hfill \\ \end{array} } \right)} ,$$

and the calculations of $R^{ + } ,S^{ + } ,T^{ + } ,O^{ + }$ are in the same way. Hence, we omit the proof of them. Finally, the $IVFHFEWBM$ operator is performed below:

$$\begin{aligned} & IVFHFEWBM^{\sigma ,\tau } \left( {{\mathcalligra{f}}_{1} ,{\mathcalligra{f}}_{2} , \ldots ,{\mathcalligra{f}}_{n} } \right) = \left( {\frac{1}{{n\left( {n - 1} \right)}}\left\{ {\begin{array}{*{20}c} n \\ \oplus \\ {x,y = 1;x \ne y} \\ \end{array} \left[ {\left( {\omega_{x} {\mathcalligra{f}}_{x} } \right)^{\sigma } \otimes \left( {\omega_{y} {\mathcalligra{f}}_{y} } \right)^{\tau } } \right]} \right\}} \right)^{{\frac{1}{\sigma + \tau }}} \\ & \quad = \bigcup\limits_{{([\mu_{i}^{ - } ,\mu_{i}^{ + } \left] {,[\nu_{i}^{ - } ,\nu_{i}^{ + } } \right]) \in {\mathcalligra{f}}}} {\left( {\begin{array}{*{20}l} {\left[ {\begin{array}{*{20}l} {\sqrt[3]{{\frac{{2\begin{array}{*{20}c} {\left[ {(R^{ - } )^{{\frac{1}{{n\left( {n - 1} \right)}}}} - (S^{ - } )^{{\frac{1}{{n\left( {n - 1} \right)}}}} } \right]^{{\frac{1}{\sigma + \tau }}} } \\ \end{array} }}{{\begin{array}{*{20}c} {\begin{array}{*{20}c} {\left[ {(R^{ - } )^{{\frac{1}{{n\left( {n - 1} \right)}}}} + 3(S^{ - } )^{{\frac{1}{{n\left( {n - 1} \right)}}}} } \right]^{{\frac{1}{\sigma + \tau }}} } \\ \end{array} + \begin{array}{*{20}c} {\left[ {(R^{ - } )^{{\frac{1}{{n\left( {n - 1} \right)}}}} - (S^{ - } )^{{\frac{1}{{n\left( {n - 1} \right)}}}} } \right]^{{\frac{1}{\sigma + \tau }}} } \\ \end{array} } \\ \end{array} }},}}} \hfill \\ {\sqrt[3]{{\frac{{2\begin{array}{*{20}c} {\left[ {(R^{ + } )^{{\frac{1}{{n\left( {n - 1} \right)}}}} - (S^{ + } )^{{\frac{1}{{n\left( {n - 1} \right)}}}} } \right]^{{\frac{1}{\sigma + \tau }}} } \\ \end{array} }}{{\begin{array}{*{20}c} {\begin{array}{*{20}c} {\left[ {(R^{ + } )^{{\frac{1}{{n\left( {n - 1} \right)}}}} + 3(S^{ + } )^{{\frac{1}{{n\left( {n - 1} \right)}}}} } \right]^{{\frac{1}{\sigma + \tau }}} } \\ \end{array} + \begin{array}{*{20}c} {\left[ {(R^{ + } )^{{\frac{1}{{n\left( {n - 1} \right)}}}} - (S^{ + } )^{{\frac{1}{{n\left( {n - 1} \right)}}}} } \right]^{{\frac{1}{\sigma + \tau }}} } \\ \end{array} } \\ \end{array} }}}}} \hfill \\ \end{array} } \right],} \hfill \\ {\left[ {\begin{array}{*{20}l} {\sqrt[3]{{\frac{{\left[ {(O^{ - } )^{{\frac{1}{{n\left( {n - 1} \right)}}}} + 3(T^{ - } )^{{\frac{1}{{n\left( {n - 1} \right)}}}} } \right]^{{\frac{1}{\sigma + \tau }}} - \left[ {(O^{ - } )^{{\frac{1}{{n\left( {n - 1} \right)}}}} - (T^{ - } )^{{\frac{1}{{n\left( {n - 1} \right)}}}} } \right]^{{\frac{1}{\sigma + \tau }}} }}{{\left[ {(O^{ - } )^{{\frac{1}{{n\left( {n - 1} \right)}}}} + 3(T^{ - } )^{{\frac{1}{{n\left( {n - 1} \right)}}}} } \right]^{{\frac{1}{\sigma + \tau }}} + \left[ {(O^{ - } )^{{\frac{1}{{n\left( {n - 1} \right)}}}} - (T^{ - } )^{{\frac{1}{{n\left( {n - 1} \right)}}}} } \right]^{{\frac{1}{\sigma + \tau }}} }},}}} \hfill \\ {\sqrt[3]{{\frac{{\left[ {(O^{ + } )^{{\frac{1}{{n\left( {n - 1} \right)}}}} + 3(T^{ + } )^{{\frac{1}{{n\left( {n - 1} \right)}}}} } \right]^{{\frac{1}{\sigma + \tau }}} - \left[ {(O^{ + } )^{{\frac{1}{{n\left( {n - 1} \right)}}}} - (T^{ + } )^{{\frac{1}{{n\left( {n - 1} \right)}}}} } \right]^{{\frac{1}{\sigma + \tau }}} }}{{\left[ {(O^{ + } )^{{\frac{1}{{n\left( {n - 1} \right)}}}} + 3(T^{ + } )^{{\frac{1}{{n\left( {n - 1} \right)}}}} } \right]^{{\frac{1}{\sigma + \tau }}} + \left[ {(O^{ + } )^{{\frac{1}{{n\left( {n - 1} \right)}}}} - (T^{ + } )^{{\frac{1}{{n\left( {n - 1} \right)}}}} } \right]^{{\frac{1}{\sigma + \tau }}} }}}}} \hfill \\ \end{array} } \right]} \hfill \\ \end{array} } \right)} \\ \end{aligned}$$

Therefore, the statement of Theorem 3.4 holds.

#### Corollary 3.

(Commutativity). If $\omega_{1} {\mathcalligra{f}}_{1}{\prime} ,\omega_{2} {\mathcalligra{f}}_{2}{\prime} , \ldots ,\omega_{n} {\mathcalligra{f}}_{n}{\prime}$ are any permutation of $\omega_{1} {\mathcalligra{f}}_{1} ,\omega_{2} {\mathcalligra{f}}_{2} , \ldots ,\omega_{n} {\mathcalligra{f}}_{n}$, then $IVFHFEBM^{\sigma ,\tau } \left( {\omega_{1} {\mathcalligra{f}}_{1} ,\omega_{2} {\mathcalligra{f}}_{2} , \ldots ,\omega_{n} {\mathcalligra{f}}_{n} } \right) = IVFHFEBM^{\sigma ,\tau } \left( {\omega_{1} {\mathcalligra{f}}_{1}{\prime} ,\omega_{2} {\mathcalligra{f}}_{2}{\prime} , \ldots ,\omega_{n} {\mathcalligra{f}}_{n}{\prime} } \right)$.

#### Proof.

Along with Theorem 3.2, prove the corollary simply. Therefore, we omit the proof.

#### Example 3.

There are three IVFHFNs, which are the same as Example 2, ${\mathcalligra{f}}_{1} = \left\{ {\left( {\left[ {0.6,0.8} \right],\left[ {0.4,0.5} \right]} \right),\left( {\left[ {0.5,0.8} \right],\left[ {0.1,0.3} \right]} \right)} \right\}$, ${\mathcalligra{f}}_{2} = \left\{ {\left( {\left[ {0.5,0.7} \right],\left[ {0.1,0.3} \right]} \right),\left( {\left[ {0.4,0.6} \right],\left[ {0.2,0.5} \right]} \right)} \right\}$, ${\mathcalligra{f}}_{3} = \left\{ {\left( {\left[ {0.3,0.4} \right],\left[ {0.5,0.7} \right]} \right)} \right\}$, and $\omega = \left( {0.28,0.47,0.25} \right)^{T}$ is the weight vector of the three IVFHFNs, with parameters $\sigma = 1, \tau = 1$. By using the AO of IVFHFEWBM, we obtain the following aggregation result:

$$\begin{aligned} & IVFHFEWBM^{1,1} \left( {{\mathcalligra{f}}_{1} ,{\mathcalligra{f}}_{2} ,{\mathcalligra{f}}_{3} } \right) \\ & \quad = \left\{ {\begin{array}{*{20}c} {\left( {\left[ {0.3384,0.4711} \right],\left[ {0.7547,0.8299} \right]} \right),\left( {\left[ {0.3067,0.4375} \right],\left[ {0.7715,0.8590} \right]} \right),} \\ {\left( {\left[ {0.3116,0.4711} \right],\left[ {0.6664,0.7966} \right]} \right),\left( {\left[ {0.2820,0.4375} \right],\left[ {0.6921,0.8306} \right]} \right)} \\ \end{array} } \right\} \\ \end{aligned}$$

## A new MAGDM based on IVFHFEBM and IVFHFEWBM

In this section, we combine the proposed theoretical model of the IVFHFEBM and IVFHFEWBM AOs in the context of MAGDM, thus proposing a new MAGDM decision method based on the IVFHFSs environment and a corresponding procedural logic algorithm.

The detailed process steps of the new MAGDM method are as follows:

In the MAGDM environment, we assume that there are $m$ experts who have evaluated each of $l$ attributes of $j$ alternative objects involved in a project. $A = \left\{ {A_{1} ,A_{2} , \ldots ,A_{j} } \right\}$ is a discrete collection that represents alternative objects with number $j$, and $B = \left\{ {B_{1} ,B_{2} , \ldots ,B_{l} } \right\}$ is a discrete collection that represents attributes with number $l$. Each expert will evaluate each attribute of each object, and the MD and ND of the evaluated values are given subjectively by the experts, and the evaluated values are represented by IVFHFN ${\mathcalligra{f}}$, i.e., ${\mathcalligra{f}}_{ab} = ([\mu_{abi}^{ - } ,\mu_{abi}^{ + } \left] {,[\nu_{abi}^{ - } ,\nu_{abi}^{ + } } \right])$, where a denotes the $a$th object, $b$ denotes the $b$th attribute, and $i$ denotes the $i$th hesitate IVFFN. We can then construct the Interval-valued Fermatean Hesitant Fuzzy decision matrix (IVFHF-DM) of experts from the 1st to the $m$th degree, where the IVFHF-DM for the $m$th expert is expressed as $D_{j \times l}^{\gamma } = \left( {{\mathcalligra{f}}_{ab} } \right)_{j \times l}$ and is as follows:

$$\begin{aligned} & D_{j \times l}^{1} = \left[ {\begin{array}{*{20}c} {\begin{array}{*{20}c} {{\mathcalligra{f}}_{11} } & {{\mathcalligra{f}}_{12} } \\ {{\mathcalligra{f}}_{21} } & {{\mathcalligra{f}}_{22} } \\ \end{array} } & \cdots & {\begin{array}{*{20}c} {{\mathcalligra{f}}_{1l} } \\ {{\mathcalligra{f}}_{2l} } \\ \end{array} } \\ \vdots & \ddots & \vdots \\ {\begin{array}{*{20}c} {{\mathcalligra{f}}_{j1} } & {{\mathcalligra{f}}_{j2} } \\ \end{array} } & \cdots & {{\mathcalligra{f}}_{jl} } \\ \end{array} } \right] \\ & D_{j \times l}^{2} = \left[ {\begin{array}{*{20}c} {\begin{array}{*{20}c} {{\mathcalligra{f}}_{11} } & {{\mathcalligra{f}}_{12} } \\ {{\mathcalligra{f}}_{21} } & {{\mathcalligra{f}}_{22} } \\ \end{array} } & \cdots & {\begin{array}{*{20}c} {{\mathcalligra{f}}_{1l} } \\ {{\mathcalligra{f}}_{2l} } \\ \end{array} } \\ \vdots & \ddots & \vdots \\ {\begin{array}{*{20}c} {{\mathcalligra{f}}_{j1} } & {{\mathcalligra{f}}_{j2} } \\ \end{array} } & \cdots & {{\mathcalligra{f}}_{jl} } \\ \end{array} } \right] \\ & \quad \quad \quad \quad \quad \vdots \\ & D_{j \times l}^{m} = \left[ {\begin{array}{*{20}c} {\begin{array}{*{20}c} {{\mathcalligra{f}}_{11} } & {{\mathcalligra{f}}_{12} } \\ {{\mathcalligra{f}}_{21} } & {{\mathcalligra{f}}_{22} } \\ \end{array} } & \cdots & {\begin{array}{*{20}c} {{\mathcalligra{f}}_{1l} } \\ {{\mathcalligra{f}}_{2l} } \\ \end{array} } \\ \vdots & \ddots & \vdots \\ {\begin{array}{*{20}c} {{\mathcalligra{f}}_{j1} } & {{\mathcalligra{f}}_{j2} } \\ \end{array} } & \cdots & {{\mathcalligra{f}}_{jl} } \\ \end{array} } \right] \\ \end{aligned}$$

*Step 1* Having the IVFHF-DMs containing the number of experts with $m$, we need to take into account the assessments of all the experts and integrate their assessments. To reduce the loss of information on aggregation when aggregating expert opinions, the rule for integration is that when none of the experts' IVFHFN ${\mathcalligra{f}}$ assessments agree, we keep all the different data, and when there is partial agreement, only one of the same assessment values is kept. Therefore, we can acquire a new IVFHN ${\mathcalligra{f}}_{ab} = ([\mu_{abi}^{ - } ,\mu_{abi}^{ + } \left] {,[\nu_{abi}^{ - } ,\nu_{abi}^{ + } } \right])$. For example, there are three IVFFNs, which are ${\mathcalligra{f}}_{1} = \left\{ {\left( {\left[ {0.6,0.7} \right],\left[ {0.1,0.2} \right]} \right),\left( {\left[ {0.5,0.8} \right],\left[ {0.3,0.5} \right]} \right)} \right\}$, ${\mathcalligra{f}}_{2} = \left\{ {\left( {\left[ {0.5,0.8} \right],\left[ {0.3,0.5} \right]} \right),\left( {\left[ {0.5,0.7} \right],\left[ {0.4,0.6} \right]} \right)} \right\}$ and ${\mathcalligra{f}}_{3} = \left( {\left[ {0.6,0.7} \right],\left[ {0.1,0.2} \right]} \right)$. Here, three IVFHFNs are integrated into one IVFHN, which is represented as ${\mathcalligra{f}} = \left\{ {\left( {\left[ {0.6,0.7} \right],\left[ {0.1,0.2} \right]} \right),\left( {\left[ {0.5,0.8} \right],\left[ {0.3,0.5} \right]} \right),\left( {\left[ {0.5,0.7} \right],\left[ {0.4,0.6} \right]} \right)} \right\}$. This allows us to obtain an Integrate IVFHF-DM $D_{j \times l}{\prime} = \left( {{\mathcalligra{f}}_{ab}{\prime} } \right)_{j \times l}$ that combines all the experts’ evaluations:

$$D_{j \times l}{\prime} = \left[ {\begin{array}{*{20}c} {\begin{array}{*{20}c} {{\mathcalligra{f}}_{11}{\prime} } & {{\mathcalligra{f}}_{12}{\prime} } \\ {{\mathcalligra{f}}_{21}{\prime} } & {{\mathcalligra{f}}_{22}{\prime} } \\ \end{array} } & \cdots & {\begin{array}{*{20}c} {{\mathcalligra{f}}_{1l}{\prime} } \\ {{\mathcalligra{f}}_{2l}{\prime} } \\ \end{array} } \\ \vdots & \ddots & \vdots \\ {\begin{array}{*{20}c} {{\mathcalligra{f}}_{j1}{\prime} } & {{\mathcalligra{f}}_{j2}{\prime} } \\ \end{array} } & \cdots & {{\mathcalligra{f}}_{jl}{\prime} } \\ \end{array} } \right]$$

*Step 2* Considering that attributes are not only benefit attributes but also cost attributes when making decisions, we need to normalize the IVFHF-DM matrix. The purpose of the normalization process is to convert all attributes into benefit attributes in a uniform way so that the attributes can be processed later. All cost attributes are converted to benefit attributes by the complementary operation in Definition 3.2. The formula for the conversion is as follows:

4.1 $$\begin{array}{*{20}c} {{\mathcalligra{f}}_{ab}^{^{\prime\prime}} = \left\{ {\begin{array}{*{20}l} {{\mathcalligra{f}}_{ab}{\prime} } \hfill & {for \;benefit \;attribute\; B_{b} } \hfill \\ {({\mathcalligra{f}}_{ab}{\prime} )^{C} } \hfill & { for\; cost\; attribute\; B_{b} } \hfill \\ \end{array} } \right.} \\ \end{array}$$

where $a = 1,2, \ldots ,j$ and $b = 1,2, \ldots ,l$. $({\mathcalligra{f}}_{ab}{\prime} )^{C}$ is the complement of ${\mathcalligra{f}}_{ab}{\prime}$. Thus, we can obtain a normalized IVFHF-DM $D_{j \times l}^{^{\prime\prime}} = \left( {{\mathcalligra{f}}_{ab}^{^{\prime\prime}} } \right)_{j \times l}$

$$D_{j \times l}^{^{\prime\prime}} = \left[ {\begin{array}{*{20}c} {\begin{array}{*{20}c} {{\mathcalligra{f}}_{11}^{^{\prime\prime}} } & {{\mathcalligra{f}}_{12}^{^{\prime\prime}} } \\ {{\mathcalligra{f}}_{21}^{^{\prime\prime}} } & {{\mathcalligra{f}}_{22}^{^{\prime\prime}} } \\ \end{array} } & \cdots & {\begin{array}{*{20}c} {{\mathcalligra{f}}_{1l}^{^{\prime\prime}} } \\ {{\mathcalligra{f}}_{2l}^{^{\prime\prime}} } \\ \end{array} } \\ \vdots & \ddots & \vdots \\ {\begin{array}{*{20}c} {{\mathcalligra{f}}_{j1}^{^{\prime\prime}} } & {{\mathcalligra{f}}_{j2}^{^{\prime\prime}} } \\ \end{array} } & \cdots & {{\mathcalligra{f}}_{jl}^{^{\prime\prime}} } \\ \end{array} } \right]$$

*Step 3* If each attribute of the decision matrix does not have a weight, we use the AO of IVFHFEBM in Theorem 3.3 to aggregate all the attribute evaluations of each alternative object into a single evaluation value in IVFHFN; if each attribute of the decision matrix has a weight, the weight vector is denoted as $\omega = \left( {\omega_{1} ,\omega_{2} , \ldots ,\omega_{l} } \right)^{T}$, so we can utilize the AO of IVFHFEWBM in Theorem 3.4 to aggregate the evaluated values of all the attributes of each object, as follows:

4.2 $$\begin{array}{*{20}c} {{\mathcalligra{f}}_{a}^{{^{\prime\prime}}} = IVFHFEBM^{\sigma ,\tau } \left( {{\mathcalligra{f}}_{a1}^{{^{\prime\prime}}} ,{\mathcalligra{f}}_{a2}^{{^{\prime\prime}}} , \ldots ,{\mathcalligra{f}}_{al}^{{^{\prime\prime}}} } \right)} \\ \end{array}$$

4.3 $$\begin{array}{*{20}c} {{\mathcalligra{f}}_{a}^{{^{\prime\prime}}} = IVFHFEWBM^{\sigma ,\tau } \left( {{\mathcalligra{f}}_{a1}^{{^{\prime\prime}}} ,{\mathcalligra{f}}_{a2}^{{^{\prime\prime}}} , \ldots ,{\mathcalligra{f}}_{al}^{{^{\prime\prime}}} } \right)} \\ \end{array}$$

where $$a = 1,2, \ldots ,j$$.

*Step 4* The SC and AC proposed in Definition 2.5 are used to calculate the score and accuracy values for each object, respectively.

*Step 5* We can perform a descending sort on each object using the comparison rules of the P function in Definition 2.6.

**Table:** 

Our algorithm: the novel MAGDM based on IVFHFEBM and IVFHFEWBM and proposed SC:	
**Input:** A MAGDM system that includes $m$ IVFHF-DM $D_{j \times l}^{m} = \left( {{\mathcalligra{f}}_{ab} } \right)_{j \times l}$ matrices and the values of the parameters $\sigma$ and $\tau$. The weight vector is added if there is a weight parameter; otherwise, it is omitted **Output:** The results of the descending sorting of the alternative objects **Begin** 1: **for** $A_{a} \in \left\{ {A_{1} ,A_{2} , \ldots ,A_{j} } \right\}$, $B_{b} \in \left\{ {B_{1} ,B_{2} , \ldots ,B_{l} } \right\}$ **do** 2: Integrate: Integrate the IVFHF-DM $D_{j \times l}^{m}$ matrices of $m$ into one $D_{j \times l}{\prime}$ matrix according to Step 1 above 3: **end for** $A_{a} \in \left\{ {A_{1} ,A_{2} , \ldots ,A_{j} } \right\}$, $B_{b} \in \left\{ {B_{1} ,B_{2} , \ldots ,B_{l} } \right\}$ **do** 4: Normalize: Convert the matrix $D_{j \times l}{\prime}$ to $D_{j \times l}^{^{\prime\prime}}$ by converting ${\mathcalligra{f}}_{ab}{\prime}$ to ${\mathcalligra{f}}_{ab}^{^{\prime\prime}}$ by Eq. (4.1) 5: **end for** $A_{a} \in \left\{ {A_{1} ,A_{2} , \ldots ,A_{j} } \right\}$ **do** 6: Aggregate: If there is no weight vector, aggregate IVFHFNs of each object involving all of the attributes by Eq. (4.2); if the weight vector exists, aggregate them by Eq. (4.3) 7: **end for** $A_{a} \in \left\{ {A_{1} ,A_{2} , \ldots ,A_{j} } \right\}$ **do** 8: Compute: Obtain the SC and the AC by Eq. (2.1) and Eq. (2.2) 9: **end for** $A_{a} \in \left\{ {A_{1} ,A_{2} , \ldots ,A_{j} } \right\}$ **do** 10: Rank: Sort all the alternative objects by Eq. (2.3) 11: **end** 12: **Return:** The ranking results are sorted in descending order of alternative objects	

## Case study and comparative analysis

In the current section, our proposed AOs address the medical diagnostic MAGDM problem of cardiovascular disease under the IVFHFSs environment. (1) We state the background and significance of the experimental study and the advantages of the case study of IVFHFSs in this context. (2) We illustrate a medical diagnostic evaluation case study of cardiovascular disease using the proposed MAGDM method. (3) The evaluation of cardiovascular disease diagnosis then yields a decision ranking result. (4) We examine the sensitivity of the proposed method by adjusting the variable parameters in the IVFHFEBM and IVFHFEWBM operators and investigate the effect of the parameter transformations on the ultimate decision results. (5) We compare the proposed method to other current MAGDM methods in the context of the medical diagnostic MAGDM problem of cardiovascular disease and verify the suggested method's efficacy and dependability. (6) In the end, we discuss and summarize the benefits of the proposed method in a tabular format.

### The background of cardiovascular disease diagnosis based on IVFHFSs-MAGDM

In recent years, non-communicable diseases have continued to account for a high proportion of the world's top 10 causes of death, with cardiovascular disease topping the list. Cardiovascular disease is the top killer of human health. According to WHO, nearly 17 million people die of cardiovascular disease each year^42^. Obviously, the degree of rapid and effective diagnosis of cardiovascular diseases has been one of the key issues in contemporary life sciences. At a time when the world has entered the era of precision medicine, the diagnosis and prevention of cardiovascular diseases have likewise stepped into a new journey of immunotherapy. At present, in the prevention of such diseases, it is possible to extract relevant body measurements through data mining and then accurately determine the impact of different features on such diseases through the analysis of their different characteristics. This will have a significant positive effect on the prevention of such diseases.

There are many indicators to check whether a patient has cardiovascular disease, the most common being troponin, myoglobin, liver function, kidney function, electrolytes, blood sugar, lipids, cardiac enzymes, and serum cholesterol^46^. When determining whether a patient has a heart condition, medical professionals frequently perform several examinations on the patient's body. As the health condition is variable, this results in a series of data on the laboratory examination sheet that is not constant, and the indicators are fluctuating data. Here, we apply IVFHFSs, which are very appropriate for cardiovascular disease diagnosis, to express the uncertainty of the results of each test. IVFHFSs model is an extension of FHFSs and IVFFSs, inheriting their strengths. That is, the model not only uses interval-valued data to describe MD and ND with a greater range, but it also involves the hesitant data characteristic. In addition to this, when it comes to certain medical situations where there are often multiple medical experts to diagnose the patient, we need to combine the opinions of all of them, and IVFHFSs are undoubtedly very convenient and reasonable.

### Problem description

Three medical experts $E = \left\{ {E_{1} ,E_{2} ,E_{3} } \right\}$ are invited to diagnose five patients $A = \left\{ {A_{1} ,A_{2} ,A_{3} ,A_{4} ,A_{5} } \right\}$ who are potentially suffering from cardiovascular disease. Four indicators that are more likely to influence the diagnosis of cardiovascular disease were selected as attributes for the diagnosis of each candidate, and these were as follows: (1) $B_{1}$ represents blood routine examination; (2) $B_{2}$ represents myocardial enzyme; (3) $B_{3}$ represents rest blood pressure; (4) $B_{4}$ represents serum cholesterol. We give $\omega = \left( {0.2,0.15,0.3,0.35} \right)^{T}$ as the weight vector for each of the above attributes. According to the four attributes, three medical experts give diagnostic assessments of the alternative objects using IVFHFN. In the next section, we give specific steps to identify patients most likely to have cardiovascular disease based on the MAGDM decision method in "Case study and comparative analysis" section.

### Case study demonstration

The evaluation matrixes of three medical experts $D_{5 \times 4}^{m} = \left( {{\mathcalligra{f}}_{ab} } \right)_{5 \times 4}$ ($m = 1,2,3$) are constructed as listed in Tables 1, 2, and 3, respectively.

**Table 1:** IVFHF-DM from first medical expert ${E}_{1}$.

$D_{5 \times 4}^{1}$	$B_{1}$	$B_{2}$	$B_{3}$	$B_{4}$
$A_{1}$	$\left\{ {\left( {\left[ {0.3,0.5\left] , \right[0.6,0.7} \right]} \right)} \right\}$	$\left\{ {\left( {0.4,0.6} \right),\left( {0.5,0.8} \right)} \right\}$	$\{ \left( {\left[ {0.6,0.7\left] , \right[0.5,0.7} \right]} \right),$ $\left( {\left[ {0.5,0.6\left] , \right[0.4,0.7} \right]} \right)\}$	$\left\{ {\left( {\left[ {0.2,0.4\left] , \right[0.7,0.9} \right]} \right)} \right\}$
$A_{2}$	$\{ \left( {\left[ {0.8,0.9\left] , \right[0.1,0.3} \right]} \right),$ $\left( {\left[ {0.7,0.8\left] , \right[0.2,0.3} \right]} \right)\}$	$\{ \left( {\left[ {0.5,0.7\left] , \right[0.3,0.5} \right]} \right),$ $\left( {\left[ {0.8,0.9\left] , \right[0.3,0.4} \right]} \right)\}$	$\left\{ {\left( {\left[ {0.6,0.7\left] , \right[0.2,0.3} \right]} \right)} \right\}$	$\left\{ {\left( {\left[ {0.7,0.9\left] , \right[0.4,0.5} \right]} \right)} \right\}$
$A_{3}$	$\left\{ {\left( {\left[ {0.1,0.2\left] , \right[0.7,0.9} \right]} \right)} \right\}$	$\left\{ {\left( {\left[ {0.2,0.3\left] , \right[0.8,0.9} \right]} \right)} \right\}$	$\left\{ {\left( {\left[ {0.2,0.4\left] , \right[0.5,0.7} \right]} \right)} \right\}$	$\{ \left( {\left[ {0.1,0.2\left] , \right[0.6,0.9} \right]} \right),$ $\left( {\left[ {0.1,0.4\left] , \right[0.8,0.9} \right]} \right)\}$
$A_{4}$	$\left\{ {\left( {\left[ {0.6,0.8\left] , \right[0.5,0.6} \right]} \right)} \right\}$	$\{ \left( {\left[ {0.5,0.8\left] , \right[0.1,0.3} \right]} \right),$ $\left( {\left[ {0.7,0.8\left] , \right[0.2,0.5} \right]} \right)\}$	$\left\{ {\left( {\left[ {0.4.0.5\left] , \right[0.7.0.9} \right]} \right)} \right\}$	$\left\{ {\left( {\left[ {0.3,0.6\left] , \right[0.6,0.7} \right]} \right)} \right\}$
$A_{5}$	$\left\{ {\left( {\left[ {0.3,0.7\left] , \right[0.6,0.7} \right]} \right)} \right\}$	$\left\{ {\left( {\left[ {0.4,0.5\left] , \right[0.7,0.8} \right]} \right)} \right\}$	$\left\{ {\left( {\left[ {0.4.0.5\left] , \right[0.5.0.7} \right]} \right)} \right\}$	$\{ \left( {\left[ {0.7,0.8\left] , \right[0.3,0.4} \right]} \right),$ $\left( {\left[ {0.5,0.8\left] , \right[0.4,0.5} \right]} \right)\}$

**Table 2:** IVFHF-DM from second medical expert ${E}_{2}$.

$D_{5 \times 4}^{2}$	$B_{1}$	$B_{2}$	$B_{3}$	$B_{4}$
$A_{1}$	$\left\{ {\left( {\left[ {0.4,0.5\left] , \right[0.5,0.6} \right]} \right)} \right\}$	$\left\{ {\left( {0.4,0.6} \right),\left( {0.5,0.8} \right)} \right\}$	$\{ \left( {\left[ {0.6,0.7\left] , \right[0.5,0.7} \right]} \right),$ $\left( {\left[ {0.5,0.6\left] , \right[0.4,0.7} \right]} \right)\}$	$\left\{ {\left( {\left[ {0.2,0.4\left] , \right[0.7,0.9} \right]} \right)} \right\}$
$A_{2}$	$\left\{ {\left( {\left[ {0.7,0.8\left] , \right[0.2,0.3} \right]} \right)} \right\}$	$\{ \left( {\left[ {0.5,0.7\left] , \right[0.3,0.5} \right]} \right),$ $\left( {\left[ {0.6,0.8\left] , \right[0.2,0.5} \right]} \right)\}$	$\left\{ {\left( {\left[ {0.6,0.7\left] , \right[0.2,0.3} \right]} \right)} \right\}$	$\left\{ {\left( {\left[ {0.7,0.9\left] , \right[0.4,0.5} \right]} \right)} \right\}$
$A_{3}$	$\left\{ {\left( {\left[ {0.1,0.2\left] , \right[0.7,0.9} \right]} \right)} \right\}$	$\left\{ {\left( {\left[ {0.2,0.3\left] , \right[0.8,0.9} \right]} \right)} \right\}$	$\left\{ {\left( {\left[ {0.1,0.3\left] , \right[0.6,0.7} \right]} \right)} \right\}$	$\left\{ {\left( {\left[ {0.1,0.2\left] , \right[0.6,0.9} \right]} \right) } \right\}$
$A_{4}$	$\{ \left( {\left[ {0.6,0.7\left] , \right[0.4,0.6} \right]} \right),$ $\left( {\left[ {0.6,0.8\left] , \right[0.5,0.6} \right]} \right)\}$	$\left\{ {\left( {\left[ {0.5,0.8\left] , \right[0.1,0.3} \right]} \right)} \right\}$	$\left\{ {\left( {\left[ {0.4.0.5\left] , \right[0.7.0.9} \right]} \right)} \right\}$	$\left\{ {\left( {\left[ {0.3,0.6\left] , \right[0.6,0.7} \right]} \right)} \right\}$
$A_{5}$	$\left\{ {\left( {\left[ {0.3,0.7\left] , \right[0.6,0.7} \right]} \right)} \right\}$	$\left\{ {\left( {\left[ {0.5,0.6} \right],\left[ {0.6,0.8} \right]} \right)} \right\}$	$\left\{ {\left( {\left[ {0.4.0.5\left] , \right[0.5.0.7} \right]} \right)} \right\}$	$\left\{ {\left( {\left[ {0.5,0.8\left] , \right[0.4,0.5} \right]} \right)} \right\}$

**Table 3:** IVFHF-DM from third medical expert ${E}_{3}$.

$D_{5 \times 4}^{3}$	$B_{1}$	$B_{2}$	$B_{3}$	$B_{4}$
$A_{1}$	$\left\{ {\left( {\left[ {0.5,0.6\left] , \right[0.6,0.8} \right]} \right)} \right\}$	$\left\{ {\left( {0.4,0.6} \right),\left( {0.5,0.8} \right)} \right\}$	$\left\{ {\left( {\left[ {0.6,0.7\left] , \right[0.5,0.7} \right]} \right)} \right\}$	$\left\{ {\left( {\left[ {0.2,0.4\left] , \right[0.7,0.9} \right]} \right)} \right\}$
$A_{2}$	$\left\{ {\left( {\left[ {0.8,0.9\left] , \right[0.1,0.3} \right]} \right)} \right\}$	$\{ \left( {\left[ {0.6,0.8\left] , \right[0.2,0.5} \right]} \right),$ $\left( {\left[ {0.8,0.9\left] , \right[0.3,0.4} \right]} \right)\}$	$\left\{ {\left( {\left[ {0.6,0.7\left] , \right[0.2,0.3} \right]} \right)} \right\}$	$\{ \left( {\left[ {0.7,0.9\left] , \right[0.4,0.5} \right]} \right),$ $\left( {\left[ {0.8,0.9} \right],\left[ {0.3,0.4} \right]} \right)\}$
$A_{3}$	$\left\{ {\left( {\left[ {0.1,0.2\left] , \right[0.7,0.9} \right]} \right)} \right\}$	$\left\{ {\left( {\left[ {0.2,0.3\left] , \right[0.8,0.9} \right]} \right)} \right\}$	$\{ \left( {\left[ {0.2,0.4\left] , \right[0.5,0.7} \right]} \right),$ $\left( {\left[ {0.1,0.3\left] , \right[0.6,0.7} \right]} \right)\}$	$\left\{ {\left( {\left[ {0.1,0.4\left] , \right[0.8,0.9} \right]} \right)} \right\}$
$A_{4}$	$\{ \left( {\left[ {0.6,0.7\left] , \right[0.4,0.6} \right]} \right),$ $\left( {\left[ {0.6,0.8\left] , \right[0.5,0.6} \right]} \right)\}$	$\{ \left( {\left[ {0.5,0.8\left] , \right[0.1,0.3} \right]} \right),$ $\left( {\left[ {0.7,0.8\left] , \right[0.2,0.5} \right]} \right)\}$	$\left\{ {\left( {\left[ {0.4.0.5\left] , \right[0.7.0.9} \right]} \right)} \right\}$	$\left\{ {\left( {\left[ {0.3,0.6\left] , \right[0.6,0.7} \right]} \right)} \right\}$
$A_{5}$	$\left\{ {\left( {\left[ {0.3,0.7\left] , \right[0.6,0.7} \right]} \right)} \right\}$	$\left\{ {\left( {\left[ {0.4,0.5\left] , \right[0.7,0.8} \right]} \right)} \right\}$	$\left\{ {\left( {\left[ {0.4.0.5\left] , \right[0.5.0.7} \right]} \right)} \right\}$	$\{ \left( {\left[ {0.7,0.8\left] , \right[0.3,0.4} \right]} \right),$ $\left( {\left[ {0.5,0.8\left] , \right[0.4,0.5} \right]} \right)\}$

*Step 1* According to Tables 1, 2, and 3, we integrate the three IVFHF-DMs so that we can get an integrated IVFHF-DM $D_{5 \times 4}{\prime} = \left( {{\mathcalligra{f}}_{ab}{\prime} } \right)_{5 \times 4}$, as shown in Table 4.

**Table 4:** Integrated IVFHF-DM for all experts.

$D_{5 \times 4}{\prime}$	$B_{1}$	$B_{2}$	$B_{3}$	$B_{4}$
$A_{1}$	$\{ \left( {\left[ {0.3,0.5\left] , \right[0.6,0.7} \right]} \right),$ $\left( {\left[ {0.4,0.5\left] , \right[0.5,0.6} \right]} \right),$ $\left( {\left[ {0.5,0.6\left] , \right[0.6,0.8} \right]} \right)\}$	$\left\{ {\left( {0.4,0.6} \right),\left( {0.5,0.8} \right)} \right\}$	$\{ \left( {\left[ {0.6,0.7\left] , \right[0.5,0.7} \right]} \right),$ $\left( {\left[ {0.5,0.6\left] , \right[0.4,0.7} \right]} \right)\}$	$\left\{ {\left( {\left[ {0.2,0.4\left] , \right[0.7,0.9} \right]} \right)} \right\}$
$A_{2}$	$\{ \left( {\left[ {0.8,0.9\left] , \right[0.1,0.3} \right]} \right),$ $\left( {\left[ {0.7,0.8\left] , \right[0.2,0.3} \right]} \right)\}$	$\{ \left( {\left[ {0.5,0.7\left] , \right[0.3,0.5} \right]} \right),$ $\left( {\left[ {0.6,0.8\left] , \right[0.2,0.5} \right]} \right)$ $\left( {\left[ {0.8,0.9\left] , \right[0.3,0.4} \right]} \right)\}$	$\left\{ {\left( {\left[ {0.6,0.7\left] , \right[0.2,0.3} \right]} \right)} \right\}$	$\{ \left( {\left[ {0.7,0.9\left] , \right[0.4,0.5} \right]} \right),$ $\left( {\left[ {0.8,0.9\left] , \right[0.3,0.4} \right]} \right)\}$
$A_{3}$	$\left\{ {\left( {\left[ {0.1,0.2\left] , \right[0.7,0.9} \right]} \right)} \right\}$	$\left\{ {\left( {\left[ {0.2,0.3\left] , \right[0.8,0.9} \right]} \right)} \right\}$	$\{ \left( {\left[ {0.2,0.4\left] , \right[0.5,0.7} \right]} \right),$ $\left( {\left[ {0.1,0.3\left] , \right[0.6,0.7} \right]} \right)\}$	$\{ \left( {\left[ {0.1,0.2\left] , \right[0.6,0.9} \right]} \right),$ $\left( {\left[ {0.1,0.4\left] , \right[0.8,0.9} \right]} \right)\}$
$A_{4}$	$\{ \left( {\left[ {0.6,0.7\left] , \right[0.4,0.6} \right]} \right),$ $\left( {\left[ {0.6,0.8\left] , \right[0.5,0.6} \right]} \right)\}$	$\{ \left( {\left[ {0.5,0.8\left] , \right[0.1,0.3} \right]} \right),$ $\left( {\left[ {0.7,0.8\left] , \right[0.2,0.5} \right]} \right)\}$	$\left\{ {\left( {\left[ {0.4.0.5\left] , \right[0.7.0.9} \right]} \right)} \right\}$	$\left\{ {\left( {\left[ {0.3,0.6\left] , \right[0.6,0.7} \right]} \right)} \right\}$
$A_{5}$	$\left\{ {\left( {\left[ {0.3,0.7\left] , \right[0.6,0.7} \right]} \right)} \right\}$	$\{ \left( {\left[ {0.5,0.6\left] , \right[0.6,0.8} \right]} \right),$ $\left( {\left[ {0.4,0.5\left] , \right[0.7,0.8} \right]} \right)\}$	$\left\{ {\left( {\left[ {0.4.0.5\left] , \right[0.5.0.7} \right]} \right)} \right\}$	$\{ \left( {\left[ {0.7,0.8\left] , \right[0.3,0.4} \right]} \right),$ $\left( {\left[ {0.5,0.8\left] , \right[0.4,0.5} \right]} \right)\}$

*Step 2* From Table 1, we know that all attributes are the benefit attributes in this case. By Eq. (4.1), we can get the normalized IVFHF-DM $D_{5 \times 4}^{^{\prime\prime}} = \left( {{\mathcalligra{f}}_{ab}^{^{\prime\prime}} } \right)_{5 \times 4}$ which is the same as $D_{5 \times 4}{\prime} = \left( {{\mathcalligra{f}}_{ab}{\prime} } \right)_{5 \times 4}$ in Table 4.

*Step 3* After obtaining the normalized IVFHF-DM, if we do not disregard the weights of the attributes, we use Eq. (3.5) in Theorem 3.3 to aggregate all attributes for each patient. If we consider that each attribute is separately weighted, we use Eq. (3.7) in Theorem 3.4 to aggregate all attributes for each patient. Here, we set the parameters $\sigma =1$ and $\tau =1$, whereupon we can obtain the aggregation results for each diagnostic assessment. The aggregation results of diagnostic assessment are still IVFHFSs, as shown in Tables 5 and 6, respectively.

**Table 5:** Aggregation results based on IVFHFEBM for all experts without attributes weights.

	The aggregated results of the diagnostic assessment
$A_{1}$	$\{ \left( {\left[ {0.4021,0.5660\left] {,,} \right[0.5818,0.7821} \right]} \right),\left( {\left[ {0.4269,0.5660\left] , \right[0.5569,0.7627} \right]} \right),\left( {\left[ {0.4572,0.5907\left] , \right[0.5818,0.8054} \right]} \right),$ $\left( {\left[ {0.3695,0.5343\left] , \right[0.5630,0.7821} \right]} \right),\left( {\left[ {0.3946,0.5343\left] , \right[0.5364,0.7627} \right]} \right),\left( {\left[ {0.4246,0.5603\left] , \right[0.5630,0.8054} \right]} \right)\}$
$A_{2}$	$\{ (\left[ {0.6662,0.8154} \right],\left[ {0.2706,0.4120} \right]{)},\left( {\left[ {0.6840,0.8356} \right],\left[ {0.2402,0.4120} \right]} \right),\left( {\left[ {0.7342,0.8616} \right],\left[ {0.2706,0.3830} \right]} \right),$ $\left( {\left[ {0.6340,0.7852} \right],\left[ {0.2844,0.4120} \right]} \right),\left( {\left[ {0.6533,0.8075} \right],\left[ {0.2574,0.4120} \right]} \right),\left( {\left[ {0.7067,0.8356} \right],\left[ {0.2844,0.3830} \right]} \right),$ $\left( {\left[ {0.6973,0.8154} \right],\left[ {0.2407,0.3830} \right]} \right),\left( {\left[ {0.7134,0.8356} \right],\left[ {0.2112,0.3830} \right]} \right),\left( {\left[ {0.7600,0.8616} \right],\left[ {0.2407,0.3536} \right]} \right),$ $\left( {\left[ {0.6662,0.7852} \right],\left[ {0.2844,0.4120} \right]} \right),\left( {\left[ {0.6533,0.8075} \right],\left[ {0.2574,0.4120} \right]} \right),\left( {\left[ {0.7342,0.8356\left] , \right[0.2547,0.3536} \right]} \right)\}$
$A_{3}$	$\{ \left( {\left[ {0.1591,0.2862} \right],\left[ {0.6624,0.8592} \right]} \right)\left( {\left[ {0.1591,0.3378} \right],\left[ {0.7155,0.8592} \right]} \right),\left( {\left[ {0.1285,0.2554} \right],\left[ {0.6817,0.8592} \right]} \right),$ $\left( {\left[ {0.1285,0.3086} \right],\left[ {0.7320,0.8592} \right]} \right)\}$
$A_{4}$	$\{ \left( {\left[ {0.4669,0.6662\left] , \right[0.5112,0.6726} \right]} \right)\left( {\left[ {0.4669,0.6973} \right],\left[ {0.5389,0.6726} \right]} \right),\left( {\left[ {0.5334,0.6662} \right],\left[ {0.5153,0.6962} \right]} \right),$ $\left( {\left[ {0.5334,0.6973\left] , \right[0.5419,0.6962} \right]} \right)\}$
$A_{5}$	$\{ \left( {\left[ {0.5007,0.6662\left] , \right[0.5187,0.6740} \right]} \right),\left( {\left[ {0.4354,0.6662\left] , \right[0.5329,0.6873} \right]} \right),\left( {\left[ {0.472,0.6468\left] , \right[0.5496,0.6740} \right]} \right),$ $\left( {\left[ {0.4071,0.6468\left] , \right[0.563,0.6873} \right]} \right)\}$

**Table 6:** Aggregation results based on IVFHFEWBM for all experts with attributes weights.

	The aggregated results of the diagnostic assessment
$A_{1}$	$\{ \left( {\left[ {0.2448,0.3494\left] , \right[0.9027,0.9516} \right]} \right),\left( {\left[ {0.2606,0.3494\left] , \right[0.8967,0.9468} \right]} \right),\left( {\left[ {0.2794,0.3641\left] , \right[0.9027,0.9564} \right]} \right),$ $\left( {\left[ {0.2247,0.3279\left] , \right[0.8943,0.9516} \right]} \right),\left( {\left[ {0.2400,0.3279\left] , \right[0.8881,0.9468} \right]} \right),\left( {\left[ {0.2580,0.3428\left] , \right[0.8943,0.9564} \right]} \right)\}$
$A_{2}$	$\{ \left( {\left[ {0.4264,0.5407\left] , \right[0.7693,0.8417} \right]} \right),\left( {\left[ {0.4341,0.5509\left] , \right[0.7584,0.8417} \right]} \right),\left( {\left[ {0.4581,0.5668\left] , \right[0.7693,0.8357} \right]} \right),$ $\left( {\left[ {0.4520,0.5407\left] , \right[0.7544,0.8303} \right]} \right),\left( {\left[ {0.4591,0.5509\left] , \right[0.7432,0.8303} \right]} \right),\left( {\left[ {0.4819,0.5668\left] , \right[0.7544,0.8242} \right]} \right),$ $\left( {\left[ {0.4323,0.5173\left] , \right[0.7748,0.8303} \right]} \right),\left( {\left[ {0.4403,0.5287\left] , \right[0.7639,0.8303} \right]} \right),\left( {\left[ {0.4652,0.5463\left] , \right[0.7748,0.8242} \right]} \right),$ $\left( {\left[ {0.4067,0.5173\left] , \right[0.7890,0.8417} \right]} \right),\left( {\left[ {0.4152,0.5287\left] , \right[0.7784,0.8417} \right]} \right),\left( {\left[ {0.4409,0.5463\left] , \right[0.7890,0.8357} \right]} \right)\}$
$A_{3}$	$\{ \left( {\left[ {0.0968,0.1772\left] , \right[0.9129,0.9658} \right]} \right),\left( {\left[ {0.0968,0.2199\left] , \right[0.9301,0.9658} \right]} \right),\left( {\left[ {0.0770,0.1573\left] , \right[0.9205,0.9658} \right]} \right),$ $\left( {\left[ {0.0770,0.1981\left] , \right[0.9366,0.9658} \right]} \right)\}$
$A_{4}$	$\{ \left( {\left[ {0.2789,0.4060\left] , \right[0.8632,0.9223} \right]} \right),\left( {\left[ {0.3105,0.4060\left] , \right[0.8767,0.9329} \right]} \right),\left( {\left[ {0.2789,0.4248\left] , \right[0.8705,0.9223} \right]} \right),$ $\left( {\left[ {0.3105,0.4248\left] , \right[0.8837,0.9329} \right]} \right)\}$
$A_{5}$	$\{ \left( {\left[ {0.3179,0.4248\left] , \right[0.8691,0.9121} \right]} \right),\left( {\left[ {0.2732,0.4248\left] , \right[0.8799,0.9203} \right]} \right),\left( {\left[ {0.3045,0.4159\left] , \right[0.8739,0.9121} \right]} \right),$ $\left( {\left[ {0.2602,0.4159\left] , \right[0.8845,0.9203} \right]} \right)\}$

*Step 4* We separately obtain Tables 7 and 8 based on the aggregation results of Tables 5 and 6 and the patient score values calculated by SC (Eq. (2.1) and Eq. (2.2)).

**Table 7:** Patients’ score values without attributes weights.

	$A_{1}$	$A_{2}$	$A_{3}$	$A_{4}$	$A_{5}$
Score	$\left[ { - 0.205, - 0.002} \right]$	$\left[ {0.139,0.272} \right]$	$\left[ { - 0.316, - 0.157} \right]$	$\left[ { - 0.097,0.085} \right]$	$\left[ { - 0.11,0.062} \right]$

**Table 8:** Patients’ score values with attributes weights.

	$A_{1}$	$A_{2}$	$A_{3}$	$A_{4}$	$A_{5}$
Score	$\left[ { - 0.423, - 0.34} \right]$	$\left[ { - 0.246, - 0.147} \right]$	$\left[ { - 0.45, - 0.392} \right]$	$\left[ { - 0.386, - 0.297} \right]$	$\left[ { - 0.372, - 0.3} \right]$

*Step 5* According to the score values displayed in Tables 7 and 8, we can ultimately obtain the sorting result of the five patients as ${A}_{2}>{A}_{4}>{A}_{5}>{A}_{1}>{A}_{3}$ when we do not consider the attributes weights and the sorting result of the five patients is ${A}_{2}>{A}_{5}>{A}_{4}>{A}_{1}>{A}_{3}$ when we consider the attributes weights. The sorted results reveal that, according to the medical expert's diagnostic analysis, patient ${A}_{2}$ is the most likely to suffer from cardiovascular disease, and patient ${A}_{3}$ is the least likely to have cardiovascular disease. That is, ${A}_{2}$ has the greatest degree of urgency and is in the most need of treatment for this heart condition.

### Sensitivity analysis

In this part, we discuss the effect of different values of $\sigma$ and $\tau$ for different parameters on the ranking results of the alternatives from ${A}_{1}$ to ${A}_{5}$ by providing varied values of $\sigma$ and $\tau$. Then, Table 9 presents the score values and ranking results from ${A}_{1}$ to ${A}_{5}$ based on the IVFHFEBM operator. Table 10 presents the score values and ranking results from ${A}_{1}$ to ${A}_{5}$ based on the IVFHFEWBM operator.

**Table 9:** The score values for alternatives as parameters vary based on the IVFHFEBM operator.

Parameters	${A}_{1}$	${A}_{2}$	${A}_{3}$	${A}_{4}$	${A}_{5}$	Ranking
$\sigma =1,$ $\tau =1$	$[-0.205,$ $-0.002]$	$[0.139,$ $0.272]$	$[ - 0.316,$ $- 0.157]$	$[ - 0.097,$ $0.085]$	$[ - 0.11,$ $0.062]$	$A_{2} > A_{4} > A_{5} > A_{1} > A_{3}$
$\sigma = 1,$ $\tau = 0$	$[ - 0.193,$ $0.007]$	$[0.147,$ $0.283]$	$[ - 0.306,$ $- 0.146]$	$[ - 0.059,$ $0.129]$	$[ - 0.087,$ $0.08]$	$A_{2} > A_{4} > A_{5} > A_{1} > A_{3}$
$\sigma = 1,$ $\tau = 3$	$[ - 0.174,$ $0.014]$	$[0.157,$ $0.291]$	$[ - 0.285,$ $- 0.071]$	$[ - 0.050,$ $0.119]$	$[ - 0.079,$ $0.093]$	$A_{2} > A_{4} > A_{5} > A_{1} > A_{3}$
$\sigma = 0,$ $\tau = 3$	$[ - 0.152,$ $0.028]$	$[0.171,$ $0.304]$	$[ - 0.259,$ $- 0.130]$	$[ - 0.01,$ $0.166]$	$[ - 0.047,$ $0.121]$	$A_{2} > A_{4} > A_{5} > A_{1} > A_{3}$
$\sigma = 0,$ $\tau = 1$	$[ - 0.193,$ $0.007]$	$[0.147,$ $0.283]$	$[ - 0.306,$ $- 0.146]$	$[ - 0.059,$ $0.129]$	$[ - 0.087,$ $0.08]$	$A_{2} > A_{4} > A_{5} > A_{1} > A_{3}$
$\sigma = 3,$ $\tau = 1$	$[ - 0.174,$ $0.014]$	$[0.157,$ $0.291]$	$[ - 0.285,$ $- 0.071]$	$[ - 0.050,$ $0.119]$	$[ - 0.079,$ $0.093]$	$A_{2} > A_{4} > A_{5} > A_{1} > A_{3}$
$\sigma = 3,$ $\tau = 0$	$[ - 0.152,$ $0.028]$	$[0.171,$ $0.304]$	$[ - 0.259,$ $- 0.130]$	$[ - 0.01,$ $0.166]$	$[ - 0.047,$ $0.121]$	$A_{2} > A_{4} > A_{5} > A_{1} > A_{3}$
$\sigma = 3,$ $\tau = 3$	$[ - 0.172,$ $0.015]$	$[0.158,$ $0.293]$	$[ - 0.3,$ $- 0.141]$	$[ - 0.042,$ $0.12]$	$[ - 0.086,$ $0.093]$	$A_{2} > A_{4} > A_{5} > A_{1} > A_{3}$

**Table 10:** The score values for alternatives as parameters vary based on the IVFHFEWBM operator.

Parameters	$A_{1}$	$A_{2}$	$A_{3}$	$A_{4}$	$A_{5}$	Ranking
$\sigma = 1,$ $\tau = 1$	$[ - 0.423,$ $- 0.336]$	$[ - 0.246,$ $- 0.147]$	$[ - 0.45,$ $- 0.392]$	$[ - 0.386,$ $- 0.297]$	$[ - 0.372,$ $- 0.3]$	$A_{2} > A_{5} > A_{4} > A_{1} > A_{3}$
$\sigma = 1,$ $\tau = 0$	$[ - 0.419,$ $- 0.336]$	$[ - 0.241,$ $- 0.139]$	$[ - 0.446,$ $- 0.388]$	$[ - 0.384,$ $- 0.295]$	$[ - 0.361,$ $- 0.287]$	$A_{2} > A_{5} > A_{4} > A_{1} > A_{3}$
$\sigma = 1,$ $\tau = 3$	$[ - 0.410,$ $- 0.325]$	$[ - 0.224,$ $- 0.128]$	$[ - 0.436,$ $- 0.374]$	$[ - 0.375,$ $- 0.287]$	$[ - 0.336,$ $- 0.256]$	$A_{2} > A_{5} > A_{4} > A_{1} > A_{3}$
$\sigma = 0,$ $\tau = 3$	$[ - 0.398,$ $- 0.311]$	$[0.139,$ $- 0.272]$	$[ - 0.421,$ $- 0.36]$	$[ - 0.367,$ $- 0.279]$	$[ - 0.3,$ $- 0.219]$	$A_{2} > A_{5} > A_{4} > A_{1} > A_{3}$
$\sigma = 0,$ $\tau = 1$	$[ - 0.419,$ $- 0.336]$	$[ - 0.241,$ $- 0.139]$	$[ - 0.446,$ $- 0.388]$	$[ - 0.384,$ $- 0.295]$	$[ - 0.361,$ $- 0.287]$	$A_{2} > A_{5} > A_{4} > A_{1} > A_{3}$
$\sigma = 3,$ $\tau = 1$	$[ - 0.410,$ $- 0.325]$	$[ - 0.224,$ $- 0.128]$	$[ - 0.436,$ $- 0.374]$	$[ - 0.375,$ $- 0.287]$	$[ - 0.336,$ $- 0.256]$	$A_{2} > A_{5} > A_{4} > A_{1} > A_{3}$
$\sigma = 3,$ $\tau = 0$	$[ - 0.398,$ $- 0.311]$	$[0.139,$ $- 0.272]$	$[ - 0.421,$ $- 0.36]$	$[ - 0.367,$ $- 0.279]$	$[ - 0.3,$ $- 0.219]$	$A_{2} > A_{5} > A_{4} > A_{1} > A_{3}$
$\sigma = 3,$ $\tau = 3$	$[ - 0.412$ $- 0.329]$	$[ - 0.222,$ $- 0.122]$	$[ - 0.441,$ $- 0.373]$	$[ - 0.373,$ $- 0.287]$	$[ - 0.346,$ $- 0.261]$	$A_{2} > A_{5} > A_{4} > A_{1} > A_{3}$

As shown in Tables 10 and 11, we can notice that when the values of the $\sigma$ and $\tau$ parameters are adjusted, the score values from $A_{1}$ to $A_{5}$ are changed accordingly. In particular, we discover that the interrelationships between different attributes are not considered when $\sigma = 0$ or $\tau = 0$. This is determined by the properties of the BM operator itself. Specially, we note that when the values of the $\sigma$ and $\tau$ parameters are exchanged, the aggregation results and the eventual score values are calculated in the same way due to the equal status of the $\sigma$ and $\tau$ parameters during the computational process of the BM operator. Moreover, when the values of $\sigma$ and $\tau$ are equal, as the values of $\sigma$ and $\tau$ increase simultaneously, the score values from $A_{1}$ to $A_{5}$ increase simultaneously. When keeping $\sigma = 1$ or $\tau = 1$ constant, the score values from $A_{1}$ to $A_{5}$ increase with the value of $\sigma$ or $\tau$ regardless of this special case of $\sigma = 0$ and $\tau = 0$.

**Table 11:** Comparison analysis with existing methods.

Methods	Operators	Ranking
The proposed method	IVFHFEBM ($\sigma ,\tau = 1$)	$A_{2} > A_{4} > A_{5} > A_{1} > A_{3}$
	IVFHFEWBM ($\sigma ,\tau = 1$)	$A_{2} > A_{5} > A_{4} > A_{1} > A_{3}$
The method of Kirişci et al.^22^	IVFHFWA	$A_{2} > A_{5} > A_{4} > A_{1} > A_{3}$
	IVFHFWG	$A_{2} > A_{5} > A_{4} > A_{1} > A_{3}$
The method of Zeng et al.^44^	WIVHFWA	$A_{2} > A_{4} > A_{5} > A_{1} > A_{3}$
	WIVHFWG	$A_{2} > A_{4} > A_{5} > A_{1} > A_{3}$
The method of Ankara et al.^43^	Correlation coefficient I (KK I)	$A_{2} > A_{5} > A_{4} > A_{1} > A_{3}$
	Correlation coefficient II (KK II)	$A_{2} > A_{5} > A_{4} > A_{1} > A_{3}$

So, we can adjust the values of $\sigma$ and $\tau$ to change the curves of data and still keep the final result the same, that $A_{2}$ is the most likely to suffer from cardiovascular disease and patient $A_{3}$ is the least likely to suffer from cardiovascular disease. The above analyses amply demonstrate that our approach is highly flexible and robust.

In general, the values of the $\sigma$ and $\tau$ parameters do not affect our selection of the most likely to have cardiovascular disease, and $A_{2}$ is consistently the best option.

### Comparative analysis

To confirm the efficacy of the suggested method, we handle the aforementioned scenario using existing MAGDM methods and perform a comparison study. The existing MAGDM methods based on six sort methods: the interval-valued Fermatean hesitant fuzzy weighted averaging(IVFHFWA) operator and interval-valued Fermatean hesitant fuzzy weighted geometric(IVFHFWG) operator proposed by Kirişci et al.^22^, the weighted interval-valued hesitant fuzzy weighted averaging(WIVHFWA) operator and weighted interval-valued hesitant fuzzy weighted geometric(WIVHFWG) operator proposed by Zeng et al.^44^, the correlation coefficient I(KK I) and correlation coefficient(KK II) operator proposed by Ankara et al.^43^. In order to reflect the properties of the BM operator, which can consider the connection between attributes, the parameters $\sigma , \tau$ of the IVFHFEBM operator and IVFHFEWBM operator are both defined as 1. Table 11 presents the comparison outcomes.

Based on the ranking results in Table 11, We can see that the ranking results of $A_{4}$ and $A_{5}$ differ in the two AOs mentioned. Using IVFHFEBM operators, $A_{4}$ is ranked second, and $A_{5}$ is ranked third. However, regarding the IVFHFEWBM operator, $A_{5}$ is ranked second, and $A_{2}$ is ranked third. This is because the IVFHFEWBM operator considers the weight factor of attributes. In addition, we find that our proposed method based on IVFHFEBM agrees with the sorting results of Zeng et al.^44^ $A_{2} > A_{4} > A_{5} > A_{1} > A_{3}$, and our proposed method based on IVFHFEWBM agrees with the sorting results of Kirişci et al.^22^ and Ankara et al.^43^ $A_{2} > A_{5} > A_{4} > A_{1} > A_{3}$. Not only that, the results of all methods are $A_{2}$ ranked highest and $A_{3}$ ranked lowest. The above conclusions fully prove that our proposed method is correct and effective.

### The advantages compared to existing methods

#### Comparison of the advantages of the data model with existing methods

IVFHFSs are the latest proposed FSs data model, which is an extension of FHFSs and IVFFSs, inheriting their respective advantages. In other words, IVFHFSs incorporate the hesitating feature of data in addition to using interval-valued data to characterize MD and ND with a greater range. In detail, the feature of interval value allows it to better handle the fluctuation of data, and the feature of hesitance allows it to better retain the data and reduce the loss of information when facing the group decision-making model, and the feature of FFSs allows it to have a wider range. IVFHFSs also apply MD and ND to depict uncertainty, while IVHFSs in^44^ ignore ND. Therefore, when dealing with fuzzy information, it has the advantage of being able to represent fuzzy information more flexibly than other fuzzy sets and represents fuzzy information in a wider range.

#### The advantages of our proposed AOs

1. Considering the connection between attributes.

In real life, especially in the context of medical diagnosis, the individual attributes are often linked to each other. For example, high blood sugar and high lipids often co-exist, especially in diabetic patients. In addition, elevated levels of myoglobin and troponin may be associated with myocardial damage, whereas myocardial damage may also lead to abnormal changes in blood sugar and lipids. The IVFHFEBM and IVFHFEWBM operators take into account the connection between the attributes, while the existing approaches in^22,44^, and^43^ ignore the relationship between attributes. Therefore, our method is superior compared to existing AOs.

(2) Higher flexibility.

Furthermore, the IVFHFEBM and IVFHFEWBM operators have two parameters, $\sigma$ and $\tau$. We can modify the values of $\sigma$ and $\tau$ to adjust the change in the data and still keep the final result constant. That is, our proposed method has two parameters, and by adjusting them, we can obtain the form of AO we need. Thus, compared with the other three methods in^22,44^ and^43^ that do not have adjustable parameters, our proposed method is more flexible and ingenious than them.

In the following, the differences between our method and the other three MAGDM will be compared. Meanwhile, the merits of our method based on the above discussions are summarized below.

In detail, we introduce the differences and comparisons among diverse MAGDM methods from seven perspectives, and the results are listed in Table 12.

**Table 12:** Comparison of MAGDM methods.

Methods	Data model	Arithmetic operation	Connection between attributes	Parameter number	Flexibility
The method of Zeng et al.^44^	IVHFSs	A-TNs	×	Zero	Lower
The method of Kirişci et al.^22^	IVFHFSs	A-TNs	×	Zero	Lower
The method of Ankara et al.^43^	IVFHFSs	–	×	Zero	Lower
The proposed method	IVFHFSs	E-TNs	√	Two	Higher

It is obvious from Table 12 that our proposed approach is superior to the three existing methods.

## Conclusion

IVFHFSs combine the characteristics of IVHFSs and FFSs and can deal with uncertainty more effectively and extensively. This paper presents a novel MAGDM method under the IVFHFSs. Firstly, we study the operational laws based on E-TNs in depth. Secondly, we apply these operational laws to propose the IVFHFEBM operator and the IVFHFEWBM operator in turn. The proof procedure for the AOs and the related corollaries are also given in detail. Unlike the existing methods, our proposed AOs take into account the link between attributes. Moreover, In the context of cardiovascular disease diagnosis, the fitness of IVFHFSs in dealing with the complex and uncertain MAGDM problem is illustrated. Subsequently, the effectiveness and robustness of the proposed method are verified by sensitivity analysis and comparative analysis. Finally, the advantages of our method are summarized and refined in comparison with existing MAGDM.

In future work, we will explore a method that can objectively calculate weights to reduce subjective factors in making decisions under the IVFHFSs, thus ensuring the validity and reasonableness of the weights of decision results. In addition to this, we will investigate a decision method based on IVFHFSs that has not only a ranking function but also a classification function.

## Data Availability

The datasets used and/or analyzed during the current study available from the corresponding author on reasonable request.
